# Reassessing adult surfactant replacement therapy with mechanics-informed reinforcement learning

**DOI:** 10.1371/journal.pcbi.1014629

**Published:** 2026-08-18

**Authors:** Philippe Meliga, Gregor Roncin, Alejandro Yepes Peñaranda, Elie Hachem

**Affiliations:** Mines Paris, PSL University, Centre for Material Forming (CEMEF), UMR CNRS, Sophia Antipolis, France; University of Connecticut School of Medicine, UNITED STATES OF AMERICA

## Abstract

Surfactant replacement therapy (SRT) remains clinically limited to neonatal applications, in part because the mechanical feasibility of achieving efficient delivery in adult lungs is poorly understood. Previous computational studies have largely been descriptive or based on parameter sweeps, providing limited guidance on how to design efficient adult protocols under anatomical constraints. Here, we introduce a computational framework that integrates mechanistic modeling of surfactant propagation in anatomically motivated airway trees with deep reinforcement learning (DRL) to identify efficient delivery strategies across scales and airway geometries. The approach leverages a reduced-order model of plug transport and redistribution that captures the key mechanics of surfactant coating in complex airway networks while remaining lightweight enough for large-scale optimization. A custom DRL agent autonomously explores delivery parameters—including aliquot volume, flow rate, patient posture, and surfactant properties—to optimize protocol-level performance across diverse anatomical and physiological conditions. Under the branch-level coverage-based reward adopted here, systematic optimization of delivery parameters improves distal delivery at both pediatric and adult scales, while clarifying how these gains depend on prescribed volume, airway asymmetry, and control complexity. Increasing the number of aliquots improves access to distal regions and, in favorable geometries, shifts the onset of high-coverage regimes toward lower prescribed volumes, whereas posture becomes especially informative in asymmetric trees. Extending the control space to include surfactant rheology provides an additional lever, particularly in constrained adult settings, but does not overcome the structural limitations imposed by strong geometric asymmetry. Overall, these results establish a physics-based framework for AI-assisted optimization of intrapulmonary liquid delivery and clarify the respective roles of dose partitioning, posture, and formulation tuning. They also show that the interpretation of delivery success depends strongly on the evaluation metric: branch-level coverage provides a functionally oriented measure across heterogeneous airway trees, whereas stricter homogeneity metrics yield substantially more pessimistic assessments, especially in adult asymmetric geometries. These findings do not predict clinical efficacy; rather, they provide a controlled mechanistic feasibility benchmark and testable design hypotheses within physiologically realistic bounds.

## Author summary

Surfactant replacement therapy (SRT), in which a liquid surfactant is delivered directly into the lungs, is highly effective in premature infants but has repeatedly failed in adults with acute respiratory distress syndrome. One common explanation is that the adult lung is simply too large and too irregular for the liquid to spread efficiently. We tested this idea using a computer model that simulates how surfactant plugs move, split, and coat the airways, combined with an artificial-intelligence method that learns which delivery protocols work best. We examined how adjustable factors such as dose splitting, instillation flow rate, body position, and surfactant properties affect delivery in pediatric and adult airway trees. Our results show that delivery performance depends strongly on airway geometry and on how success is measured. In particular, some classical global uniformity measures can yield dramatically more pessimistic assessments of adult delivery, especially in asymmetric trees. By contrast, branch-level coverage provides a more functionally oriented picture of how broadly surfactant reaches the distal lung. Overall, the study identifies which protocol choices most improve delivery, where strong geometric limitations remain, and how mechanical strategy and formulation interact. These findings do not demonstrate clinical benefit and should not be read as candidate clinical protocols. Nonetheless, they suggest that some assumed mechanical constraints on adult SRT deserve re-examination, and provide computational design hypotheses to guide targeted experimental studies in controlled settings.

## Introduction

Surfactant replacement therapy (SRT) has long been a cornerstone of neonatal care, dramatically improving survival in preterm infants with respiratory distress syndrome by restoring pulmonary surface activity [1]. However, its translation to conditions involving surfactant deficiency beyond the neonatal setting has been limited [2]. In particular, SRT is generally considered clinically ineffective for adult acute respiratory distress syndrome (ARDS), a severe inflammatory disorder with persistently high mortality. This conclusion is supported by randomized trials [3–6] and meta-analyses [7,8] showing no mortality benefit of exogenous surfactant in adult ARDS, and no consistent improvement in oxygenation.

While the obstacles to effective adult SRT remain debated [9,10], a prevailing assumption is that conventional SRT is strongly constrained by delivery maldistribution in the large, heterogeneous adult airway network. Targeted bronchoscopic delivery explored during the COVID-19 pandemic has further renewed interest in this mechanical question, with early reports suggesting feasibility and possible signals of benefit [11]. In this context, the present study sets aside biochemical and biological dimensions (e.g., rapid biophysical inactivation and persistence of the inflammatory milieu) to focus on the mechanical constraints governing delivery at adult scale. Accordingly, deposition improvements in this *in silico* model should be interpreted as indicators of mechanical feasibility rather than as clinical outcome surrogates. The goal is to map attainable surfactant–delivery regimes under physically realistic conditions within the present model, thereby helping distinguish intrinsic mechanical limitations from controllable levers to be further investigated in targeted experimental or model-extension studies.

Over the past two decades, simplified physics-based models of air–liquid interface propagation in idealized pulmonary geometries [12–15] have offered valuable mechanistic insight into how surfactant spreads through the bronchial tree. These models identified key nonlinear processes—wall coating losses, uneven plug splitting, and gravitational effects—that govern delivery efficiency and spatial uniformity across airway generations [16–18]. When applied recursively throughout the branching structure, these formulations yield tractable organ-scale descriptions of SRT that capture how geometry-dependent mechanisms shape global delivery performance and have reproduced key qualitative contrasts between neonatal and adult SRT outcomes [19–21].

Despite these advances, translating mechanistic understanding into experimentally testable and clinically relevant delivery strategies remains an open challenge. Most existing models are primarily explanatory rather than prescriptive: they describe how surfactant behaves, but offer limited guidance on how controllable delivery parameters should be tuned to achieve broad and efficient distal deposition. As a result, adult SRT design still relies largely on empirical adjustments rather than systematic, physics-informed optimization [8,22].

This work addresses the lack of systematic, physics-informed design tools for SRT by introducing a numerical framework that integrates mechanistic modeling with AI-guided optimization to identify and refine efficient delivery strategies across airway scales. Rather than scanning parameters, our framework performs protocol-level optimization by learning candidate delivery strategies directly from a mechanistic simulator to meet clinically motivated delivery objectives. This is achieved with reinforcement learning (RL), a paradigm increasingly effective for data-driven control in fluid mechanics, capable of discovering unanticipated yet effective control policies even when direct optimization of the governing equations is computationally prohibitive [23,24]. RL-based optimization studies in biomedical fluid mechanics have predominantly targeted cardiovascular applications, often relying on electrical-analog (lumped-parameter) circulation surrogates to optimize global hemodynamic objectives (e.g., cardiac output, arterial pressures) through device or operating parameters [25–27]. Fewer works couple RL to high-fidelity CFD, for instance in shape-control settings for upper-airway surgical planning [28] or device/geometry optimization in vascular flows (e.g., aneurysm hemodynamics [29]).

To our knowledge, this is the first RL-based framework for lower-airway instillation/delivery fluid mechanics and, in particular, for surfactant delivery optimization, trained on a simplified yet mechanistically faithful organ-scale SRT model that captures key delivery challenges while remaining computationally efficient for large-scale exploration. The model has been extensively validated against *in vitro* data across realistic flow regimes [16,17] and benchmarked against whole-lung *ex vivo* rat studies [21], showing that the resulting deposition patterns and flow transitions are physiologically consistent. Building on this foundation, the study focuses on methodological feasibility by integrating this physics-based formulation with a neural-network–based RL implementation (hereafter referred to as deep RL, DRL) that autonomously identifies efficient and physiologically meaningful delivery strategies across anatomically motivated, synthetic airway-tree geometries (symmetric and stochastically perturbed variants).

Within this framework, the agent explores delivery parameters under varying anatomical and physiological conditions and is trained with a clinically motivated reward function to identify mechanically efficient delivery patterns. A central element of the approach is the design of the reward function itself, which must ensure stable learning while faithfully encoding delivery objectives so that optimization aligns with therapeutic intent rather than purely algorithmic performance. While DRL has primarily been used for real-time closed-loop control via actuation/feedback [30], our framing emphasizes prescriptive protocol design under anatomical constraints. We therefore employ a custom single-step DRL algorithm—a stateless variant tailored to open-loop control [31–35], in which network updates occur after one-step episodes [36].

The study investigates how protocol design and control complexity influence distal delivery, volumetric efficiency, and redistribution patterns in pediatric and adult airway models. Multi-aliquot scenarios are organized by increasing levels of realism and control freedom, from clinically inspired baseline protocols to configurations that allow per-aliquot adjustment of dose partitioning, flow rate, posture, and, in the most flexible setting, surfactant rheology. Specifically, we (i) formulate surfactant instillation as an open-loop protocol-design problem optimized by a single-step DRL agent interacting with a validated reduced-order mechanistic model; (ii) quantify the marginal value of clinically motivated control levers across anatomically motivated airway-tree geometries; and (iii) introduce a branch-level coverage-based evaluation framework that assesses whether delivery remains locally acceptable across a broad distal population in heterogeneous trees, while exposing the limitations of previously used metrics. Rather than asking whether adult delivery can simply replicate pediatric outcomes, this framework maps the mechanically attainable delivery landscape within the present modeling envelope and helps distinguish intrinsic geometric barriers from losses that can be mitigated through protocol design. We do not represent mucus, compliance/recruitment, or fully coupled ventilation–liquid interactions; feasibility conclusions should therefore be interpreted within this modeling envelope, and sensitivity to these mechanisms is left to targeted extensions and prospective experimental checks.

## Materials and methods

### SRT modeling framework

#### Geometry generation and structural parameters.

**Fig 1:**
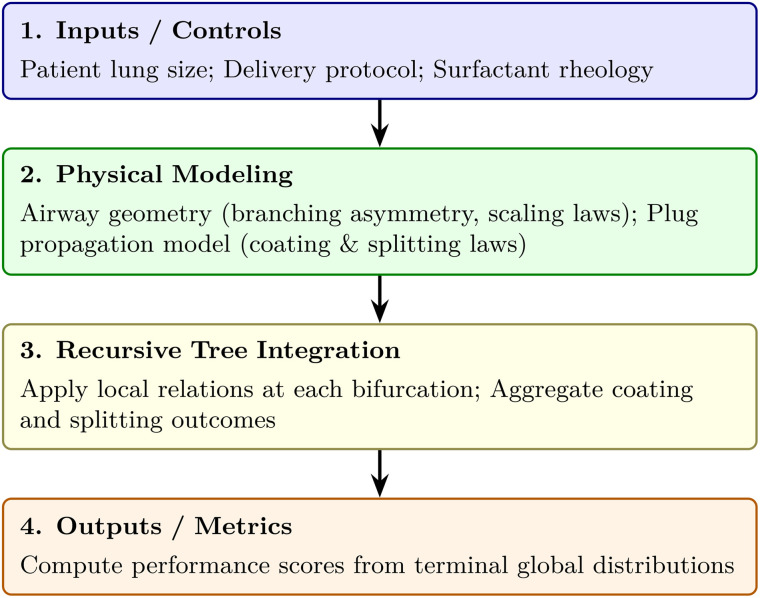
Schematic overview of the SRT modeling framework illustrating the links between input controls, physical modeling, recursive tree integration, and output metrics.

The model initializes airway trees that are designed to be morphometrically plausible and consistent with reported scaling laws of the human bronchial network. Each tree is defined by the branching structure, geometric scaling laws, and gravitational orientation, which jointly determine the local flow conditions governing surfactant propagation (Fig 1). Each branch is modeled as a rigid cylindrical duct with constant diameter, and the length-to-diameter ratio is uniformly set to 2.8, consistent with average morphometric data [37,38].

The branching geometry follows a Murray-type (Hess–Murray) design rule: daughter airway diameters are computed as $a_{1}=r^{1/3}a_{0}$ and $a_{2}=(1-r{)}^{1/3}a_{0}$, where *r* is the division ratio that defines the relative diameters of the two daughter branches with respect to the parent airway, and the exponent follows Murray’s minimum-work scaling [39]. The bifurcation angles $\theta_{1}$ and $\theta_{2}$ of the daughter branches are defined after [40] as


$$\begin{array}{c} \cos\theta_{1}=\frac{1+r^{4/3}-(1-r{)}^{4/3}}{2r^{2/3}},\;\cos\theta_{2}=\frac{1-r^{4/3}+(1-r{)}^{4/3}}{2(1-r{)}^{2/3}}\;, \end{array}$$ (1)

to minimize the total volume of the bifurcating structure, and the rotation angles between successive bifurcation planes are set to $90^{\circ }$.

**Fig 2:**
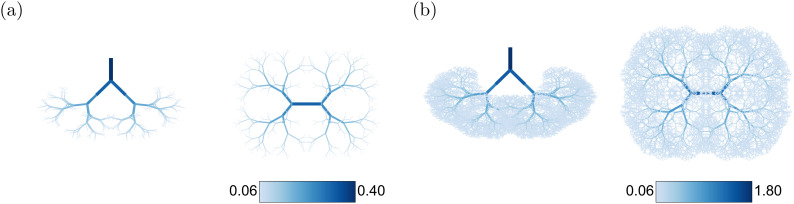
Reference symmetric pediatric and adult airway-tree models. (a) From left to right: side and bottom views of the (symmetric) pediatric airway tree (8 generations plus trachea; 256 terminal airways). (b) Same as (a) for the (symmetric) adult airway tree (15 generations plus trachea; 32,768 terminal airways). Airway narrowing across generations is shown by encoding branch diameters with both color and line thickness. For ease of comparison, branch lengths and diameters are normalized by the tracheal diameter of each model.

Unless specified otherwise, the symmetric configuration *r* = 0.5 is assumed, corresponding to the canonical Weibel-C model [41]. In this case, the two daughter branches emerge symmetrically from the parent airway, with equal and opposite branching angles ($|\theta_{i}|=37.6^{\circ }$), and the *i* -th generation (with the trachea as the 0-th generation) follows $a(i)=a_{0}/2^{i/3}$. As a result, the symmetric airway tree morphology is fully determined by the tracheal diameter and the division ratio. Representative airway trees shown in Fig 2 are used to model both pediatric and adult lungs. The pediatric model comprises 8 generations with a tracheal diameter of 4 mm, whereas the adult model consists of 15 generations with a tracheal diameter of 18 mm, both consistent with typical morphometric data. The resulting terminal airway counts are 256 for the pediatric model and 32,768 for the adult model.

To incorporate natural asymmetry between daughter branches, asymmetric airway trees are generated by sampling an asymmetry coefficient using a stochastic scheme and mapping it to a local division ratio *r* that varies across bifurcations. Either daughter can therefore be the larger one (depending on whether *r* > 0.5 or *r* < 0.5), and diameters compound multiplicatively along any root-to-terminal path as the local rule is applied recursively. Fixed pseudo-random seeds ensure reproducible anatomical variability across experiments. In this work, asymmetry is treated as a sensitivity axis within a proof-of-feasibility scope, rather than as a statistical sample of population-level anatomical variability. Symmetric trees serve as a reference to characterize baseline behaviors, whereas mildly and highly asymmetric trees are used to probe sensitivity to branching imbalance. We use five stochastic realizations per asymmetry level, a limited sampling intended to illustrate trend consistency and anatomy-driven variability, not population-level generalization.

**Fig 3:**
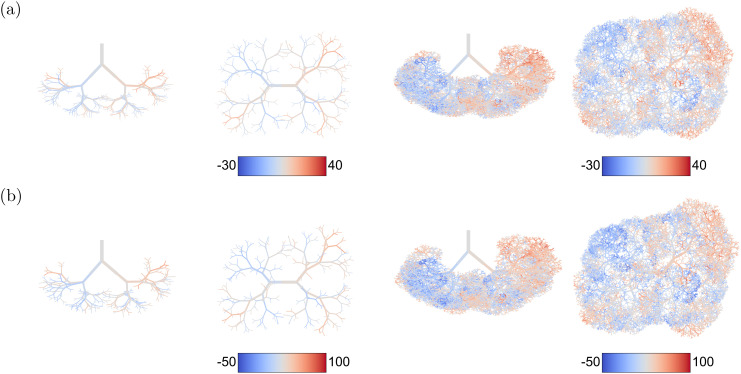
Mildly (a) and highly (b) asymmetric airway trees generated from the same pseudo-random seed (tree 3 in Table 1). For each asymmetry level, the pediatric model (left; 8 generations plus trachea) and the adult model (right; 15 generations plus trachea) are shown in side and bottom views. Airway narrowing across generations is encoded by line thickness, while color encodes the relative difference between the local diameter and that of the corresponding symmetric tree. For ease of comparison, all dimensions are normalized by the tracheal diameter of each model.

Table 1 summarizes the symmetric baseline and the mildly and highly asymmetric pediatric and adult airway trees used in this study. The mean terminal diameters remain identical to those of the symmetric reference cases (0.63 mm for pediatric and 0.56 mm for adult models), ensuring comparable distal size scales across models. Increasing asymmetry primarily broadens the distribution, with standard deviations reaching approximately 0.10–0.12 mm in highly asymmetric configurations. Fig 3 shows representative mildly and highly asymmetric configurations generated from the same pseudo-random seed (tree 3 in Table 1). Complete visualizations for all seeds and the full sampling procedure are provided in S1 Text (Section 1).

**Table 1:** Statistics of the symmetric and asymmetric lung models used in the numerical experiments. Pediatric and adult models comprise 8 and 15 generations plus trachea, respectively. Trees with the same ID (1–5) correspond to different numbers of generations for the same pseudo-random seed.

Model	Group	Tracheal Ø [ mm]	Tree	Terminal Ø [ mm]
Sym.	Pediatric	4	—	0.63
	Adult	18	—	0.56
Mild asym.	Pediatric	4	1	0.53 – 0.76
			2	0.50 – 0.76
			3	0.51 – 0.76
			4	0.51 – 0.76
			5	0.52 – 0.75
	Adult	18	1	0.37 – 0.83
			2	0.37 – 0.82
			3	0.38 – 0.82
			4	0.37 – 0.83
			5	0.36 – 0.81
High asym.	Pediatric	4	1	0.44 – 0.90
			2	0.38 – 0.91
			3	0.41 – 0.90
			4	0.41 – 0.91
			5	0.41 – 0.89
	Adult	18	1	0.24 – 1.19
			2	0.24 – 1.18
			3	0.25 – 1.15
			4	0.23 – 1.18
			5	0.22 – 1.14

#### Surfactant transport model.

This study builds upon a lineage of physically grounded models describing surfactant transport in the lungs. At the bifurcation level, the seminal works of Zheng *et al.* [16,17] modeled how a liquid plug propagates, loses volume due to wall coating, and splits between daughter airways across different flow regimes. Upon introduction into the airway tree, the surfactant dose is assumed to form a liquid plug that advances deeper into the branching network during forced inspiration. Two key mechanisms govern the transport process at each bifurcation: (i) airway coating and (ii) plug splitting.

**Airway coating.** As the plug moves along a parent airway of diameter *a*_0_, a trailing film of thickness *h* is deposited on the airway wall. The post-coating volume available for splitting is


$$\begin{array}{c} {\tilde{V}}_{0}=V_{0}(1-\frac{2h}{a_{0}}{)}^{2}\;, \end{array}$$ (2)

where *V*_0_ is the incoming plug volume and *h*/*a*_0_ is the dimensionless film thickness (relative to airway diameter), assumed constant along the parent airway. Following [42], we model *h*/*a*_0_ empirically as a function of the plug capillary number ${Ca}_{p}$:


$$\begin{array}{c} \frac{h}{a_{0}}({Ca}_{p})=0.18(1-\exp[-2\;{Ca}_{p}^{0.523}])\;. \end{array}$$ (3)

This law predicts a rapid increase of coating thickness with ${Ca}_{p}$ followed by saturation, consistent with experimentally observed trailing-film behavior.

**Plug splitting.** At a bifurcation, the post-coating plug volume ${\tilde{V}}_{0}$ is split instantaneously between the two daughter airways through a splitting factor Λ∈[0,1],


$$\begin{array}{c} V_{1}=\Lambda {\tilde{V}}_{0}\;,\;V_{2}=(1-\Lambda ){\tilde{V}}_{0}\;. \end{array}$$ (4)

Λ is obtained from a local pressure balance including capillary pressure jumps, gravity, inertia, and frictional pressure losses. The resulting splitting condition can be written as


$$\begin{array}{c} A\Lambda^{2}+B\Lambda +C=0, \end{array}$$ (5)

where *A*,*B*,*C* depend on local geometry (diameters and branching angles), gravitational orientation, and relevant dimensionless groups (plug capillary, Reynolds, and Bond numbers ${Ca}_{p}$, ${Re}_{p}$, Bo). In the present work, the frictional pressure-loss term uses the nonlinear-plus-linear closure of [17]. Its coefficients are recalibrated using experimental values digitized from the plots reported in [16,17] and assembled into a single continuous closure used across the full range of conditions encountered throughout the airway tree (S1 Text, Sections 2.3 and 2.5).

Plug propagation is modeled as quasi-static and algebraic: at each bifurcation, given the parent plug state (volume and flow rate) and local geometry, the model assembles (*A*,*B*,*C*), solves for Λ, and maps directly to daughter plug volumes and flow rates under a pressure-matching constraint. Intra-branch transients are not modeled.

**Tree-level recursion and multi-aliquot protocols.** This local bifurcation model was extended by Filoche *et al.* [19] and applied recursively throughout the bronchial tree to obtain tractable organ-scale deposition predictions. A key enabler is that, even when long plugs span multiple generations, splitting depends on local pressure differences rather than absolute upstream values, so proximal pressure contributions cancel out in the closure and the same bifurcation-level rules can be applied consistently across generations. The model also supports clinically relevant multi-aliquot protocols, where the total dose is divided into several instillations delivered over successive breaths. Each aliquot forms a distinct plug that propagates independently, and deposition occurs only on previously uncoated walls, consistent with observations that pre-existing thin films have negligible impact on further deposition [13].

Ultimately, the system’s behavior depends on four main components: (i) the airway geometry, defined by tracheal diameter and, in the asymmetric case, a dictionary of local asymmetries (sampled as described in Section Geometry generation and structural parameters and reproducible via fixed seeds); (ii) the number of aliquots, i.e., how the dose is split across breaths; (iii) surfactant properties (viscosity, density, surface tension); and (iv) instillation parameters (total dose, flow rate, body orientation). Body orientation is encoded via a pitch angle γ, defining the component of gravity along the trachea (gravity aids delivery when γ<0), and a roll angle ϕ, defining the lateral gravitational bias. Because left/right is not intrinsic to the abstract airway tree, we define it by convention at the first tracheal bifurcation: $\phi =-90^{\circ }$ corresponds to a left-sided bias and $\phi =+90^{\circ }$ to a right-sided bias. For reference, common clinical postures correspond to the following values:

- standing: $\gamma =-90^{\circ }$, $\phi =0^{\circ }$ ,
- supine (S): $\gamma =0^{\circ }$, $\phi =0^{\circ }$ ,
- prone (P): $\gamma =-180^{\circ }$, $\phi =0^{\circ }$ ,
- left lateral decubitus (LLD): $\gamma =0^{\circ }$, $\phi =-90^{\circ }$ ,
- right lateral decubitus (RLD): $\gamma =0^{\circ }$, $\phi =90^{\circ }$ .

**Recursive application on the full tree.** Surfactant transport is simulated by recursively applying the above local rules at each bifurcation based on local geometry, plug flow rate, and local gravitational orientation. While airway diameters are known and flow rates are propagated downstream, the local pitch and roll angles are recomputed at each bifurcation from cumulative rotations relative to the root, ensuring an anatomically consistent mapping of body posture onto each airway segment (algorithmic details and pseudocode in S1 Text, Section 2.4). This framework lays the foundation for reinforcement learning, where an agent explores instillation and rheological parameters that, under varying anatomical conditions, map to quantifiable deposition patterns through the recursive transport process.

### DRL methodology

#### Single-step deep reinforcement learning for open-loop SRT optimization.

**Fig 4:**
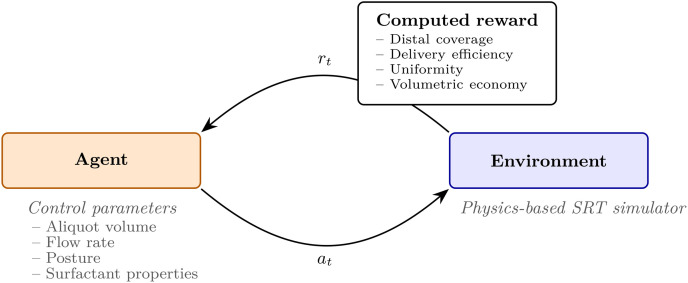
Simplified single-step reinforcement learning (RL) loop for surfactant delivery optimization. The agent selects control parameters (aliquot volume, flow rate, posture, and surfactant properties), which are evaluated by a physics-based environment simulator returning a composite reward that balances distal coverage, delivery efficiency, uniformity, and volumetric economy (see Section Reward selection).

Reinforcement learning (RL) is a framework where an agent learns a policy to maximize cumulative reward through interaction with an environment; in deep reinforcement learning (DRL), this policy is represented by a deep neural network (DNN). Here, we use a single-step DRL formulation tailored to black-box optimization and open-loop control problems, where the optimal action is independent of environment dynamics. In this setting, the agent interacts with the environment only once per episode, which effectively reduces the problem to learning a direct mapping from input to action. This formulation is particularly suitable for SRT instillation, as the optimal sequence of instillation parameters depends on airway geometry and administration protocols, rather than on feedback from intermediate states (Fig 4).

Consequently, rather than learning a policy $\pi^{⋆}$ that maps states to actions to maximize cumulative reward, single-step DRL focuses on learning a fixed transformation $f_{\theta^{⋆}}$ such that $a^{⋆}=f_{\theta^{⋆}}(s_{\text{ini}})$, where *s*_ini_ is the input state, typically fixed. A brief reminder of standard RL/DRL terminology and additional context on the single-step formulation are provided in S1 Text (Sections 3.1 and 3.2).

#### Policy-based optimization.

This work uses an in-house single-step reinforcement learning algorithm, policy-based optimization (PBO), in which the policy is a parametric action-sampling distribution $\pi_{\theta }(a)$ optimized by a policy-gradient update. In our open-loop setting, each episode evaluates one complete protocol (one action) and returns a terminal reward; training is therefore stateless and allows off-policy reuse of past action–reward samples.

At each update, PBO samples actions from a *d*-dimensional multivariate normal distribution $\pi_{\theta }(a)=𝒩(\mu ,\Sigma )$, where *d* is the action-space dimension. Compared to diagonal-covariance policies, using a full covariance matrix allows the sampling distribution to adapt its shape to the local structure of the objective, improving exploration in correlated, higher-dimensional protocol spaces. Actions are sampled in the normalized domain $[-1,1{]}^{d}$ and mapped by the environment to physical protocol parameters.

Policy parameters are updated with Adam optimizer [43] by minimizing


$$\begin{array}{c} L(\theta )={𝔼}_{a\sim \pi_{\theta }}\;\left[\max(\tilde{r},0)\;\log\pi_{\theta }(a)\right]\;, \end{array}$$ (6)

where $\tilde{r}$ is the whitened reward (zero mean, unit variance) used as a one-step advantage estimator. Following the original PBO formulation [36], the $\max(\tilde{r},0)$ factor implements an update that retains only above-average samples; its motivation and practical role are summarized in S1 Text (Section 3.3).

At each training iteration, a batch of actions drawn from the current policy is evaluated in parallel across $n_{env}$ independent simulator instances (here $n_{env}=24$), yielding a batch of action–reward pairs used to form the empirical estimate of the expectation in Eq. (6). This parallel evaluation strategy is used throughout all action-to-protocol optimization experiments.

All algorithmic components follow the published PBO formulation [36] and are reused here without modification. Implementation specifics—including network architectures, training hyperparameters, covariance parameterization, history reuse, and reward normalization/filtering—are provided in S1 Text (Section 3.3), together with pseudocode summarizing the update rule and the end-to-end workflow (S2 Algorithm–S3 Algorithm). For a given airway-tree geometry, the SRT model defines an environment in which each action encodes a complete instillation protocol (aliquot volumes, flow rates, posture schedule, and optionally formulation parameters). Actions sampled from the policy are mapped to interpretable, quantized protocol parameters and evaluated once in the mechanistic simulator. The resulting deposition fields are summarized into clinically motivated metrics and aggregated into a scalar reward that drives a single-step policy update. Iterating this procedure progressively refines protocol designs.

#### Action-to-protocol mapping.

One key aspect of the present research is the evaluation of how varying the number of aliquots *n*_alqt_ affects the delivery of a given total dose volume. To this end, delivery strategies are defined through two complementary types of actions:

- Global actions, which remain constant across all instillations in a delivery sequence. These typically include the total instilled volume and, when applicable, the overall physical properties of the surfactant such as viscosity, density, and surface tension.
- Per-injection actions, which can vary between individual aliquots. These include the fraction of the total dose delivered, the flow rate, and patient posture (e.g., pitch and roll angles). By allowing these parameters to vary, the agent can adapt each instillation to the airway architecture and improve overall performance.

For each global action, the agent outputs a single normalized value $\hat{a}\in [-1,1]$ that is mapped to its corresponding physical range $\left[a_{\min},a_{\max}\right]$ using an affine transformation:


$$\begin{array}{c} a=\frac{1-\hat{a}}{2}\;a_{\min}+\frac{1+\hat{a}}{2}\;a_{\max}\;. \end{array}$$ (7)

For per-injection actions, the agent outputs a low-dimensional representation in the form of $n_{ctrl}$ normalized control points $\{{\hat{a}}_{k}{\}}_{k=1}^{n_{ctrl}}$ per variable, each of which is first mapped to the physical range using Eq (7). These mapped values $\left\{a_{k}\right\}$ are then interpolated over t∈[0,1] with a shape-preserving PCHIP (piecewise-cubic Hermite) spline interpolator to define a smooth profile *a*(*t*). The profile is then sampled at $n_{alqt}$ equally spaced points $t_{j}=(j-1)/(n_{alqt}-1)$ to generate the full per-aliquot sequence $\{a(t_{j}){\}}_{j=1}^{n_{alqt}}$. Finally, both global and per-injection values are quantized to predefined step sizes (adapted to pediatric or adul*t* clinical contexts) before being passed to the simulator for reward evaluation.

This spline-based parametrization addresses a common issue in multi-step control problems, where early actions can disproportionately affect system dynamics and dominate the reward signal. By producing smooth and globally coherent action profiles, it encourages the agent to coordinate all instillations to maximize their collective impact. In practice, setting $n_{ctrl}=6$ provides a satisfactory balance between expressiveness and regularity across all fractionation settings considered here.

Conceptually, the spline-based parametrization allows the number of aliquots *n*_alqt_ to be treated as a decision variable without changing the action vector dimensionality. In practice, however, independent optimizations with fixed aliquot counts proved more efficient. Further implementation details on aliquot-count handling and the training procedure are provided in S1 Text (Section 3.4).

### Reward selection

As in any optimization framework, the notion of an optimal protocol is inherently relative to the objective function used to assess delivery performance, rather than an absolute property of the protocol itself. The next step is thus to define a reward that translates physically grounded delivery objectives into a learning signal for the agent. A key challenge is that no widely accepted metric exists for SRT delivery quality, so “success” must be defined in relation to the specific delivery question being asked, in a way that is both clinically meaningful and computationally tractable. In particular, this requires avoiding metrics that can be dominated by a small subset of poorly dosed terminal branches, which would compress the learning signal, obscure incremental policy improvements, and potentially lead to artificial score collapse in structurally challenging delivery settings. Here, we define success with respect to the following mechanical delivery objective: reaching a sufficiently broad fraction of the distal airway network with an acceptable local deposited amount, while limiting both the influence of localized outlier branches and the use of excessive total instilled volume. This objective is clinically motivated for instilled therapies because terminal regions that receive little or no surfactant remain locally underserved, whereas excessive deposition in a small subset of branches cannot compensate for insufficient delivery elsewhere in the distal network. The resulting reward does not define a universally optimal notion of deposition quality, but specifies the selected delivery objective via a composite formulation built from complementary quantities that remain interpretable across different levels of airway asymmetry.

The reward is centered on a primary branch-level coverage term, which evaluates how many terminal branches receive a dose close to a prescribed local target while avoiding strong local overdosing. As summarized in Table 2, this primary score is complemented by a global efficiency term, which quantifies the fraction of instilled material that reaches the terminal branches; a conditional uniformity term, which measures how evenly the dose is distributed among the branches that are already sufficiently served; and a volume factor, which discourages solutions relying on excessive total instilled volume. Together, these terms define a mechanically grounded reward that distinguishes distal access, branch-level spatial coverage, and refinement of the delivered pattern while keeping objectives on comparable scales. The resulting scores should be interpreted as mechanical delivery proxies within the simulator rather than as direct clinical outcome predictors; further calibration toward clinically informed objectives remains an important direction. Definitions are given below, and parameter values together with related sensitivity tests are reported in S1 Text (Sections 4 and 6).

**Table 2:** Plain-language summary of the composite reward. Formal definitions are provided in Section Reward selection. Parameter values and related sensitivity tests are given in S1 Text (Sections 4 and 6).

Score component	Role	Main knobs
Coverage ($S_{c}$)	Primary tolerance-based branch-level coverage proxy (fraction of well-dosed terminal branches); main driver of the optimization	Local target volume, lower/upper ratio bounds, overdosing decay rate
Efficiency ($S_{e}$)	Global distal delivery term: quantifies how much of the instilled material reaches the terminal tree	—
Cond. uniformity ($S_{u}$)	Auxiliary refinement term: measures relative homogeneity among sufficiently served terminal branches	Selection window for branch ratios
Volume penalty ($S_{v}$)	Global safeguard against excessive total instilled volume relative to the prescribed target	Target total volume, penalty shape
Reward (*r*)	Aggregates complementary objectives; multiplicative structure prevents compensating poor branch-level coverage by high distal delivery or high conditional uniformity alone	—

#### Coverage score.

We first define a branch-level coverage score, intended to quantify how many terminal branches receive a dose close to a prescribed local target. To this end, we first introduce $V_{target}$, the prescribed protocol target volume used as the global reference volume for a given optimization setting. This quantity represents the protocol-level target volume against which delivery performance is evaluated; it is distinct from the total volume actually instilled by the policy, denoted $V_{inst}$. To construct the branch-level coverage score, we then define a distal target volume as a fixed fraction of this prescribed reference,


$$\begin{array}{c} V_{target,d}=f_{d}V_{target}\;, \end{array}$$ (8)

and the associated local distal target volume


$$\begin{array}{c} v_{target,d}=\frac{V_{target,d}}{N}\;, \end{array}$$ (9)

where *N* is the number of terminal branches.

For each terminal branch *i*, let $v_{i}$ denote the delivered volume and define the branch-wise ratio


$$\begin{array}{c} x_{i}=\frac{v_{i}}{v_{target,d}}\;. \end{array}$$ (10)

Each branch is then assigned a local coverage score $s_{c,i}\in [0,1]$ as a function of $x_{i}$, defined by the following piecewise formulation:


$$\begin{array}{c} s_{c,i}=\left\{ \begin{array}{cc} 0, & x_{i}\leq x_{\min}\;,\\ \frac{x_{i}-x_{\min}}{1-x_{\min}}, & x_{\min}<x_{i}\leq 1\;,\\ 1, & 1<x_{i}\leq x_{\max}\;,\\ \exp\;(-k_{over}(x_{i}-x_{\max}))\;, & x_{i}>x_{\max}, \end{array}\right. \end{array}$$ (11)

where $x_{\min}$ defines the lower ratio threshold at which branches begin to receive nonzero credit, $x_{\max}$ is an upper tolerance range above the target, and $k_{over}$ controls the strength of the overdosing penalty.

This formulation deliberately allows a moderate asymmetry around the target: slight underdosing is penalized more strongly than slight overdosing, whereas strong overdosing is again penalized through the exponential decay term. For instilled therapies, a terminal branch receiving substantially less than the target dose is effectively underserved, whereas a modest excess above target may remain acceptable provided it does not result from strong localized overconcentration at the expense of broad distal coverage.

The global coverage score is then defined as the average over all terminal branches, reported on a 0–100 scale:


$$\begin{array}{c} S_{c}=\frac{100}{N}\sum_{i=1}^{N}s_{c,i}\;. \end{array}$$ (12)

This score favors broad distal reach together with branch-level dose matching, while avoiding a trivial gain by strongly overfeeding a few already favored branches. It thus provides an interpretable and clinically motivated component of the reward, and is used as the primary branch-level driver of the optimization.

#### Efficiency score.

Coverage alone does not quantify how much of the instilled material reaches the distal tree. We therefore define a global efficiency score as the fraction of the instilled volume that reaches the terminal branches:


$$\begin{array}{c} S_{e}=\frac{\sum_{i=1}^{N}v_{i}}{V_{inst}}\;. \end{array}$$ (13)

This term corresponds to the standard efficiency metric widely used in the literature, except that it is here rescaled to the interval [0,1] for direct use as a multiplicative modulation factor in the reward. Used in combination, coverage and efficiency prevent overvaluing solutions that reach many terminal branches only weakly, by requiring both broad branch-level coverage and substantial distal delivery.

#### Conditional uniformity score.

Coverage and efficiency do not distinguish whether the terminal branches that are already sufficiently served are themselves evenly matched. To capture this refinement, we define a conditional uniformity score on the subset of branches whose ratio lies within a prescribed admissible interval:


$$\begin{array}{c} 𝒮=\left\{i\;|\;x_{u,min}<x_{i}<x_{u,max}\right\}\;, \end{array}$$ (14)

where $x_{u,min}$ and $x_{u,max}$ define the lower and upper ratio thresholds used to select the terminal branches included in the conditional uniformity calculation.

If fewer than two branches belong to 𝒮, the score is set to zero. Otherwise, we define


$$\begin{array}{c} S_{u}=\frac{1}{1+{CV}_{𝒮}}\;, \end{array}$$ (15)

where ${CV}_{𝒮}$ is the coefficient of variation of the branch-wise volume ratio distribution $\{x_{i}{\}}_{i\in 𝒮}$.

This term is conceptually related to the standard homogeneity metric widely used in the literature, but differs in two important respects. First, high values of $S_{u}$ do not imply globally uniform delivery across the full terminal tree, because it is evaluated only on the subset of terminal branches lying within a prescribed branch-wise ratio range, rather than on the full terminal population; this prevents severely underdosed branches from dominating a quantity whose purpose is to describe the relative uniformity of the branches that are already close to the intended dosing range. Second, the score is mapped to the interval (0,1], unlike unbounded deviation-based metrics, and is thus directly usable as an additional multiplicative refinement term rather than as a primary performance driver.

#### Volume penalty.

To prevent the optimizer from exploiting excessive total instilled volume, we introduce a global multiplicative volume factor. We define


$$\begin{array}{c} S_{v}=\left\{ \begin{array}{cc} 1, & X\leq 1\;,\\ \max(0,2-X{)}^{K_{over}}, & X>1\;, \end{array}\right. \end{array}$$ (16)

where


$$\begin{array}{c} X=\frac{V_{inst}}{V_{target}}\;, \end{array}$$ (17)

denotes the ratio between the total instilled volume and the user-defined prescribed target volume for instillation, and $K_{over}$ controls the strength of the global overvolume penalty; $k_{over}$ denotes the corresponding local overdosing penalty in the branch-level coverage score.

This factor leaves solutions at or below the prescribed target volume unchanged, while progressively downweighting solutions that rely on excess instilled volume. The penalty remains bounded and preserves a useful learning signal for moderate excess, while suppressing strategies that achieve high distal scores only by substantially exceeding the intended total volume.

#### Reward formulation.

The final reward combines the previous terms multiplicatively:


$$\begin{array}{c} r=S_{c}\;S_{e}\;S_{u}\;S_{v}\;. \end{array}$$ (18)

This structure reflects the intended hierarchy of objectives. The coverage score $S_{c}$ acts as the primary branch-level driver; the efficiency term $S_{e}$ prevents solutions with broad but very weak distal delivery from being overvalued; the conditional uniformity term $S_{u}$ rewards smoother branch-level allocation among sufficiently served branches; and the volume factor $S_{v}$ suppresses strategies that rely on excessive total volume. The multiplicative form also prevents a very poor value in one component from being fully compensated by excelling in another. Since $S_{e}$, $S_{u}$, and $S_{v}$ are bounded modulation factors in [0,1], the resulting reward naturally follows the 0–100 scale of the coverage score.

**Fig 5:**
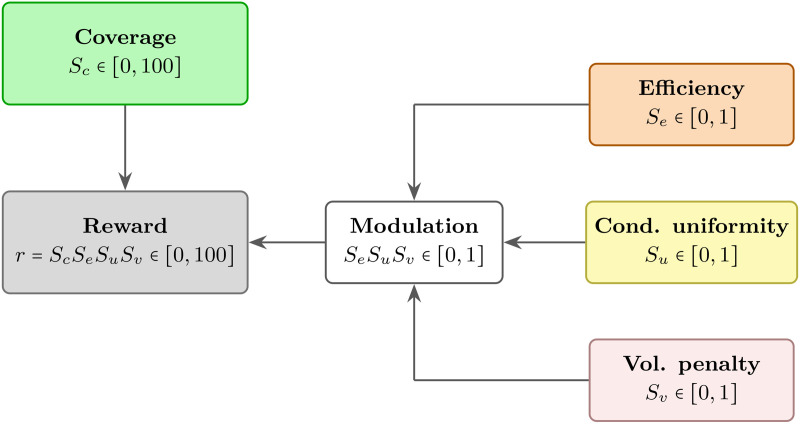
Reward computation graph. Arrows denote data flow. Coverage is the primary reward driver and sets the overall reward scale. Efficiency, conditional uniformity, and volume penalty enter as bounded multiplicative factors. Formal definitions are provided in Section Reward selection. Parameter values and related sensitivity tests are given in S1 Text (Sections 4 and 6).

A synthetic computation graph summarizing this reward pipeline is provided in Fig 5. For clarity, the numerical parameters and thresholds defining the reward and its constituent scores are summarized in S1 Text (Section 4). The reward metrics defined above serve as mechanical delivery proxies and are not intended as clinical outcome surrogates. Nevertheless, the formulation emphasizes physically grounded objectives aligned with clinically motivated goals for instilled therapies—broad distal reach, sufficiently widespread deposited mass, and robustness to localized maldistribution—as heterogeneous surfactant spreading has been linked to impaired gas exchange and regional atelectasis [44]. The framework is methodologically portable, and recalibration against imaging-based or *in vitro* deposition readouts is an important direction. However, any translational use would require adapting these proxies and restricting the protocol space to bedside-feasible actions, as well as validation against experimental and imaging readouts; we thus view the present formulation as a controlled feasibility benchmark rather than a clinically prescriptive tool.

### Numerical experiments

#### Optimization scenarios.

The objective of this study is to identify effective surfactant delivery strategies by assessing how increased experimental flexibility affects achievable delivery performance. To this end, we define four optimization scenarios of increasing dimensionality, whose respective action spaces and vector dimensions are summarized in Table 3. The first three scenarios form a staged mechanical-control hierarchy, progressing from global protocol parameters to per-aliquot dose scheduling and posture control. The fourth scenario then adds surfactant rheology as an additional formulation-level lever beyond this purely mechanical hierarchy.

- *Baseline:* The agent optimizes only global protocol parameters, namely the total instilled volume (split evenly between aliquots) and the driving flow rate (identical for all aliquots). Patient posture alternates between left and right lateral decubitus to mimic standard clinical practice. The agent output thus consists of *d* = 2 variables. Although this low dimensionality would allow a simpler parametric study, DRL is maintained to ensure methodological consistency across scenarios.
- *Per-dose:* The agent additionally optimizes per-injection parameters, specifically the fraction of total volume and the flow rate assigned to each aliquot, while the total dose volume remains a global variable. Patient posture still alternates between left and right lateral decubitus following standard clinical protocols. The agent output consists of *d* = 2 *n*_ctrl_+1 variables. After quantization, the volume fractions are rescaled to satisfy a unit-sum constraint, ensuring that the total volume is exactly allocated across all aliquots.
- *Per-dose + Posture:* Patient posture is introduced as an additional per-injection variable (roll and pitch angles), allowing the agent to tailor each instillation to airway geometry. The total dose volume remains global, giving *d* = 4 *n*_ctrl_+1 variables.
- *Per-dose + Posture + Rheology:* Building on the preceding mechanical optimizations, this final scenario introduces surfactant formulation as an additional control lever. Viscosity, density, and surface tension are optimized jointly with the per-aliquot dosing profiles and posture schedule, yielding the full action space with *d* = 4 *n*_ctrl_+4. This final step therefore constitutes a second ablation layer beyond purely mechanical control, isolating the added value of formulation tuning once dose scheduling and posture have already been optimized.

**Table 3:** Summary of optimization scenarios. Each scenario increases the degrees of freedom available to the agent, from global-only parameters to full per-dose and rheology control. LLD and RLD refer to left and right lateral decubitus orientations used in clinical protocols. The last column indicates the dimensionality of the corresponding action vector, where *n*_ctrl_ denotes the number of spline control points parameterizing each per-injection variable.

Scenario	Actions		Optimization		Action space dimension
	Global	Per dose	Posture	Surfactant	
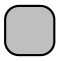 Baseline	Instilled vol.	—	×	×	2
	Flow rate		LLD/RLD		
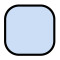 Per-dose	Instilled vol.	Vol. fraction	×	×	2 *n*_ctrl_+1
		Flow rate	LLD/RLD		
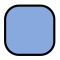 Posture	Instilled vol.	Vol. fraction	✔	×	4 *n*_ctrl_+1
		Flow rate			
		Posture			
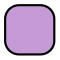 Rheology	Instilled vol.	Vol. fraction	✔	✔	4 *n*_ctrl_+4
	Surfactant	Flow rate			
		Posture			

For compact display in subsequent tables and figures, the four scenarios are abbreviated as Baseline, Per-dose, Posture, and Rheology, where each label denotes the incremental addition of the corresponding control lever relative to the preceding scenario. This staged set of optimization scenarios acts as an ablation over control-space dimensionality, as we progressively add clinically motivated and mechanically interpretable levers (dose fractionation, per-aliquot dosing/flow, posture, and rheology), enabling lever-by-lever quantification of performance changes.

Each strategy is evaluated for both pediatric and adult airway models across symmetric, mildly asymmetric, and highly asymmetric anatomical conditions. For each asymmetry level, we use five stochastic airway-tree realizations (Table 1) to probe trend-level robustness and anatomy-driven variability rather than cohort-level generalization. Accordingly, we emphasize effect sizes and sign-consistency of scenario-to-scenario gains across realizations, rather than formal statistical significance testing.

Under the single-step RL formulation used across all scenarios, the action vector encodes the entire multi-aliquot sequence (volume fractions, flow-rate profiles, posture schedule—and global rheology when applicable), and the SRT model returns a single terminal reward after the complete administration. There are no inter-aliquot observations; hence the policy maps airway morphology and scenario to a full control trajectory in one shot.

#### Instillation protocol variables.

To ensure that these optimization problems remain physically meaningful and comparable across pediatric and adult settings, protocol parameters (instilled volume, volume fraction, flow rate, and patient posture) are constrained within order-of-magnitude, physiologically plausible ranges, summarized in Table 4. Throughout the manuscript, quoted protocol volumes refer to the total instilled volume $V_{inst}$, unless stated otherwise. For each optimization setting, the target volume $V_{target}$ is prescribed externally and used as a reference quantity in reward construction. The agent then selects $V_{inst}$, together with the other protocol parameters, and the resulting protocol is evaluated relative to $V_{target}$. Quantization step sizes used to map normalized agent outputs to physical units are set to 0.02 mL (pediatric) and 1 mL (adult) for volume, 0.02 mL/s and 2 mL/s for flow rate, and 15 ° for gravitational angles across all scenarios. Volume and flow quantization are chosen to preserve sufficient reward sensitivity and avoid flattening the optimization landscape, while angular discretization restricts posture control to a finite, interpretable set. This can be viewed as a first step toward feasibility; additional feasibility constraints (e.g., admissible posture subsets or limited posture changes across aliquots) can be incorporated through constrained action spaces in future work.

**Table 4:** Parameter ranges used during protocol optimization for the total instilled volume, aliquot dose fraction, flow rate, and posture, for a 1 kg neonate and a 70 kg adult. Normalized values are expressed per kilogram of body weight.

Group	Instilled vol. [mL]	Vol. fraction	Flow rate [mL/s]	Pitch angle [°]	Roll angle [°]
Pediatric	0.2–8	0.1–1	1–7	-180–0	-90–90
Adult	14–560		70–490		
Normalized per kg	0.2–8	—	1–7	—	—

Despite substantial anatomical and physiological differences between premature infants and ventilated adults, we use the same body-weight–normalized ranges as a cross-scale parameterization to enable controlled comparisons within the simulator (volumes: 0.2–8 mL/kg; flow rates: 1–7 mL/kg/s). In the numerical experiments reported below, the prescribed target volumes explored within these ranges are 1–4 mL in the pediatric model and 70–280 mL in the adult model, corresponding in both cases to 1–4 mL/kg. This normalization is not intended to enforce mechanical equivalence across ages or clinical contexts (e.g., differences in compliance, resistance, ventilatory settings, or disease mechanics). Rather, it provides a practical and interpretable way to span plausible operating regimes and to ask a first-pass feasibility question within the present model: whether delivery strategies can in principle achieve high deposition scores at adult scale without resorting to extreme dosing. Additional justification of the explored volume and flow-rate ranges, and of the interpretation of flow rate as a proxy for ventilatory airflow, is provided in S1 Text (Section 5). Establishing clinically calibrated mappings between ventilator settings, airway mechanics, and effective instillation ranges is beyond the scope of this study and is left for future work.

#### Surfactant physical properties.

In the first three scenarios (Baseline, Per-dose, Per-dose + Posture), the rheological properties of the surfactant are held fixed to representative values reported for existing formulations. These formulations are typically biologically derived rather than synthetic, and are designed to mimic key functional properties of natural pulmonary surfactant. Specifically, viscosity is set to 30 cP, based on measurements for Survanta, Infasurf, and Curosurf [45,46]; density is assumed to be aqueous carrier-like (1 g/cm^3^); and surface tension is fixed at 30 dyn/cm [47,48]. In our plug-transport model, σ should be interpreted as an effective air–liquid interfacial parameter governing capillary-pressure jumps and coating during airway propagation, rather than as a full descriptor of dynamic minimum alveolar surface tension.

In the last scenario (Per-dose + Posture + Rheology), the agent is granted control over surfactant rheology to explore formulation–protocol trade-offs beyond currently marketed preparations. We thus allow an intentionally exploratory but physically plausible design space (Table 5): surface tension is bounded by σ≤40 dyn/cm, below the air–carrier value (≈70 dyn/cm, corresponding to air–water/air–saline) to ensure surface-active behavior; density ranges from 0.7 to 1.3 g/cm^3^; and viscosity spans 10–100 cP, covering low-resistance to more viscous solutions that remain practically instillable. These ranges overlap those previously explored in surfactant-transport modeling [19,49] (e.g., μ down to a few cP and σ in the few–tens of dyn/cm regime, including a few dyn/cm), and encompass higher effective interfacial values reported under experimental conditions [50]. Parameter quantization steps are set to 1 for viscosity and surface tension, and 0.01 for density.

**Table 5:** Parameter ranges used during surfactant rheology optimization.

Group	Viscosity [cP]	Density [g/cm^3^]	Surface tension [dyn/cm]
Pediatric	10–100	0.7–1.3	5–40
Adult			

These numerical experiments define the parameter space used for optimization. Performance is then evaluated through the composite reward introduced in Section Reward selection, which combines complementary metrics of branch-level coverage, distal delivery efficiency, conditional uniformity, and global volume penalization. The corresponding results are reported in Section Results and Discussion.

## Results and discussion

We examine here how successive control levers—dose allocation, posture, and rheology—shape delivery performance across scales and airway geometries. Unless otherwise specified, all comparisons are interpreted primarily through the coverage score $S_{c}$, as it serves as the main branch-level performance endpoint and sets the overall reward scale. The efficiency, conditional uniformity, and volume penalty terms are used secondarily to explain finer reward differences when needed. This metric remains informative in large and asymmetric trees, where stricter deviation-based summaries can be dominated by a small number of extreme outliers and obscure broader improvements in branch-level coverage (see Section Limitations of homogeneity-based delivery metrics and the role of asymmetry).

As a first step, we examine representative convergence behavior through pediatric and adult training trajectories shown in S1 Text (Section 7.1). These trajectories report exponential moving averages (EMA) of the reward and its components, computed over a 10-episode window. The corresponding terminal values used below are computed as the mean of the last ten EMA values. The selected runs show smooth stabilization within the training horizon, with small residual fluctuations over the final training window (the intra-run standard deviation over the final window remains systematically below 1–2 %), supporting the stability of the optimized delivery outcomes reported below. These examples are consistent with the broader training behavior observed across the tested settings; additional runs and scenario-specific convergence summaries are available in the associated Zenodo deposit [51], which documents the full code and data associated with this study.

### Protocol optimization

We now examine how progressively enriching the control space—first through per-aliquot dose/flow optimization, then through posture adaptation—shapes achievable delivery performance across airway geometries and age groups. This staged design supports a controlled ablation over delivery-relevant protocol levers. Accordingly, we report (i) incremental scenario-to-scenario gains within each age group and geometry, and (ii) the remaining adult–pediatric gap at matched scenario complexity. We first summarize these global shifts, then analyze branch-level deposition maps and error statistics to disentangle the respective roles of dosing and posture. Unless otherwise specified, reported target-volume conditions in the Results refer to the externally prescribed reference volume $V_{target}$ that defines the optimization setting. The optimized protocol may instill a different total volume $V_{inst}$, selected by the agent and penalized relative to $V_{target}$ through the reward. The resulting evolution of $V_{inst}$ as a function of $V_{target}$ is therefore itself an informative outcome of the optimization.

Because training is stochastic and the search landscape quickly becomes highly non-convex, independent runs can converge to distinct near-equivalent optima. To separate optimization variability from anatomical variability, these two sources of dispersion are analyzed at different levels. Run-to-run variability is quantified in S1 Text through independent training-seed comparisons of final scores and learned-policy alignment, including reduced action-space comparisons that identify which control components carry most of the policy variability. These analyses show weak dispersion of the attained physical scores across runs, even when several near-equivalent policy realizations coexist. This behavior is not unexpected: the reward is outcome-oriented, i.e., it rewards delivery quality rather than a unique control realization, so different protocols may legitimately achieve very similar final scores. In the following, we therefore report, for each setting, the best-performing policy among three independent runs as a pragmatic upper bound under a fixed compute budget.

Anatomical variability is assessed separately in the main-text asymmetric comparisons by repeating the optimized analyses across five independent airway-tree realizations for each asymmetry level. For each realization, the best-performing policy is selected independently among the three training runs before summarizing performance across airway realizations.

Quantitative performance metrics are summarized in the scenario-specific tables below, and selected branch-level score maps are visualized in the corresponding figures. In the main text, we focus on symmetric and highly asymmetric trees; mild asymmetry is reported in S1 Text as an intermediate check. For asymmetric models, main-text values are summarized as medians with interquartile ranges across five airway realizations (seeds 1–5; Table 1), with the interquartile range providing a compact measure of anatomy-driven variability in attained performance. Their generally limited magnitude in the main comparisons indicates that the reported performance trends are consistently recovered across the tested airway realizations, even though the specific protocol realizations need not be identical.

We do not exhaustively list optimal schedules, because multiple distinct schedules can perform similarly and because small schedule-level differences can arise from coupled interactions among dose partitioning, flow, posture, and airway-specific gravitational routing, making direct schedule-to-schedule comparisons difficult to interpret. Instead, we focus on selected spatial patterns and lever-specific performance shifts that most directly support the ablation logic of the study and help identify the physical mechanisms underlying high-performing strategies. The main trends are summarized at the end of this section to show how the reward structure promotes interpretable, physically consistent control patterns while providing insight into patient- and geometry-specific optimization. All figures in this section use the same color conventions for optimization scenarios as in Table 3 to facilitate comparison across experiments.

**Fig 6:**
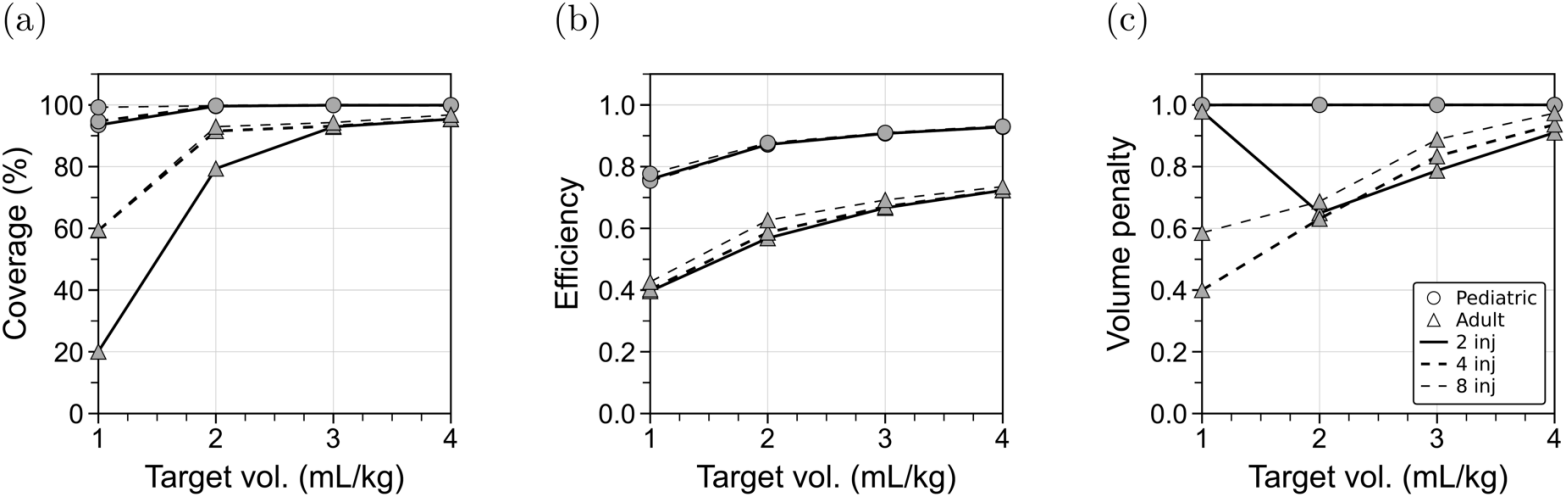
Baseline protocol in the reference symmetric airway models: effect of prescribed target volume $V_{target}$, stratified by aliquot number, on (a) the coverage score $S_{c}$, (b) the efficiency term $S_{e}$, and (c) the volume penalty term $S_{v}$. Circles and triangles denote pediatric and adult groups, respectively; line styles denote aliquot number.

**Fig 7:**
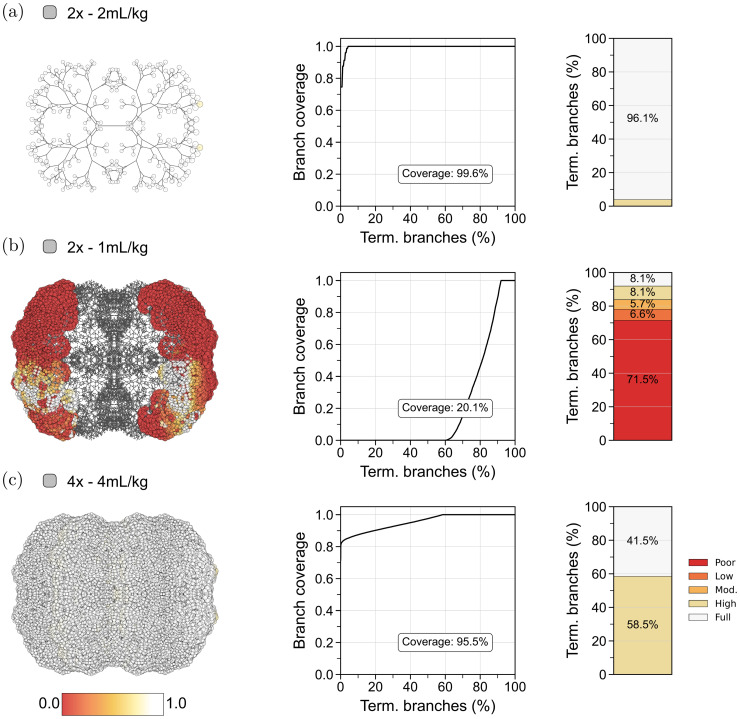
Representative branch-level score patterns under the optimized baseline protocol in the reference symmetric airway models. **(a)** Pediatric, 2 aliquots, target volume $V_{target}=2\;mL/kg$ (2 mL). Left: branch-level coverage map, with color encoding local coverage scores (lighter colors indicate higher scores) and circle size indicating the local relative error relative to the target local volume. Middle: sorted local coverage scores across terminal branches. Right: corresponding fractions of terminal branches in five local-score classes. **(b,c)** Same as (a) for (b) adult, 2 aliquots, 1mL/kg (70 mL). **(c)** Same as (a) for the adult model, 4 aliquots, 4mL/kg (280 mL).

**Baseline performance in symmetric models.** Quantitative performance metrics are summarized in Table 6, with the evolution of the key reward components with target volume shown in Fig 6 and selected branch-level score maps illustrated in Fig 7. Additional metrics reported in S1 Text (Section 7.2.1) further refine this picture.

**Table 6:** Coverage scores achieved under the optimized baseline protocol in the reference symmetric airway models. The body-weight–normalized target-volume range of 1–4 mL/kg corresponds to 1–4 mL in the pediatric setting (1 kg neonate) and 70–280 mL in the adult setting (70 kg adult).

Scenario	Group	Target vol. [mL/kg]	No. aliquots		
			2×	4×	8×
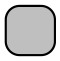 Baseline	Pediatric	1	93.5	94.7	99.2
		2	99.6	99.7	99.7
		3	99.9	99.9	99.9
		4	99.9	99.9	99.9
	Adult	1	20.1	59.3	59.5
		2	79.4	91.5	93.0
		3	92.9	93.1	94.3
		4	95.4	95.5	96.8

A strong scale-dependent contrast already appears in the symmetric reference geometry. In the pediatric model, the delivery problem is favorable almost immediately: as shown in Fig 6(a), coverage is already high at a target volume of 1mL/kg and rapidly enters a near-ceiling regime, increasing from 93.5 to 99.6−99.9 between 1 and 2−4mL/kg, with only minor differences between 2, 4, and 8 aliquots. In the adult model, by contrast, low prescribed target volumes remain strongly limiting. At 1mL/kg (70mL), coverage is only 20.1 with 2 aliquots and rises only to about 59 with 4–8 aliquots, showing that fractionation alone cannot compensate for insufficient total dose. Once the prescribed volume reaches 2mL/kg (140mL), however, coverage rises sharply to 79.4, 91.5, and 93.0 for 2, 4, and 8 aliquots, respectively, before approaching a high-performance regime at 3−4mL/kg. This contrast is also reflected in the adult–pediatric gap: it is maximal at 1mL/kg, reaching about 73 points for 2 aliquots and still ~35−40 points for 4–8 aliquots, but quickly collapses once the adult regime becomes mechanically accessible, falling to about 20 points at 2mL/kg for 2 aliquots and to only 7−8 points for 4–8 aliquots, before reaching only a few points at 3−4mL/kg. Thus, prescribed volume is the dominant first-order lever, especially in adults, where a sufficiently large target volume is required to make broad distal coverage mechanically accessible.

The efficiency curves in Fig 6(b) clarify the origin of this contrast. In the pediatric model, efficiency is already relatively high at 1mL/kg (≈0.75−0.78) and then increases smoothly toward ≈0.93. In the adult model, it starts much lower (≈0.40−0.43) and rises only gradually to ≈0.72−0.74 at 4mL/kg. The main low-volume adult limitation is therefore not poor balancing among already reached branches, but restricted distal access itself. This is consistent with the stronger need for overinstillation in adults and helps explain why the low-volume regime is much more constrained at adult than at pediatric scale.

Within this volume-limited adult regime, fractionation remains beneficial but acts mainly as a secondary refinement lever. Its direct effect at fixed volume is most visible at low and intermediate doses, whereas gains become marginal near saturation. More importantly, fractionation also improves the apparent volumetric efficiency of the protocol, in the sense that comparable coverage can be achieved at lower prescribed volume: in the adult symmetric model, 4–8 aliquots at 2mL/kg already achieve coverage comparable to or better than 2 aliquots at 3mL/kg (91.5−93.0 versus 92.9). Thus, finer partitioning does not simply raise the attainable score; once the prescribed volume enters an effective range for distal access, it shifts the onset of the high-coverage regime toward lower volumes. This effect remains secondary to the volume threshold itself, but is practically meaningful because it reduces the dose required to reach a given coverage level.

The volume penalty term, which downweights solutions with $V_{inst}>V_{target}$, is mainly useful to interpret the residual trade-offs in the low-volume adult regime; see Fig 6(c). In pediatric symmetric cases, it remains essentially equal to one, consistent with the fact that high coverage is achieved without substantial overinstillation. In adults, by contrast, it is markedly reduced at 1−2mL/kg, especially for fractionated protocols, showing that some of the best low-volume solutions are obtained by accepting moderate excess volume in exchange for a much broader distal reach. Accordingly, the occasional low-volume outliers are not numerical anomalies but signatures of a genuine mechanical trade-off: some runs preserve the volume budget better but remain poorly covering, whereas others accept a stronger penalty and recover substantially higher coverage.

Taken together, these symmetric results establish a simple baseline hierarchy: prescribed volume is the dominant first-order driver of performance, fractionation provides a secondary but meaningful improvement in volumetric efficiency, and the adult scale remains substantially more access-limited than the pediatric one even under otherwise favorable geometry. Fig 7 illustrates this baseline narrative through three representative cases: the pediatric model with 2 aliquots and 2mL, the adult model with 2 aliquots and 70mL, and the adult model with 4 aliquots and 280mL. The first case is already close to trivial, with nearly all branches well covered. The second is clearly underdosed, with a large fraction of poorly served or unreached branches and a strong spatially structured deficit. The third reopens a high-coverage regime, marked by a substantial contraction of the low-score tail and the disappearance of most poorly covered branches.

Although the present symmetric setting is intentionally idealized, the mechanically accessible volume range observed here remains compatible with independent order-of-magnitude estimates. As shown in Table 7, the corresponding alveolar film thicknesses remain within physiologically plausible ranges, typically tens of nanometers to a few micrometers, consistent with effective surface tension reduction and normal respiratory function [52]. These reported ranges are intended only as an a posteriori check within this regime, estimated under a uniform-coverage assumption from total alveolar surface area [53]. In the adult case, the onset of broad distal accessibility is also consistent with prior modeling work indicating that volumes on the order of 150–200 mL are required to enter a mechanically accessible regime, even if coverage remains incomplete [19]. More broadly, these adult-scale volumes remain within the order of magnitude explored in prior experimental and clinical feasibility studies of surfactant instillation in adults [4,11,54,55]. This agreement should not be interpreted as direct translational validation, but rather as support for the plausibility of the volume range over which the performance transition is observed.

**Table 7:** Order-of-magnitude estimate of alveolar film thickness in the mechanically feasible delivery regime identified by the optimization, assuming uniform coverage of the alveolar surface. The distal delivered volume is estimated here as approximately 90% of the instilled volume in the pediatric setting and 75% in the adult setting, consistent with typical optimized efficiencies in this regime. These values are intended only as illustrative post hoc ranges rather than unique protocol values.

Group	Instilled vol. [ mL]	Distal delivered vol. [ mL]	Alveolar surface [ m^2^]	Film thickness [μm]
Pediatric	1–2	0.9–1.8	3	0.3–0.6
Adult	200–280	150–210	80	1.9–2.6

**Fig 8:**
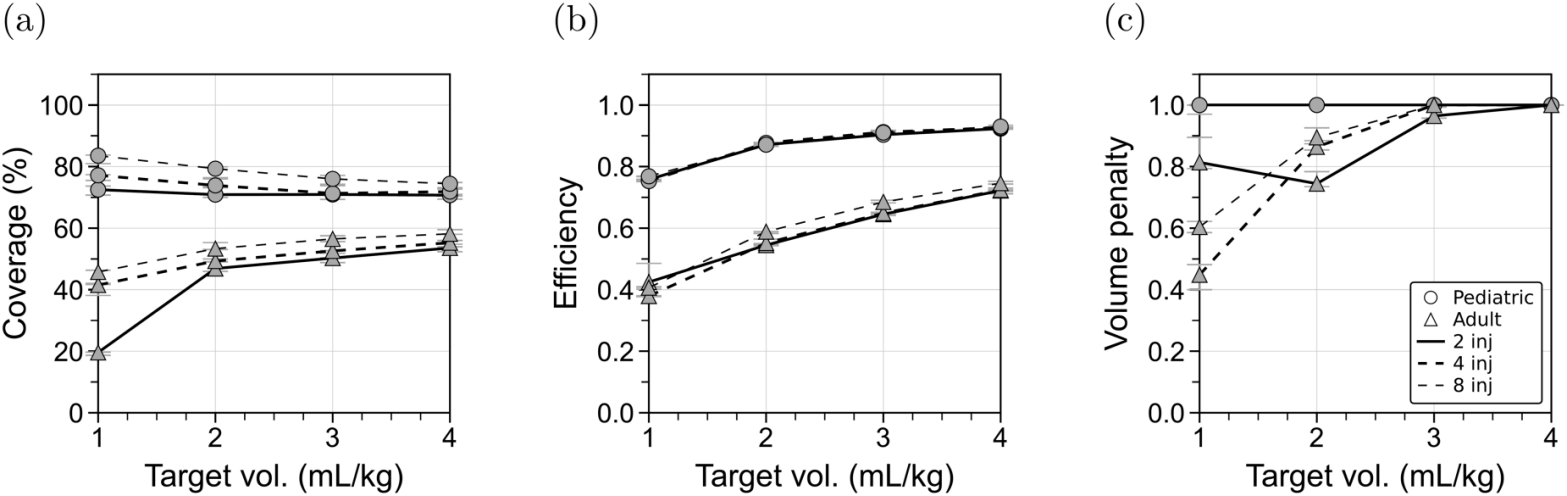
Baseline protocol in the highly asymmetric airway models: effect of prescribed target volume $V_{target}$, stratified by aliquot number, on (a) the coverage score $S_{c}$, (b) the efficiency term $S_{e}$, and (c) the volume penalty term $S_{v}$. Circles and triangles denote pediatric and adult groups, respectively; line styles denote aliquot number. Marker positions indicate medians, and error bars indicate the interquartile range (Q1–Q3) across the five airway realizations (seeds 1–5; see Table 1).

**Fig 9:**
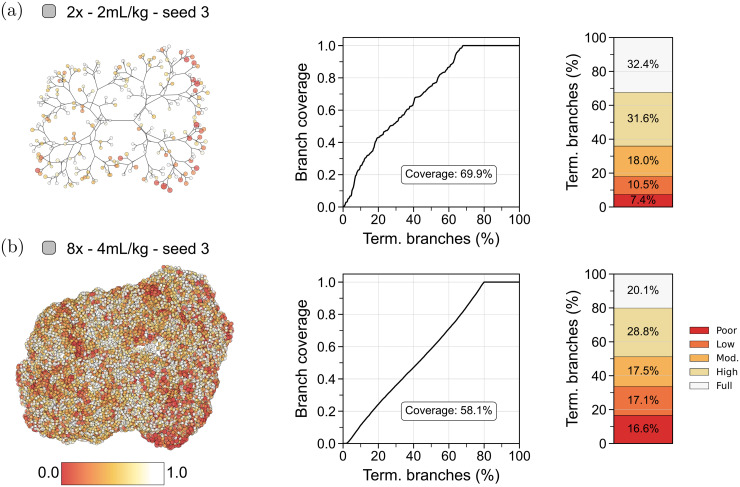
Representative branch-level score patterns under the optimized baseline protocol in the highly asymmetric airway models. **(a)** Pediatric, 2 aliquots, target volume $V_{target}=2\;mL/kg$. **(b)** Adult, 8 aliquots, target volume $V_{target}=4\;mL/kg$. Same conventions as in Fig 7.

**Baseline performance in highly asymmetric models.** We next assess how the same baseline protocol behaves under strong geometric asymmetry, using the five stochastic airway-tree realizations described in Section Geometry generation and structural parameters for each condition. Performance metrics are summarized in Table 8 as median [IQR] across these five realizations, with the evolution of the key reward components with target volume shown in Fig 8 and selected branch-level score maps illustrated in Fig 9. Additional metrics for asymmetric geometries are reported in S1 Text (Sections 7.2.2 and 7.2.3), including mildly asymmetric baseline results and supplementary high-asymmetry metrics.

**Table 8:** Coverage scores achieved under the optimized baseline protocol in the highly asymmetric airway models. Values report the median [IQR] across the five airway realizations (seeds 1–5; see Table 1). The body-weight–normalized target-volume range of 1–4 mL/kg corresponds to 1–4 mL in the pediatric setting (1 kg neonate) and 70–280 mL in the adult setting (70 kg adult).

Scenario	Group	Target vol. [mL/kg]	No. aliquots		
			2×	4×	8×
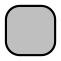 Baseline	Pediatric	1	72.5 [3.0]	77.2 [1.9]	83.5 [2.8]
		2	70.9 [3.5]	73.9 [2.9]	79.3 [3.6]
		3	70.9 [2.1]	71.3 [2.8]	76.0 [2.9]
		4	70.7 [1.5]	71.9 [2.3]	74.4 [1.7]
	Adult	1	19.6 [1.0]	41.5 [3.6]	45.8 [4.2]
		2	46.9 [3.0]	49.3 [0.9]	53.3 [2.2]
		3	50.3 [1.6]	52.6 [0.9]	56.5 [1.9]
		4	53.6 [1.6]	55.3 [1.2]	58.1 [1.5]

Relative to the symmetric reference geometry, strong asymmetry induces a marked and scale-dependent degradation of coverage performance, as shown in Fig 8(a). The pediatric model remains comparatively resilient: median coverage stays in the 70−75 range with 2 aliquots and in the 74−84 range with 8 aliquots across the explored target volumes, indicating that broad distal coverage remains mechanically accessible despite the asymmetric branching pattern. By contrast, the adult model becomes strongly constrained. At a target volume of 1mL/kg, median coverage is only 19.6 for 2 aliquots, and even with stronger fractionation reaches only 45.8 for 8 aliquots. Across realizations, the corresponding IQRs remain small to moderate, indicating that these trends are robust at the trend level despite residual anatomy-dependent variability.

Increasing prescribed volume still improves performance, but much less dramatically than in the symmetric adult case: at 4mL/kg, median coverage reaches only 53.6, 55.3, and 58.1 for 2, 4, and 8 aliquots, respectively. Thus, in the highly asymmetric adult regime, neither volume nor fractionation is sufficient to recover the high-coverage plateau seen in the symmetric geometry. Additional exploratory tests at stronger fractionation or higher volume did not materially alter this picture. In the highly asymmetric adult model, increasing fractionation from 8 to 12 aliquots at 4mL/kg raises median coverage only from 58.1 to 61.3, while increasing target volume from 4 to 5mL/kg at 8 aliquots raises it only from 58.1 to 59.5. These extensions therefore provide only marginal gains relative to the main trend; see also Section Limitations of homogeneity-based delivery metrics and the role of asymmetry, where we discuss how global deviation metrics can obscure such trends under strong asymmetry.

This degradation is also reflected in the adult–pediatric gap, which remains large throughout the explored range instead of collapsing at higher volume as in the symmetric case. For 2 aliquots, the gap is about 53 points at 1mL/kg and still remains around 17 points at 4mL/kg. For 8 aliquots, it decreases from about 38 to 16 points over the same range, but never approaches the near-convergence observed in symmetric models. Strong asymmetry therefore changes not only the absolute performance level, but also the overall structure of the delivery problem: the adult regime remains persistently access-limited even when target volume is increased.

By contrast, the efficiency curves in Fig 8(b) remain qualitatively close to those observed in symmetric models. Pediatric values remain systematically higher than adult ones, and both scales still show a monotonic increase with prescribed volume and a modest benefit from fractionation. Thus, strong asymmetry does not primarily alter the global distal-access trends captured by $S_{e}$; rather, its main effect is to degrade how this distal delivery translates into branch-level coverage. A similar qualitative behavior is observed for the volume penalty term in Fig 8(c), as well as for the conditional uniformity term (S1 Text, Section 7.2.3).

This picture is illustrated in Fig 9, which shows two representative highly asymmetric cases: the pediatric model with 2 aliquots and 2mL, and the adult model with 8 aliquots and 280mL. In the pediatric case, coverage remains substantial but becomes visibly more heterogeneous than in the symmetric reference geometry, with a broader branch-level score distribution and a non-negligible low-score tail. In the adult case, even under the most favorable baseline setting explored here, coverage remains incomplete, with widespread poorly served regions and a large fraction of terminal branches that remain only moderately covered. These maps therefore make explicit the main conclusion of this section: strong asymmetry primarily degrades the ability of the baseline protocol to achieve satisfactory branch-level coverage, an effect that is especially pronounced in the adult model.

**Fig 10:**
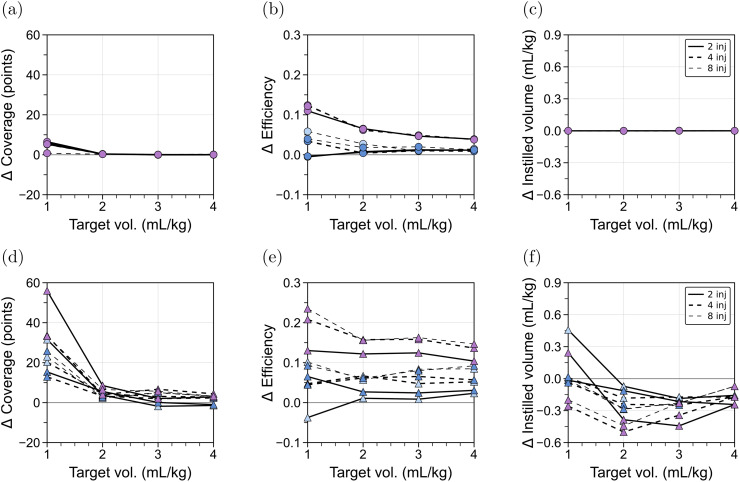
Optimized protocols in the reference symmetric airway models: effect of prescribed target volume $V_{target}$, stratified by aliquot number and scenario complexity, on (a,d) the coverage score $S_{c}$, (b,e) the efficiency term $S_{e}$, and (c,f) the total instilled volume, shown as differences relative to their baseline values. Panels **(a)**–**(c)** correspond to the pediatric group and panels **(d)**–**(f)** to the adult group. Improvement therefore corresponds to positive values in (a,b,d,e) and to negative values in **(c,f)**. Scenario colors follow Table 3: Baseline (gray), Per-dose (light blue), Per-dose + Posture (blue), and Per-dose + Posture + Rheology (purple).

**Effect of scenario complexity in symmetric models.** We next quantify how progressive enrichment of the control space modifies performance in the symmetric reference geometry, starting from the optimized baseline protocol and then adding, in a staged manner, per-dose adjustment, posture scheduling, and rheology control. To isolate the contribution of these additional levers, Fig 10 reports all results as differences relative to the baseline for the coverage score, the efficiency term, and the total instilled volume; selected absolute values are reported in Table 9, and additional metrics are provided in S1 Text (Section 7.3.1).

**Table 9:** Optimal coverage scores achieved in the reference symmetric airway models using a staged scenario design in which each scenario adds one additional control lever (Baseline → Per-dose → Per-dose + Posture → Per-dose + Posture + Rheology). For clarity, only the pediatric 1mL/kg condition is reported, since higher pediatric volumes are already near ceiling in all scenarios. The body-weight–normalized target-volume range of 1–4 mL/kg corresponds to 1–4 mL in the pediatric setting (1 kg neonate) and 70–280 mL in the adult setting (70 kg adult).

Scenario	Group	Target vol. [mL/kg]	No. aliquots		
			2×	4×	8×
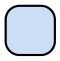 Per-dose	Pediatric	1	98.7	99.9	100.0
	Adult	1	51.7	79.6	82.5
		2	83.2	95.8	95.3
		3	91.1	96.2	99.1
		4	94.0	98.3	100.0
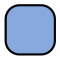 Posture	Pediatric	1	99.4	99.9	100.0
	Adult	1	35.4	72.2	85.3
		2	84.9	94.8	95.7
		3	93.0	95.1	99.3
		4	94.4	97.8	99.9
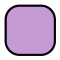 Rheology	Pediatric	1	100.0	100.0	100.0
	Adult	1	76.0	92.9	92.8
		2	87.9	95.6	99.5
		3	94.9	99.8	99.9
		4	98.2	99.9	100.0

In the pediatric model, gains in coverage are confined almost entirely to the lowest-volume regime, where the additional control levers recover a few extra points relative to the baseline, especially for 2 and 4 aliquots. Beyond this regime, however, the simplest baseline scenario is already sufficient to reach near-maximal performance, so further increases in control complexity yield little or no additional benefit in final coverage.

Adult behavior differs more markedly. The model remains substantially sensitive to increased scenario complexity, particularly in the most constrained low-volume regime. At a target volume of 1mL/kg, all three enriched scenarios improve coverage relative to the baseline, with the largest gains obtained for the rheology-enabled setting. These gains then decrease progressively as prescribed volume increases and the baseline protocol itself approaches saturation. Consistently, the adult–pediatric gap narrows substantially under scenario enrichment at low volume: at 1mL/kg, it decreases from a baseline value of about 73 points to about 24 points for 2 aliquots when rheology optimization is enabled, and from about 35−40 points to about 7 points for 4–8 aliquots under the same scenario. At higher prescribed volumes, this gap becomes much smaller as both scales approach ceiling performance.

Efficiency trends in Fig 10b, 10e show that these gains are driven by a better conversion of instilled material into useful distal delivery. At both scales, additional control levers increase efficiency relative to the baseline. In the pediatric model, the efficiency gain remains modest because the simplest scenario is already highly efficient, whereas in adults it becomes more pronounced, especially as fractionation increases. The richer control scenarios therefore raise the final coverage score by improving the delivery process itself, particularly where the symmetric adult baseline remains suboptimal.

The volume trends in Fig 10c, 10f reinforce the same interpretation. In the pediatric model, total volume remains essentially unchanged relative to the baseline, consistent with the fact that performance is already near ceiling and leaves little room for volumetric refinement. In adults, by contrast, the more complex scenarios achieve their gains while requiring no extra volume and often less. Thus, increased scenario complexity improves performance not only in terms of coverage but also in terms of volumetric economy.

Overall, the benefit of added control complexity depends strongly on how constrained the underlying delivery problem already is. In pediatric symmetric trees, the benefit remains marginal because the baseline protocol already operates close to the attainable ceiling. In adults, the same additional levers remain useful over a broader range, with the largest improvements observed in the low-volume regime, where the delivery problem is mechanically most challenging.

The rheology-enabled scenario provides the strongest and most consistent gains, with rheology tuning acting as a complementary lever alongside mechanical optimization. By contrast, enabling posture does not systematically improve coverage beyond what is already achieved by per-dose optimization. This likely reflects the fact that the predefined alternating posture sequence used in the baseline protocol is already close to mechanically favorable in a symmetric tree. Once per-dose volume and flow-rate control are introduced, the additional benefit of explicitly optimizing posture therefore remains limited in this setting. Moreover, several posture schedules may yield very similar rewards in the symmetric geometry, so independent trainings can converge to distinct near-equivalent solutions. When posture-enabled protocols yield slightly lower coverage, this appears to reflect either a small rebalancing of borderline branches under improved efficiency and volume usage, or convergence to a nearby quasi-equivalent posture solution, rather than a substantial deterioration of overall delivery quality. This point is important for interpreting the asymmetric results below, where posture control becomes more clearly differentiated.

**Fig 11:**
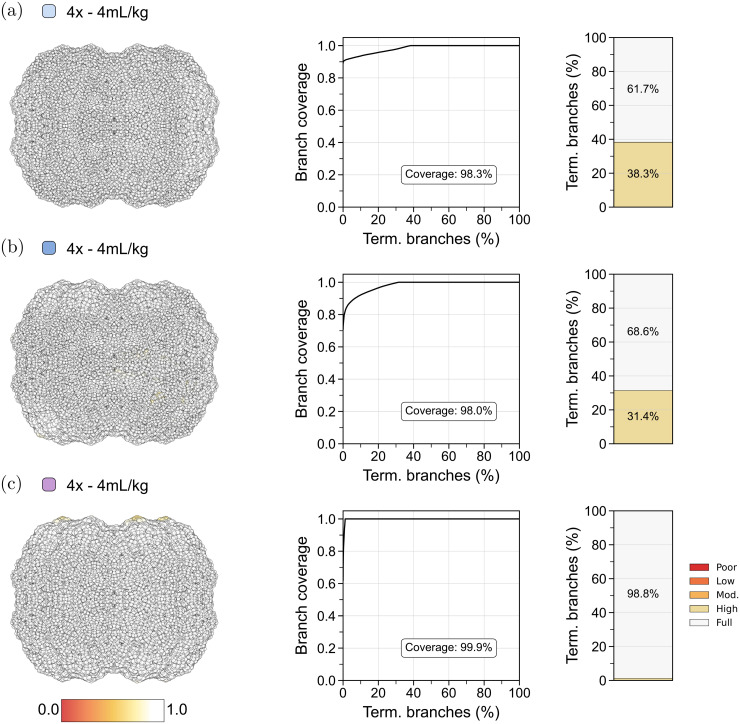
Representative branch-level score patterns under the optimized (a) Per-dose, (b) Per-dose + Posture, and (c) Per-dose + Posture + Rheology protocols in the reference symmetric airway models. All panels pertain to the adult model, 4 aliquots, target volume $V_{target}=4\;mL/kg$. Same conventions as in Fig 7.

A typical progression is illustrated in Fig 11, which compares representative adult symmetric cases at 4 aliquots and 4mL/kg under the Per-dose, Per-dose + Posture, and Per-dose + Posture + Rheology scenarios. The per-dose and posture-enabled scenarios already operate in a high-coverage regime, but still retain a visible low-score tail and a substantial fraction of terminal branches outside the full-coverage class. By contrast, the rheology-enabled scenario nearly eliminates this residual tail and shifts almost the entire branch population into the full-coverage regime. Thus, even in an already favorable symmetric adult setting, the most complex scenario visibly refines branch-level delivery by removing the remaining poorly matched regions.

**Fig 12:**
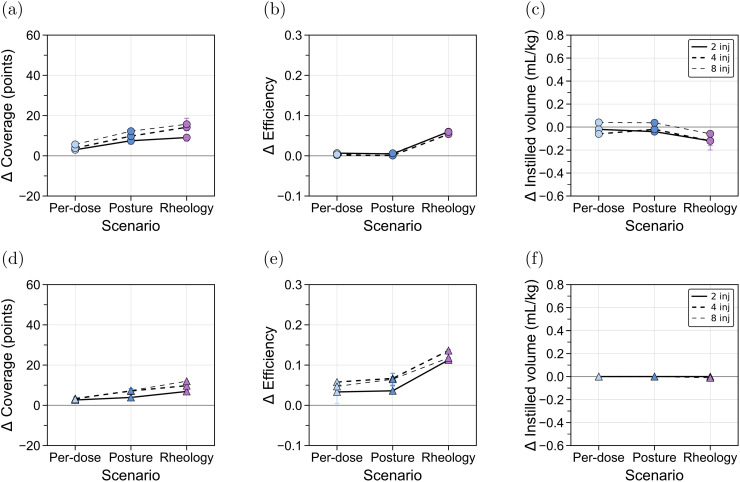
Optimized protocols in the highly asymmetric airway models: effect of prescribed target volume $V_{target}$, stratified by aliquot number and scenario complexity, on (a,d) the coverage score $S_{c}$, (b,e) the efficiency term $S_{e}$, and (c,f) the total instilled volume, shown as differences relative to the baseline scenario. Marker positions indicate medians, and error bars indicate the interquartile range (Q1–Q3) across the five airway realizations (seeds 1–5; see Table 1). Panels **(a)**–**(c)** correspond to the pediatric group at 2mL/kg, and panels **(d)**–**(f)** to the adult group at 4mL/kg. Improvement therefore corresponds to positive values in (a,b,d,e) and to negative values in **(c,f)**. Scenario colors follow Table 3: Baseline (gray), Per-dose (light blue), Per-dose + Posture (blue), and Per-dose + Posture + Rheology (purple).

**Effect of scenario complexity in highly asymmetric models.** We then turn to the highly asymmetric setting, where the same staged design probes whether additional control levers can compensate for geometric imbalance. To focus on mechanically feasible conditions where meaningful recovery is possible without re-entering a clearly underdosed regime, we report pediatric results at a target volume of $V_{target}=2\;mL/kg$ and adult results at 4mL/kg. Coverage scores are summarized in Table 10, and Fig 12 reports the corresponding changes relative to the baseline protocol for coverage, efficiency, and total instilled volume. Additional asymmetric results are provided in S1 Text, including the mildly asymmetric cases (Section 7.3.2) and supplementary metrics in the highly asymmetric setting (Section 7.3.3).

**Table 10:** Optimal coverage scores achieved in the highly asymmetric airway models, using a staged scenario design in which each scenario adds one additional control lever (Baseline → Per-dose → Per-dose + Posture → Per-dose + Posture + Rheology). Pediatric and adult results are shown at 2mL/kg (2 mL) and 4mL/kg (280 mL), respectively, corresponding to mechanically feasible regimes in the asymmetric setting.

Scenario	Group	Target vol. [mL/kg]	No. aliquots		
			2×	4×	8×
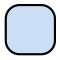 Per-dose	Pediatric	2	73.5 [3.6]	77.0 [3.8]	82.9 [2.0]
	Adult	4	56.3 [1.9]	59.4 [2.1]	62.7 [2.7]
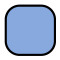 Posture	Pediatric	2	78.9 [0.3]	83.7 [1.0]	90.9 [1.4]
	Adult	4	57.6 [0.4]	62.0 [1.2]	66.8 [0.5]
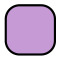 Rheology	Pediatric	2	80.8 [1.0]	89.3 [1.2]	95.0 [0.2]
	Adult	4	60.5 [0.9]	65.5 [0.2]	70.3 [0.6]

Under strong asymmetry, the scenarios become more clearly discriminant than in the symmetric reference geometry. At both scales, coverage improves essentially monotonically as control progresses from per-dose optimization to posture-enabled and then rheology-enabled protocols, showing that the additional control levers remain useful once geometric imbalance makes delivery more challenging. The corresponding IQRs remain small, indicating that this scenario ordering is consistently recovered despite anatomy-dependent variability, even though the optimized protocols themselves may differ between airway-tree realizations. The ordering is especially clear in the pediatric model, where the gain over baseline reaches about 8−15 points in the rheology-enabled setting, depending on aliquot number, compared with more modest gains under per-dose optimization alone. In adults, the same hierarchy is preserved, although with smaller absolute increments, reflecting the fact that even the most complex scenario only partially compensates for the strong geometric limitations of the highly asymmetric tree.

Despite these gains, the adult–pediatric gap remains substantial in the fixed-volume asymmetric regimes considered here and is not eliminated by increased control flexibility. For example, across the 2-, 4-, and 8-aliquot settings, it remains about 17−21 points in the baseline case and about 20−25 points even when rheology optimization is enabled. Although both scales benefit from the additional control levers, the pediatric model retains a higher absolute coverage level, indicating that additional control flexibility mitigates but does not remove the underlying scale dependence.

A key difference from the symmetric case is that posture control becomes genuinely useful under asymmetry. Unlike in the symmetric geometry, enabling posture now yields additional coverage gains beyond per-dose optimization alone at both scales and for all aliquot numbers, indicating that geometric imbalance makes body orientation a more informative control variable. Strong asymmetry therefore increases the value of posture as a control lever, making its contribution more clearly distinguishable from that of dose partitioning and flow-rate adjustment.

The efficiency changes in Fig 12b, 12e provide a complementary view of this hierarchy. As in the symmetric case, the more complex scenarios improve the conversion of instilled material into useful distal delivery, and the rheology-enabled scenario again provides the strongest gains. These improvements are moderate in absolute magnitude, but they are systematic and closely parallel the coverage hierarchy. By contrast, the total instilled volume changes little, especially in adults, where the optimized solutions remain close to the prescribed target volume. In this strongly constrained regime, overinstillation is no longer an effective way to recover enough coverage to compensate for the associated penalty. Thus, under strong asymmetry, the main benefit of additional control flexibility comes primarily from improved delivery quality and efficiency rather than from a substantial change in the instilled volume budget.

**Fig 13:**
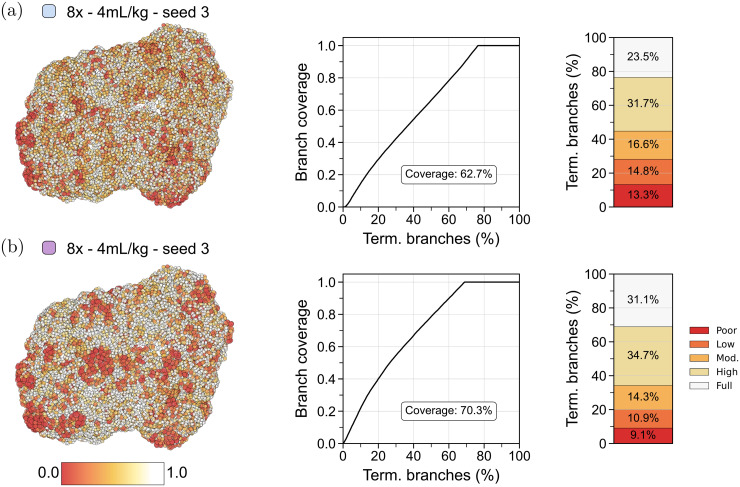
Representative branch-level score patterns under the optimized (a) Per-dose and (b) Per-dose + Posture + Rheology protocols in the highly asymmetric airway models. All panels pertain to the adult model, 8 aliquots, target volume $V_{target}=4\;mL/kg$. Same conventions as in Fig 7.

The branch-level maps in Fig 13 make this trade-off more explicit. The rheology-enabled scenario yields the best global coverage, but the improvement is not pointwise uniform: some regions that are moderately well covered under simpler scenarios can become less favorable under more complex ones, even as the overall score increases. The net gain therefore reflects a spatial rebalancing of delivery across the tree rather than a uniform improvement in every local region.

Overall, strong asymmetry amplifies the separation between scenarios and changes the relative value of the available control levers. Per-dose optimization remains beneficial, posture control becomes clearly informative, and the rheology-enabled scenario provides the largest aggregate gain. However, these gains remain partial, indicating that additional control flexibility mitigates but does not eliminate the geometric constraints imposed by strong asymmetry.

### Per-aliquot mechanistic diagnostics

**Fig 14:**
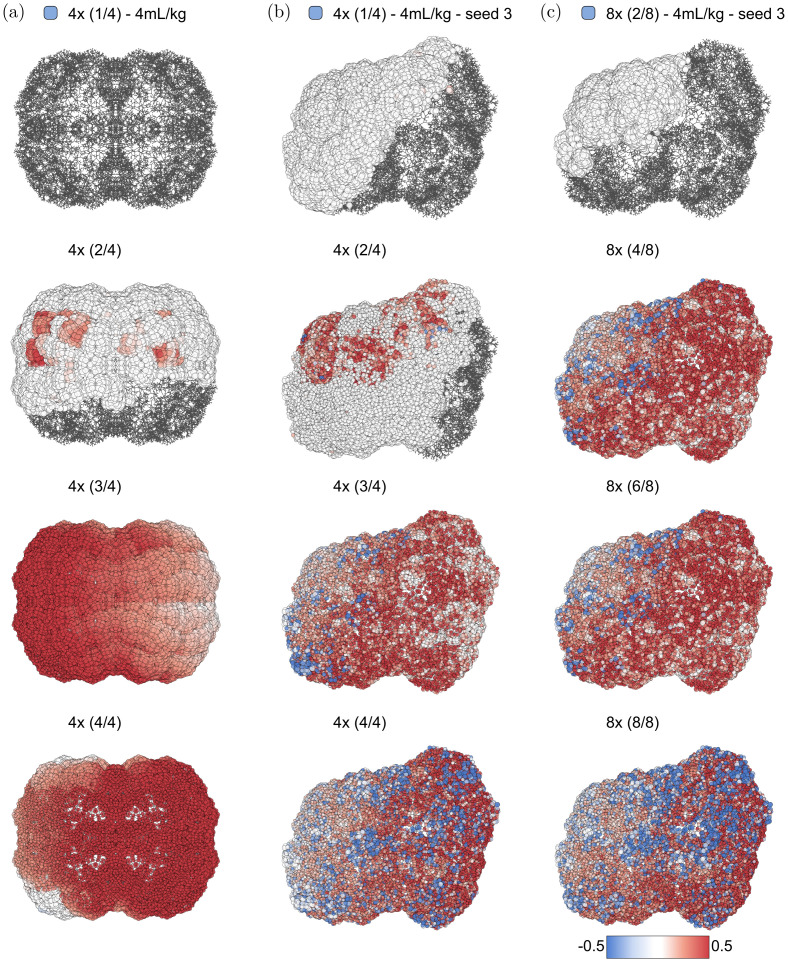
Aliquot-resolved branch-level coverage changes under the adult optimized Per-dose + Posture protocol. **(a)** Symmetric airway model, 4 aliquots, target volume $V_{target}=4\;mL/kg$. **(b)** Highly asymmetric airway model, 4 aliquots, 4mL/kg. In **(a,b)**, colors indicate the signed change in local coverage between successive aliquots, with warm (resp. cool) colors denoting local improvement (resp. degradation). **(c)** Highly asymmetric airway model, 8 aliquots, 4mL/kg, shown as cumulative changes over successive two-aliquot blocks (2/8, 4/8, 6/8, 8/8).

To further interpret the optimized protocols, we examine aliquot-resolved changes in branch-level coverage for representative adult Posture protocols in the symmetric and highly asymmetric geometries. Rather than reporting absolute coverage alone, Fig 14 shows the local coverage-score change accumulated over successive aliquot blocks, providing a mechanistic readout of how the protocol redistributes delivery throughout the sequence. In both geometries, the early aliquots primarily establish distal access, while later aliquots increasingly refine branch-level redistribution. Branch-level scores begin to rise more substantially only once this initial access phase has been completed, typically beyond the first half of the sequence. In the symmetric case, the progression remains largely cumulative: distal reach is completed rapidly, and successive aliquots improve coverage over broad regions with only limited evidence of persistent local regressions. By contrast, in the highly asymmetric case, local improvement is no longer monotone. Some regions improve while others become less well covered than at earlier stages, even though the final global score is higher. This reflects the coupled, terminal nature of the objective over the full protocol: intermediate or late aliquots may trade local errors against improved global redistribution and outlier correction, rather than improving every branch in isolation. Overall, these maps indicate that learned schedules act through sequential completion of distal reach and constrained spatial reallocation, rather than through a strictly monotone “each aliquot improves everything” behavior. The more highly fractionated 8-aliquot protocol follows the same logic: intermediate states can remain comparable to those observed under 4-fold fractionation, but the additional late-stage correction steps improve the final branch-level outcome.

### Cross-scenario consistency of optimized protocols

Beyond aggregate performance metrics, the structure of the optimized protocols provides physical insight into how the agent coordinates dose allocation, flow-rate scheduling, and posture adjustments across anatomically diverse airway trees while balancing branch-level coverage against excessive total instilled volume. We summarize below how these representative patterns depend on scenario complexity in symmetric and asymmetric adult and pediatric models.

**Dose-volume and flow-rate patterns across the instillation sequence.** We first characterize the temporal organization of each optimized protocol by examining how relative volume and flow-rate allocation vary across the injection sequence. For each protocol, the dose-wise injected volume and flow rate are normalized by their protocol-wise totals, yielding per-dose fractions. A linear fit of these fractions as a function of normalized dose index is then used to define a trend for volume and flow-rate allocation. Positive (resp. negative) slopes are classified as increasing (resp. decreasing). Combining both classifications yields a categorical descriptor of each protocol, with the analysis below focusing on the prevalence of protocols in which both injected volume fraction and flow-rate fraction increase across the sequence (increasing/increasing).

**Fig 15:**
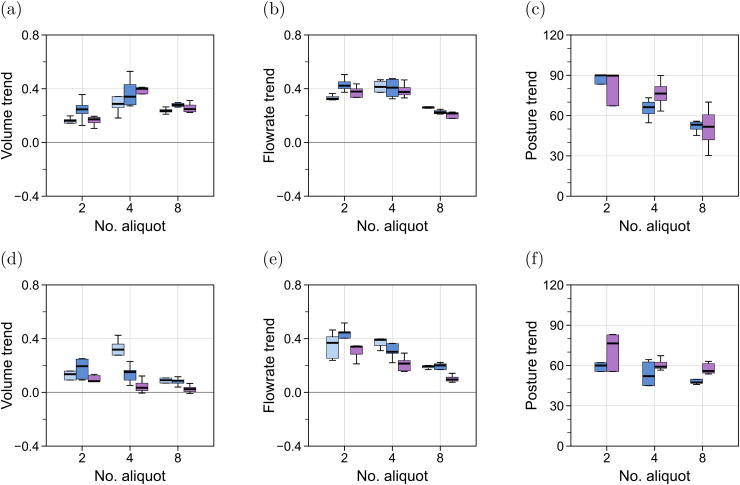
Temporal allocation trends in the adult optimized protocols: distribution of the fitted trend values obtained from dose-wise (a,d) injected-volume fractions, (b,e) flow-rate fractions, and (c,f) posture-trend values over the aliquot sequence, shown for the (a–c) symmetric and (d–f) highly asymmetric airway models. Protocols for which both fitted volume and flow-rate trends are positive are classified as increasing/increasing, corresponding to joint increases in injected-volume and flow-rate fractions across aliquots. Posture trends are evaluated relative to the baseline alternating LLD/RLD sequence at the level of delivery-plane orientation.

**Fig 16:**
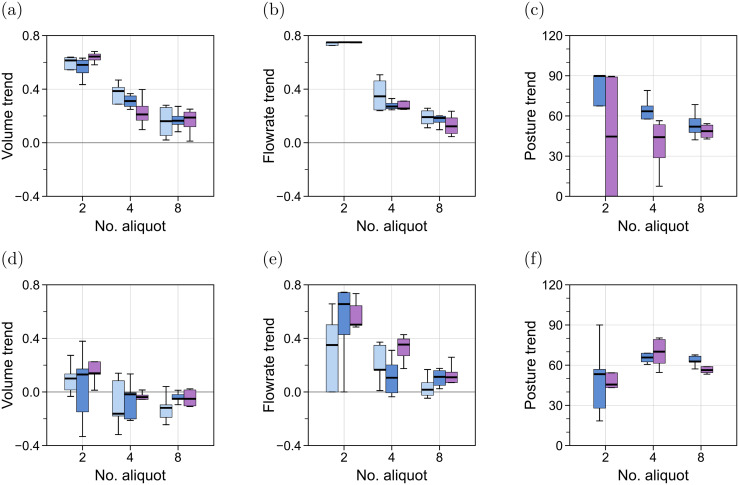
Same as Fig 15 for the pediatric optimized protocols.

Quantitative results are reported in Table 11, while the distribution of volume- and flow-rate-allocation trends is shown in Figs 15a, 15b and 16a, 16b for adult and pediatric models, respectively. In adults, the increasing-volume / increasing-flow-rate pattern remains highly robust across scenarios, with frequencies ranging from 92.9 % to 100.0 % depending on fractionation. Posture scheduling preserves this motif in all adult cases, whereas rheology introduces only a modest decrease in robustness, most noticeably at 8 aliquots (78.6 %). In pediatrics, by contrast, this robustness is markedly reduced. The increasing/increasing pattern is observed in only 42.9–64.3 % of protocols under per-dose optimization, 35.7–85.7 % when posture control is enabled, and 35.7–92.9 % when rheology optimization is added, with a noticeable loss of robustness beyond 2 aliquots. This likely reflects the fact that a less anatomically constrained environment allows a much broader set of optimal strategies to emerge. Similar deposition performance can therefore be achieved through more diverse temporal organizations of dose volume and flow rate.

**Table 11:** Frequency of the increasing/increasing temporal motif in the optimized protocols. Values report the percentage of protocols for which both the injected-volume fraction and the flow-rate fraction increase over the aliquot sequence. All entries are based on the same sample size (*n* = 14 protocols per group and scenario).

Group	Scenario	No. aliquots		
		2×	4×	8×
Adult	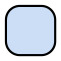 Per-dose	92.9 %	100.0 %	92.9 %
	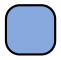 Posture	100.0 %	100.0 %	100.0 %
	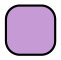 Rheology	92.9 %	85.7 %	78.6 %
Pediatric	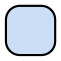 Per-dose	64.3 %	57.1 %	42.9 %
	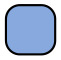 Posture	85.7 %	50.0 %	35.7 %
	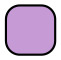 Rheology	92.9 %	35.7 %	42.9 %

These cross-scenario trends provide a mechanistic readout that supports an interpretation of the optimized schedules in terms of model-resolved transport mechanisms. Progressively increasing aliquot volume and flow rate suggests that early instillations are used preferentially to establish distal access while limiting proximal coating losses, whereas later, larger and faster aliquots can place more emphasis on downstream splitting and redistribution once the most accessible proximal surfaces have already been coated. This is consistent with earlier modeling results showing that lower flow rates tend to favor delivery efficiency, whereas higher flow rates improve redistribution and homogenization through enhanced splitting dynamics [19]. In that sense, the robust adult increasing/increasing motif is not only a statistical regularity, but also a plausible transport-level strategy for balancing early distal penetration against later homogenization over the course of the sequence.

**Posture schedules.** We then examine whether optimized protocols also exhibit a robust temporal organization in patient orientation across the injection sequence. To do so, each protocol is compared against the baseline alternating left–right lateral decubitus sequence (LLD/RLD) across aliquots. Because physically equivalent (γ,ϕ) pairs can describe the same delivery plane, posture differences are evaluated at the level of plane orientation rather than raw angular coordinates. For each optimized protocol, we then quantify posture trend from the sequence-wise evolution of this relative postural organization across aliquots. Since posture angles were discretized on a 15° grid, these trends should be interpreted as coarse spatial organization rather than fine continuous adjustments.

The corresponding distributions are shown in Figs 15 and 16. In contrast to volume and flow-rate allocation, posture does not display a comparably conserved sequential motif across scenarios. Posture-trend values remain of similar magnitude in all adult settings, with median values around 60, corresponding roughly to four angular discretization steps, and IQRs of about 20. This suggests that posture adjustments remain useful, but are not constrained to a uniquely conserved temporal pattern. Comparable values are also observed in pediatric settings, but the associated distributions are substantially more heterogeneous, with stronger dependence on anatomical group, symmetry class, and aliquot count than in adults. A particularly broad spread is observed for the pediatric symmetric case in the rheology-enabled setting, the least constrained scenario, in which multiple posture-scheduling strategies may support similarly effective deposition patterns.

Overall, these results suggest that posture scheduling contributes to optimization by exploiting gravitational redistribution, but not with the same structural robustness as dose-volume and flow-rate scheduling. Whereas volume and flow-rate trends define a highly reproducible sequential motif in adults, posture appears to play a more context-dependent compensatory role, with multiple distinct spatial organizations capable of achieving comparable deposition outcomes.

**Formulation trends.** Allowing rheological parameters to vary reveals consistent patterns across adult geometries. Optimized solutions remain within the prescribed plausibility bounds, but may approach them; this is interpreted as an informative transport-level trade-off rather than a numerical artefact. In particular, they tend to favor higher surface tension (often near the upper bound, σ=40 dyn/cm) together with lower density. This should not be interpreted as contradicting the clinical role of surfactant: in the present plug-transport model, σ is a constant effective parameter entering capillary-pressure jumps and coating via the capillary number. Lowering σ increases ${Ca}_{p}$, thickens the trailing film, and increases coating losses, which can reduce the volume reaching distal regions; conversely, higher σ mitigates coating and is therefore associated with improved downstream propagation and splitting in the airway-transport regime. The selected values remain surface-active relative to the carrier fluid and lie within the range explored in prior surfactant-transport studies [50].

In addition, rheology optimization selects viscosities systematically near the lower bound of the explored admissible range (μ=10 cP). This behavior is informative when compared with earlier modeling results, which associated very low viscosity with poor homogenization under much more constrained delivery protocols and used that observation to help rationalize the inferior performance of low-viscosity synthetic surfactants such as Exosurf relative to animal-derived preparations [19]. The present results do not directly contradict that interpretation, but suggest that it may not be universal once delivery mechanics are sufficiently adaptive. Under fixed or weakly adaptable protocols, low viscosity may indeed degrade global tree-scale mixing. However, under jointly optimized aliquot partitioning, flow-rate control, and posture scheduling, it can instead favor distal access, branch-level coverage, and delivery efficiency.

The present low-viscosity optimum should not be read as a prescriptive statement about clinical surfactant selection, but rather as evidence that material properties and delivery mechanics act as coupled components of a shared optimization landscape rather than as independent levers. In that sense, the apparent disadvantage of low-viscosity formulations may be protocol-dependent rather than purely intrinsic. More generally, the ranking of formulations may depend strongly on which aspect of delivery quality is prioritized and how it is quantified, a point revisited below when comparing global and branch-level metrics more explicitly.

From a practical standpoint, rheological tuning should therefore be viewed as a complementary experimental lever rather than as a substitute for mechanical control. Because surfactant additives are known to modify surface activity and rheological properties, notably viscosity, *in vitro* and *in vivo* [56], they provide a plausible route for benchtop testing of the coupled effects of formulation and protocol suggested by the present optimization framework.

### Limitations of homogeneity-based delivery metrics and the role of asymmetry

The preceding sections show that optimized instillation protocols can substantially improve surfactant access to distal regions. However, the interpretation of these gains depends strongly on the metric used to quantify delivery quality. The choice of an evaluation metric defines the delivery objective being assessed. Branch-level coverage is not intended as a universally superior deposition metric, but provides a task-specific measure of satisfactory distal dosing by assessing whether a sufficiently broad fraction of terminal branches receives an amount within a functionally acceptable range. Homogeneity-based metrics are not alternative definitions of the same objective; they answer a different and stricter question, namely whether the achieved deposition pattern approaches uniformity across terminal branches. These quantities are therefore not equivalent: a protocol may achieve high distal coverage while still leaving substantial residual imbalance across branches. Low homogeneity scores do not necessarily imply that protocol optimization failed to improve distal access; rather, they indicate that near-uniform delivery remains difficult to achieve and may be poorly discriminating in strongly asymmetric geometries. Additional comparisons reported in S1 Text (Section 9) confirm quantitatively that these metrics are related, but not interchangeable.

**Fig 17:**
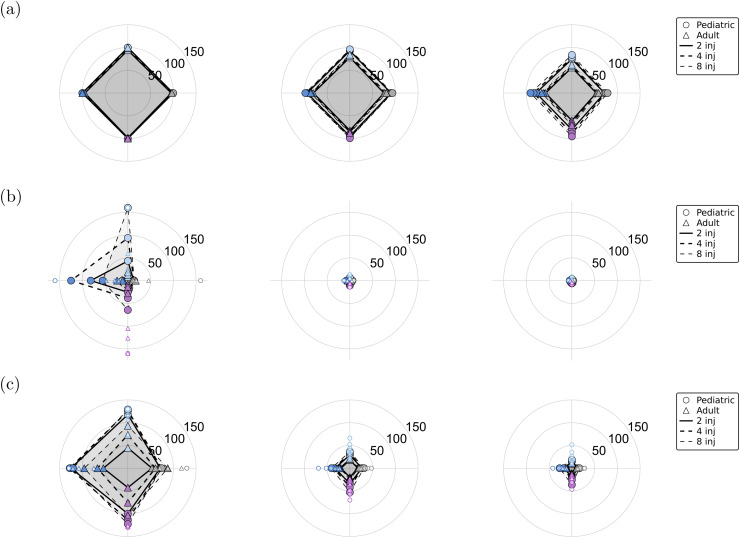
Spider-chart summary of global performance across optimization protocols, stratified by aliquot number and scenario complexity, for (a) coverage, (b) the RMS deviation-based global homogeneity score, and (c) the branch-resolved homogeneity score defined in S1 Text. From left to right: symmetric, mildly asymmetric, and highly asymmetric models. Open symbols in (b,c) denote homogeneity values evaluated a posteriori on the coverage-optimized protocols, whereas filled symbols denote reduced-budget direct-optimization runs for the corresponding metric. Coverage and branch-resolved homogeneity are shown on their original score scales, whereas global homogeneity is unbounded and therefore capped at 160 for visualization purposes.

This distinction is illustrated in Fig 17, which compares coverage with two homogeneity-based diagnostics across optimization scenarios and asymmetry levels: a global homogeneity score computed at the scale of the whole tree, identical to the standard homogeneity metric widely used in the literature [19], and a branch-resolved homogeneity score computed at the branch level, intended to provide an intermediate criterion between coverage and global homogeneity (see S1 Text, Section 8). Empty symbols correspond to homogeneity values computed a posteriori from the coverage-optimized protocols, whereas filled symbols correspond to reduced-budget direct-optimization runs (150 episodes) aimed at optimizing homogeneity itself.

We first consider the global homogeneity metric, because this type of score has often been used to argue that surfactant instillation in adults is intrinsically inefficient. The present results in Fig 17(b) show that such a conclusion can be misleading. Even in symmetric geometries, global homogeneity already begins to decouple from coverage under coverage-optimized protocols, especially in pediatric cases, where high distal coverage can be achieved without strongly constraining tree-scale uniformity. As asymmetry increases, the divergence becomes much stronger: coverage remains comparatively stable and continues to improve with scenario complexity, whereas global homogeneity collapses, even in pediatric settings, where values fall to very low levels only marginally above those observed in adults. This is difficult to reconcile with the well-established clinical efficacy of surfactant replacement in comparable pediatric settings. In that sense, global homogeneity yields a markedly more pessimistic picture of delivery success than coverage. This discrepancy suggests that, once distal access is broadly achieved, global homogeneity may become too sensitive to residual imbalance to serve on its own as a robust indicator of functional delivery quality.

The direct-optimization controls help explain why this occurs. In symmetric models, global homogeneity recovers substantially when optimized explicitly, especially in pediatric settings. This shows that low values under the coverage-driven reward partly reflect objective mismatch: a protocol can be highly effective in terms of distal access without producing globally uniform deposition. In asymmetric models, however, the directly optimized values remain low, only modestly above their coverage-optimized counterparts, indicating that most of the degradation is structural and reflects the intrinsic difficulty of maintaining tree-scale homogeneity in highly asymmetric regimes. Global homogeneity is therefore not simply a harsher version of coverage; it is also a more fragile metric, whose low values combine true delivery limitations with a strong sensitivity to local outliers and residual imbalance.

This has an important interpretive consequence. Low global-homogeneity values should not be read automatically as evidence that surfactant delivery “does not work”. Rather, they indicate that globally uniform redistribution across the whole tree becomes difficult to preserve once the protocol is required to maintain efficient distal access in a geometrically constrained tree. Richer optimization scenarios partly mitigate this drop, suggesting that additional control flexibility can reduce the mismatch between coverage and global homogeneity, but they do not eliminate it. Coverage therefore provides a functional indicator aligned with the delivery objective targeted here: whether a broad distal population remains sufficiently served despite residual local imbalance.

A more permissive picture emerges when homogeneity is evaluated at the branch level rather than at the scale of the whole tree. Branch-resolved homogeneity in Fig 17(c) remains better aligned with coverage, so the discrepancy between coverage-optimized and directly optimized values is smaller. Nevertheless, it still degrades substantially with asymmetry, indicating that improved metric alignment is not sufficient to prevent a real loss of distributional uniformity under geometrically constrained delivery. Taken together, these results suggest that homogeneity metrics remain informative as complementary diagnostics, but that global homogeneity in particular is too sensitive to serve on its own as a reliable marker of delivery success in adult asymmetric trees.

The branch-level coverage-based scoring adopted here addresses these limitations by providing a functionally oriented and physically meaningful assessment of deposition quality across heterogeneous lungs. Using this criterion, pediatric asymmetric trees recover high scores consistent with the robust delivery expected in clinically successful pediatric settings, thereby resolving the apparent paradox created by global homogeneity metrics. In adults, optimized protocols likewise recover substantially higher and more interpretable performance levels, while highly asymmetric geometries under standard protocols still highlight genuine challenges in achieving broadly homogeneous coating. In that sense, coverage does not remove the underlying difficulty of adult delivery, but it provides a clearer basis for distinguishing true transport limitations from artefacts of overly fragile summary metrics.

## Conclusion

**Summary.** In this study, we introduced a computational framework that couples mechanistic modeling of surfactant propagation in morphometrically plausible airway trees with reinforcement learning-based optimization of surfactant replacement therapy protocols. Unlike prior computational studies based on parameter sweeps, our framework learns delivery policies directly from a physics-based model that captures the fluid-mechanical determinants of surfactant transport. This illustrates how AI-assisted optimization can complement mechanistic modeling, both to generate and evaluate candidate administration schemes within a controlled computational setting and to probe the controllability of complex biophysical processes.

Our results show that joint optimization of aliquot volume, flow rate, and patient posture markedly improves branch-level delivery performance across anatomically diverse airway trees, yielding protocols that, within this *in silico* framework, substantially improve predicted adult-scale distal delivery. Extending the control space to include surfactant rheology—particularly viscosity—provides complementary levers to improve performance while maintaining physiologically realistic instillation volumes. Together, these findings show that delivery performance depends strongly on geometry, prescribed volume, and control complexity, and that additional mechanical and material levers can expand the set of attainable adult-scale delivery patterns without removing the structural limitations imposed by strong asymmetry.

Across the tested scenarios, the results reveal a continuum of feasible regimes linking control complexity, delivery efficiency, and formulation. Increasing control freedom—through finer dose partitioning, posture adaptation, and, when included, rheology tuning—improves branch-level delivery performance without necessarily increasing total instilled volume. In this sense, richer protocols do not simply deliver more surfactant; they improve how effectively the administered dose is routed and redistributed through the tree. The rheology-enabled setting further indicates that material properties and delivery mechanics should be interpreted as coupled rather than independent levers: within the present framework, formulations that appear less favorable under fixed protocols may perform more favorably once delivery is jointly optimized.

**Implications.** Although the optimized metrics are not intended as clinical outcome surrogates, they serve as mechanical delivery proxies designed to reflect clinically motivated objectives for instilled therapies by capturing (i) broad distal reach across the airway network, (ii) sufficiently widespread delivery with adequate deposited mass in targeted regions, and (iii) robustness to localized maldistribution, i.e., preventing the score from being dominated by a small subset of outlier branches. This perspective underscores the limitations of stricter homogeneity-based metrics for SRT assessment, especially when applied at the scale of the whole tree, where they may fail to capture the interplay between airway geometry, plug dynamics, and surfactant distribution that ultimately governs mechanically achievable delivery in adults. Within this perspective, our branch-level coverage metric provides a functionally oriented assessment of deposition quality across heterogeneous lung structures: it evaluates whether delivery remains locally acceptable across a sufficiently broad distal region, rather than whether the full deposition pattern approaches near-uniformity. This makes it possible to compare anatomies and interpret delivery gains in structurally challenging cases where stricter homogeneity metrics may collapse despite improved distal access.

**Limitations and outlook.** The present formulation remains a simplified representation of pulmonary fluid mechanics, in line with the level of abstraction typically used in organ-scale instillation models. The airway transport model is intentionally reduced (rigid cylindrical branches, constant diameter per branch, fixed L/D scaling, idealized branching geometry, and quasi-static algebraic recursion) to enable systematic protocol optimization over large control spaces. Absolute performance values should therefore be interpreted within this modeling envelope, and fine details of the optimized schedules (exact switching times, intermediate roll angles, or precise aliquot partitioning) should not be viewed as uniquely optimal or directly prescriptive. Nevertheless, the main conclusions rely on lever-by-lever performance shifts and coarse spatial signatures driven by mechanisms expected to persist under more detailed airway mechanics: gravity-driven redistribution under posture changes, the trade-off between proximal coating losses and distal penetration under flow modulation, and reduced step-to-step maldistribution under dose fractionation. Accordingly, we emphasize qualitative, mechanistically interpretable trends and staged ablations over exact protocol realizations.

The omitted physiological mechanisms should not all be interpreted as small perturbations of the present results. Some model refinements, such as modest changes in geometric scaling or local frictional closure, may primarily shift absolute score values or the volume thresholds at which high-coverage regimes become accessible. By contrast, airway compliance and recruitment, ventilation–liquid coupling, mucus transport, disease-specific obstruction, dynamic breathing mechanics, patient-specific morphology, and *in vivo* variability could alter redistribution pathways more substantially, thereby changing the relative ranking and practical feasibility of posture control, flow scheduling, aliquot partitioning, or rheology in specific physiological settings. These factors should therefore be tested in targeted extensions. As high-resolution morphometric datasets become available, an additional direction will be to stratify patient-specific airway trees and deposition responses using clustering and representation-learning methods for structured data, to organize anatomical variability and support consistent protocol evaluation across larger sets of anatomies [57,58]. Consequently, the detailed optimized schedules reported here should be viewed as optima within the present idealized environment, rather than as schedules expected to remain optimal under all model extensions. The more robust conclusion is methodological and mechanistic: surfactant administration can be posed as a constrained delivery-optimization problem in which airway geometry, plug transport, and controllable protocol variables interact. More realistic physiological descriptions would therefore redefine the simulation environment and its constraints, requiring re-optimization rather than direct transfer of the present protocols; the same model-environment plus RL framework can, however, be applied to those enriched settings.

The present open-loop formulation commits to a multi-aliquot sequence in advance; it therefore identifies the best fixed sequence under the model assumptions, but cannot revise the protocol once delivery has started. More adaptive formulations exploiting information acquired during administration could operate at different granularities, ranging from receding-horizon updates using inter-aliquot delivery states (e.g., partial coating or coverage maps, distal delivered volumes, or local flow distributions) to finer closed-loop control within an aliquot, if suitable real-time feedback were available. In modeling terms, such variants would redefine the learning problem by introducing sequential credit assignment and requiring explicit assumptions about which intermediate quantities are realistically observable. They could also produce different model-optimal protocols, but would not necessarily dominate the present sequence-level optimum in all settings, because optimizing the full sequence at once can account for cases in which early aliquots are not individually optimal but help establish distal access or redistribution patterns that improve the final delivery outcome. Their relevance in realistic clinical settings would remain conditional on the availability of reliable and timely feedback signals, on whether these signals can be interpreted as delivery information, and on which control inputs can safely and practically be modified during administration.

Finally, the present study focuses on mechanical feasibility rather than clinical efficacy. In adult ARDS, outcomes reflect the interplay of delivery with biophysical inactivation and a heterogeneous, evolving inflammatory milieu; therefore, improved deposition in a mechanistic model should not be interpreted as a direct surrogate for clinical benefit. Practical deployment of protocol levers is further constrained by ICU logistics and safety considerations, which may limit achievable posture modulation or flow profiles. Accordingly, the optimized protocols should be viewed as computational design hypotheses that stress-test delivery mechanics, not as candidate clinical protocols. The present results therefore provide a controlled feasibility benchmark. The predicted protocol trade-offs—and the associated mechanical delivery proxies used for optimization—are directly testable in controlled benchtop settings using additively manufactured multi-generation airway-tree replicas, where plug splitting and posture-dependent routing can be quantified experimentally [59]. Future work will pursue this experimental direction to anchor the *in silico* predictions, while extending the framework toward more clinically relevant scenarios, including multi-site bronchoscopic instillation, enriched disease-specific mechanics, and adaptive protocol variants.

Altogether, this framework unifies physics-based modeling with AI-assisted protocol optimization to guide the mechanistic evaluation of intrapulmonary liquid delivery strategies. Beyond surfactant replacement, it provides a methodological foundation for studying geometry-aware respiratory delivery, with future translational work to be informed by targeted experimental validation and the integration of bedside feasibility constraints.

## Supporting information

S1 Text Supplementary methods and analyses. (PDF)

S1 Fig Histograms of the asymmetry coefficient computed from a double-lobed Gaussian distribution for three pseudo-random seeds: (a) pediatric models (8 generations plus trachea) and (b) adult models (15 generations plus trachea). (TIF)

S2 Fig Mildly asymmetric trees corresponding to the five stochastic airway-tree realizations listed in the mildly asymmetric section of Table 1: (a) pediatric airway trees (8 generations plus trachea) and (b) adult airway trees (15 generations plus trachea), shown in side and bottom views. Airway narrowing across generations is encoded by line thickness, while color encodes the relative difference between the local diameter and that of the corresponding symmetric tree. For ease of comparison, all dimensions are normalized by the tracheal diameter of each model. (TIF)

S3 Fig Same as S2 Fig for the highly asymmetric trees corresponding to the five stochastic airway-tree realizations listed in the highly asymmetric section of Table 1. (TIF)

S4 FigIllustration of the score-shaping functions used in the reward definition.(a) Local branch-level coverage score $s_{c,i}$ as a function of the branch-wise ratio $x_{i}=v_{i}/v_{target,d}$, with dashed lines indicating the main coverage thresholds $\left(x_{\min},1,x_{\max}\right)$ and the shaded band showing the narrower conditional uniformity interval $\left(x_{u,min},x_{u,max}\right)$. (b) Global volume factor $S_{v}$ as a function of the total-volume ratio $X=V_{inst}/V_{target}$. All plots use the default parameters reported in S3 Table.(TIF)

S5 Fig Learning trajectories for a representative Per-dose + Posture scenario, four-aliquot pediatric optimization run. Panels (a–e) show the episode-wise evolution of the total reward and its components (coverage, efficiency, conditional uniformity, volume penalty). Thin lines: instantaneous values; thick lines: 10-episode moving averages. Panels (f–i) report the learned action parameters by aliquot: volume fractions, flow rates, pitch, and roll angles. (TIF)

S6 Fig Learning trajectories for a representative Per-dose + Posture scenario, four-aliquot adult optimization run. Panels (a–e) show the episode-wise evolution of the total reward and its components (coverage, efficiency, conditional uniformity, volume penalty). Thin lines: instantaneous values; thick lines: 10-episode moving averages. Panels (f–i) report the learned action parameters by aliquot: volume fractions, flow rates, pitch, and roll angles. (TIF)

S7 FigAdditional optimized baseline metrics in the reference symmetric airway models: effect of prescribed target volume $V_{target}$, stratified by aliquot number, on (a) the conditional uniformity score $S_{u}$ and (b) the fraction of terminal branches entering the calculation of $S_{u}$. Circles and triangles denote pediatric and adult groups, respectively; line styles denote aliquot number.(TIF)

S8 FigBaseline protocol in the mildly asymmetric airway models: effect of prescribed target volume $V_{target}$, stratified by aliquot number, on (a) the coverage score $S_{c}$, (b) the efficiency term $S_{e}$, and (c) the volume penalty term $S_{v}$. Marker positions indicate medians, and error bars indicate the interquartile range (Q1–Q3) across the five airway realizations (seeds 1–5; see Table 1).(TIF)

S9 FigAdditional optimized baseline metrics in the mildly asymmetric airway models: effect of prescribed target volume $V_{target}$, stratified by aliquot number, on (a) the conditional uniformity score $S_{u}$ and (b) the fraction of terminal branches entering the calculation of $S_{u}$.(TIF)

S10 FigAdditional optimized baseline metrics in the highly asymmetric airway models: effect of prescribed target volume $V_{target}$, stratified by aliquot number, on (a) the conditional uniformity score $S_{u}$ and (b) the fraction of terminal branches entering the calculation of $S_{u}$.(TIF)

S11 FigAdditional metrics for the staged scenario-complexity analysis in the reference symmetric airway models: effect of prescribed target volume $V_{target}$, stratified by aliquot number, on the conditional uniformity score $S_{u}$ and on the fraction of terminal branches entering the conditional uniformity calculation. Panels (a)–(b) correspond to the pediatric model and panels (c)–(d) to the adult model.(TIF)

S12 FigOptimized protocols in the mildly asymmetric airway models: effect of scenario complexity, stratified by aliquot number, on (a,d) the coverage score $S_{c}$, (b,e) the efficiency term $S_{e}$, and (c,f) the total instilled volume, shown as differences relative to the baseline scenario. Symbols indicate the median and error bars the interquartile range (Q1–Q3) across the five airway realizations (seeds 1–5; see Table 1). Panels (a)–(c) correspond to the pediatric model at 2mL/kg, and panels (d)–(f) to the adult model at 4mL/kg. Improvement therefore corresponds to positive values in (a,b,d,e) and to negative values in (c,f).(TIF)

S13 FigAdditional metrics for the staged scenario-complexity analysis in the mildly asymmetric airway models: effect of scenario complexity, stratified by aliquot number, on the conditional uniformity score $S_{u}$ and on the fraction of terminal branches entering the conditional uniformity calculation. Panels (a)–(b) correspond to the pediatric model at 2mL/kg, and panels (c)–(d) to the adult model at 4mL/kg.(TIF)

S14 FigAdditional metrics for the staged scenario-complexity analysis in the highly asymmetric airway models: effect of scenario complexity, stratified by aliquot number, on the conditional uniformity score $S_{u}$ and on the fraction of terminal branches entering the conditional uniformity calculation. Panels (a)–(b) correspond to the pediatric model at 2mL/kg, and panels (c)–(d) to the adult model at 4mL/kg.(TIF)

S15 Fig Representative local score patterns for (a) branch-level coverage and (b) branch-resolved homogeneity in a highly asymmetric airway model. For this case, the coverage score is 63.4, the branch-resolved homogeneity score is 11.2, and the global homogeneity score is 1.9, illustrating how increasingly stringent metrics yield progressively more pessimistic assessments of the same delivery pattern. (TIF)

S16 Fig Relationship between coverage and homogeneity metrics. Coverage versus (a) global homogeneity and (b) branch-resolved homogeneity, with both quantities normalized by their scenario-specific maxima. The identity line is shown for reference.
(TIF)

S17 Fig Relationship between coverage and homogeneity metrics. Residuals versus predicted values for bidirectional linear models relating coverage and homogeneity: (a) global homogeneity predicted from coverage, (b) coverage predicted from global homogeneity, (c) branch-resolved homogeneity predicted from coverage, and (d) coverage predicted from branch-resolved homogeneity. A residual cloud without visible structure indicates that the fitted relationship captures the main trend adequately, whereas systematic residual patterns suggest additional variability or bias not explained by the model.
(TIF)

S1 Table Fluids and parameter ranges used for calibration of the friction factors (datapoints of Zheng [16, 17]). (PDF)

S2 Table Summary of the PBO meta-parameters and network architectures. Only the hidden layer sizes are reported for each network architecture. (PDF)

S3 Table Numerical parameters used in the reward definition. (PDF)

S4 Table Numerical definitions of the reward variants used in the sensitivity analyses. Each variant family modifies only one group of reward parameters at a time: branch-level coverage thresholds, conditional uniformity window, or distal target fraction.
(PDF)

S5 TableSummary statistics for the pediatric airway model under the reference reward. Reported reward and score values are median [IQR] across 3 runs. Policy alignment is reported as the median [IQR] of the inter-run cosine similarities of the learned policies in normalized action space. Efficiency and conditional uniformity scores are reported as 100$S_{e}$ and 100$S_{u}$ for readability.(PDF)

S6 Table Summary statistics for the pediatric Per-dose + Posture scenario under the reward variants defined in S4 Table. Reported reward and score values are median [IQR] percentage variations relative to the reference setting, computed from variant-to-reference cross-comparisons across seeded runs. Policy alignment is reported as the median [IQR] cosine similarity between variant and reference policies in normalized action space; for each variant family, these values are computed after pooling the cross-comparisons from the corresponding loose and tight settings. (PDF)

S7 TableSummary statistics for the adult Per-dose + Posture scenario under the reference reward. Reported reward and score values are median [IQR] across 3 runs. Policy alignment is reported as the median [IQR] of the inter-run cosine similarities of the learned policies in normalized action space. Efficiency and conditional uniformity scores are reported as 100$S_{e}$ and 100$S_{u}$ for readability.(PDF)

S8 Table Summary statistics for the adult Per-dose + Posture scenario under the reward variants defined in S4 Table. Reported reward and score values are median [IQR] percentage variations relative to the reference setting, computed from variant-to-reference cross-comparisons across seeded runs. Policy alignment is reported as the median [IQR] cosine similarity between variant and reference policies in normalized action space; for each variant family, these values are computed after pooling the cross-comparisons from the corresponding loose and tight settings. (PDF)

S9 Table Coverage scores achieved under the optimized baseline protocol in the mildly asymmetric airway models. Values report the median [IQR] across the five airway realizations (seeds 1–5; see Table 1). The body-weight–normalized target-volume range of 1–4 mL/kg corresponds to 1–4 mL in the pediatric setting (1 kg neonate) and 70–280 mL in the adult setting (70 kg adult). (PDF)

S10 TableCoverage scores achieved under the optimized protocols in the mildly asymmetric airway models, using a staged scenario design where each scenario adds one additional lever (Baseline → Per-dose → Per-dose + Posture → Per-dose + Posture + Rheology). Pediatric and adult results are shown at 2mL/kg (2 mL) and 4mL/kg (280 mL), respectively, which corresponds to mechanically feasible regimes in the asymmetric setting.(PDF)

S11 Table Error thresholds and associated weights used for the branch-resolved homogeneity scoring. Each branch is assigned the highest weight corresponding to the smallest threshold it satisfies.
(PDF)

S12 TableStatistics comparing coverage and global homogeneity across scenarios. For each scenario, symmetric cases include *n* = 6 samples and asymmetric cases include *n* = 30 samples (5 seeds). Reported quantities include Spearman’s rank correlation coefficient ρ (significance always <0.001 except in symmetric models, where p≈0.15 in the baseline scenario and p≈0.005 otherwise), the pediatric–adult discrimination AUC, the corresponding effect size *r*, and group-wise medians.(PDF)

S13 TableStatistics comparing coverage and branch-resolved homogeneity across scenarios.For each scenario, symmetric cases include *n* = 6 samples and asymmetric cases include *n* = 30 samples (5 seeds). Reported quantities include Spearman’s rank correlation coefficient ρ (significance always <0.001 except in symmetric models, where p≈0.15 in the baseline scenario and p≈0.005 otherwise), the pediatric–adult discrimination AUC, the corresponding effect size *r*, and group-wise medians.(PDF)

S1 Algorithm Computation of local gravitational parameters. (PDF)

S2 Algorithm Single-step DRL workflow for SRT protocol optimization. The policy update UpdatePBO is detailed in S3 Algorithm. (PDF)

S3 Algorithm Policy-Based Optimization (PBO) update (UpdatePBO in S2 Algorithm). (PDF)
