# Supplementary material for: Reassessing adult surfactant replacement therapy with mechanics-informed reinforcement learning
Source: PLoS Comput Biol. 2026 Aug 18;22(8):e1014629. doi: 10.1371/journal.pcbi.1014629 (PMC13485052; doi:10.1371/journal.pcbi.1014629)
Supplement: Supplementary file 1 [file pcbi.1014629.s001.pdf]

Supporting Information

for

Reassessing adult surfactant replacement therapy with mechanics-informed reinforcement learning

Philippe Meliga, Gregor Roncin, Alejandro Yepes Peñaranda, Elie Hachem

1 Asymmetric airway tree generation

This section describes the stochastic scheme used to generate asymmetric airway trees from the baseline scaling law introduced in Section Geometry generation and structural parameters. We detail the sampling of the perturbed division ratio and provide additional statistics and visualizations illustrating the resulting seed-to-seed morphological variability in the pediatric and adult models considered in this study.

1.1 Stochastic sampling of asymmetric division ratios

At each bifurcation, daughter airway diameters are computed as $a_1 = r^{1/3}a_0$ and $a_2 = (1-r)^{1/3}a_0$ using the following two-step sampling procedure: first, an asymmetry coefficient is drawn from a Gaussian distribution

$$\varepsilon \sim \mathcal{N}(s\mu, \sigma^2), \qquad s \in \{-1, +1\}, \tag{S1}$$

where the random sign s is drawn uniformly from $\{-1, +1\}$. After clipping to $\varepsilon \in [-0.5, 0.5]$ (to prevent near-degenerate splits in r), the values are mapped to the effective division ratio as

$$r = \frac{1+\varepsilon}{2}\,. \tag{S2}$$

This formulation yields a double-lobed distribution with peaks near $0.5 \pm \mu/2$, promoting architectural asymmetry. The standard deviation σ controls the spread of each lobe: larger values increase deviations from the peaks but also raise the likelihood of near-symmetric branches. The resulting diameters $a_{1,2}(i)$ are thus defined locally at each bifurcation from its sampled division ratio.

In this work, we set the ratio $\mu/\sigma = 3$ as a practical compromise between variability and regularity of the resulting branching patterns, limiting the accumulation of quasi-symmetric values while still preserving a small fraction of them. Each individual branch receives a unique value, following a breadth-first traversal of the tree structure to ensure consistent labeling. Deterministic tree generation is enforced by initializing the pseudo-random number generator with a fixed seed, so that repeated runs with the same seed yield identical airway trees (independent of parallel execution). Characteristic pediatric and adult histograms of branch count versus normalized asymmetry coefficient are shown in S1 Fig for three representative random seeds. Variability across seeds is largely driven by sampling noise and decreases with the number of bifurcations. Thus, for adult trees (15 generations), division-ratio histograms are only weakly sensitive to the exact value of μ provided that the lobes remain well separated (i.e., $\mu/\sigma \gtrsim 3$–4), and Eq (S1) yields a stable double-lobed distribution with minimal seed-to-seed variability. In contrast, pediatric trees (8 generations) involve fewer bifurcations, so the corresponding histograms appear more scattered and more seed-dependent.

1.2 Additional visualizations

The supplementary airway-tree realizations shown in S2 Fig and S3 Fig complement Fig 3 in the main text by showing the five stochastic airway-tree realizations used for the mildly ($\mu = 0.075$) and highly ($\mu = 0.15$) asymmetric cases listed in Table 1. Trees with the same ID correspond to the same seed, with the pediatric realization matching the first eight generations of the associated adult tree. These realizations are used throughout this study as a controlled sensitivity axis to probe anatomical asymmetry and seed-to-seed variability within the present synthetic framework, rather than to represent population-level anatomical diversity.

2 Surfactant-transport model: governing equations and recursion details

This section summarizes the governing equations of the surfactant-transport (SRT) model used in the simulator. The bifurcation-scale closure follows the plug-transport framework of Zheng *et al.* [1, 2], later applied recursively at organ scale by Filoche *et al.* [3]. We report the coating law, the pressure-balance formulation used to compute splitting, the nonlinear friction model introduced in the present work, and the procedure for recomputing the local pitch and roll angles at each bifurcation. Additional closed-form expressions for the splitting coefficients are given in Section 2.5.

2.1 Dimensionless groups and local state variables

The parent plug state at a bifurcation is defined by its incoming volume V_0 and flow rate Q_0. The relevant dimensionless groups are expressed in terms of Q_0 and the parent airway diameter a_0:

$$\mathrm{Ca}_p = \frac{4\mu Q_0}{\sigma \pi a_0^2}, \qquad \mathrm{Re}_p = \frac{4\rho Q_0}{\mu \pi a_0}, \qquad \mathrm{Bo} = \frac{\rho g a_0^2}{\sigma}, \tag{S3}$$

where μ is viscosity, ρ density, σ surface tension, and g gravity. Daughter branches are indexed by $i \in \{1, 2\}$ and characterized by diameters a_i, branching angles θ_i, and local gravitational orientation (γ, ϕ).

2.2 Deposition and plug splitting in a single bifurcation

Coating losses are modeled as described in Section Surfactant transport model. As the plug travels along the parent airway, it deposits a trailing film on the wall, reducing the volume available for splitting at the next bifurcation. The resulting post-coating volume is

$$\tilde{V}_0 = V_0 \left(1 - 0.36\left(1 - \mathrm{e}^{-2\,\mathrm{Ca}_p^{0.523}}\right)\right)^2. \tag{S4}$$

At the bifurcation, this remaining volume is split instantaneously according to a splitting factor $\Lambda \in [0, 1]$:

$$V_1 = \Lambda \tilde{V}_0, \qquad V_2 = (1 - \Lambda)\tilde{V}_0\,. \tag{S5}$$

Thus, $\Lambda = 0$ sends all liquid to daughter 2, whereas $\Lambda = 1$ sends all liquid to daughter 1. Following [2], Λ is determined from a local pressure balance at the junction by imposing equal downstream pressures; $P_1 = P_2$.

For each daughter branch, the pressure drop from the parent branch to the downstream end is written as the sum of meniscus pressure jumps and distributed hydrostatic, inertial, and viscous

friction losses. The viscous contribution is modeled using the nonlinear-plus-linear law proposed by [2]:

$$\Delta P_{\mu,i} = 32\alpha \frac{(\rho\mu V_i)^{1/2}}{\pi^2 a_i^5} Q_i^{3/2} + 512\beta \frac{\mu V_i}{\pi^2 a_i^6} Q_i\,, \qquad i \in \{1,2\}\,, \tag{S6}$$

where V_i and Q_i denote the post-split plug volume and flow rate in daughter i, and α and β are friction factors. The model combines a linear contribution (second term) with a nonlinear correction documented in bifurcation flow models [4]. Setting $\alpha = 0$ and $\beta = 1$ recovers the standard Poiseuille law commonly assumed in organ-scale implementations [3, 5].

Enforcing $P_1 = P_2$ yields a quadratic equation for Λ:

$$A\Lambda^2 + B\Lambda + C = 0, \tag{S7}$$

with coefficients A, B, C depending on the local geometry $(a_0, a_1, a_2, \theta_1, \theta_2)$, gravitational orientation (γ, ϕ), and the dimensionless groups $(\mathrm{Ca}_p, \mathrm{Re}_p, \mathrm{Bo})$ (see Section 2.5 for exhaustive closed-form expressions). In practice, we evaluate (A, B, C) and solve (S7) for Λ.

2.3 Nonlinear friction closure and calibration

The models in [1, 2] were developed for distinct capillary- and inertia-dominated parameter ranges, and the authors noted that a single-regime theory may lead to systematic under- or over-predictions outside its range of validity. For recursive tree-level simulations, we favor a single globally calibrated friction closure over an explicit regime-switching interpolation, in order to avoid introducing additional sensitivity to the precise location and sharpness of such a transition across neighboring bifurcations. We therefore use a single nonlinear-plus-linear friction law with constant coefficients (α, β), calibrated globally against the combined experimental dataset spanning both regimes. This choice may yield a slightly less accurate asymptotic fit within each limiting regime, but provides a more robust organ-scale closure. Although Zheng [2] also reported an inclination-angle dependence of the friction law, we neglect it here because the available angular data span too limited a range for reliable extrapolation. Posture effects are therefore included only through the hydrostatic terms of the pressure balance.

A least-squares calibration is performed over $N = 131$ experimental values digitized from [1, 2], spanning four fluids and wide parameter ranges ($\mathrm{Ca}_p \in [5 \times 10^{-5},\, 10^{-1}]$, $\mathrm{Re}_p \in [2 \times 10^{-3},\, 600]$; see S1 Table). This yields the friction parameters

$$\alpha = 7.86, \qquad \beta = 10.57\,, \tag{S8}$$

with mean squared error $\mathrm{MSE} = 1.1 \times 10^{-2}$ computed between model predictions and the corresponding experimental values.

2.4 Tree-level recursion and posture mapping

To simulate transport over the full airway tree, the above bifurcation map is applied recursively from the trachea to terminal branches. At each bifurcation, the local gravitational parameters (γ, ϕ) are computed from the 3D orientation of the parent/daughter directions and the global body posture. The pseudocode used to compute local pitch/roll from airway direction vectors is provided in S1 Algorithm, ensuring a consistent propagation of posture throughout the tree.

2.5 Closed-form splitting coefficients

We define

$$\Delta_k(r) = r^{-k/3} - (1-r)^{-k/3}\,, \tag{S9}$$

where r is the diameter-division ratio defining the daughter diameters relative to the parent. The coefficients (A, B, C) entering Eq (S7) read

$$A = \frac{1}{2}\Delta_4(r)\mathrm{CaRe} + \alpha\Delta_5(r)\mathrm{Ca}(\mathrm{Re}\tilde{V}_0)^{1/2} + 8\beta\Delta_6(r)\mathrm{Ca}\tilde{V}_0\,, \tag{S10}$$

$$B = \frac{1}{(1-r)^{4/3}}\mathrm{CaRe} + \left(\frac{f_1}{r^{2/3}} + \frac{f_2}{(1-r)^{2/3}}\right)\mathrm{Bo}\tilde{V}_0 + \frac{2\alpha}{(1-r)^{5/3}}\mathrm{Ca}(\mathrm{Re}\tilde{V}_0)^{1/2} + \frac{16\beta}{(1-r)^2}\mathrm{Ca}\tilde{V}_0\,, \tag{S11}$$

$$C = -\frac{1}{2(1-r)^{4/3}}\mathrm{CaRe} - \frac{f_2}{(1-r)^{2/3}}\mathrm{Bo}\tilde{V}_0 - \frac{\alpha}{(1-r)^{5/3}}\mathrm{Ca}(\mathrm{Re}\tilde{V}_0)^{1/2} - \frac{8\beta}{(1-r)^2}\mathrm{Ca}\tilde{V}_0 - 2\Delta_1(r)\,. \tag{S12}$$

Here, $\tilde{V}_0$ is the post-coating volume (Eq (S4)), $f_i = \sin\theta_i \sin\phi - \cos\theta_i \sin\gamma$ are gravity factors, and we use the modified capillary and Reynolds numbers

$$\mathrm{Ca} = \left(1 - \frac{2h}{a_0}\right)^2 \mathrm{Ca}_p, \qquad \mathrm{Re} = \left(1 - \frac{2h}{a_0}\right)^2 \mathrm{Re}_p\,. \tag{S13}$$

3 Deep reinforcement learning framework and implementation

This section provides complementary methodological details on the reinforcement-learning ingredients used in the main text. We first recall the standard RL/DRL concepts required to frame our approach, then summarize the specific rationale of the single-step formulation adopted for SRT protocol optimization. We finally report implementation details for the in-house policy-based optimization (PBO) algorithm and for the action-to-protocol mapping, including practical choices related to aliquot handling, exploration, and training schedules.

3.1 Background on standard RL and DRL

For completeness, we briefly recall standard RL terminology used in the main text. At each time step, the agent observes a state s_t, selects an action a_t, receives a reward r_t, and transitions to a new state s_{t+1}. The goal is to optimize the expected return over an episode, defined as a sequence from an initial to a terminal state.

In DRL, policies are parameterized using DNNs, typically relying on fully connected feedforward architectures. Neurons are organized in layers, each computing a weighted sum of inputs followed by a nonlinear activation. Learning consists of iteratively updating the network's weights and biases via backpropagation to minimize a loss function that encodes the discrepancy between predicted and target action values, thereby improving policy performance over time.

3.2 Single-step DRL background

In contrast to sequential RL, the present formulation consists of a single state–action evaluation followed by a terminal reward: in this open-loop setting, the policy outputs a complete protocol in one shot, and the reward is evaluated only at the end of the simulated administration (i.e., no inter-aliquot observations or feedback). The learning problem therefore reduces to identifying an optimal open-loop action conditioned on the airway description and the selected optimization scenario. From a methodological standpoint, this can be viewed as stochastic black-box optimization with a learned action-sampling distribution, which is well suited to problems where intermediate observations are unavailable or not exploited.

This perspective also rationalizes the use of compact policy parametrizations in low-dimensional settings. Because the agent learns a static mapping rather than a temporal decision rule, compact

fully connected architectures are typically sufficient to represent high-performing policies in the present SRT setting (hence the small hidden-layer sizes; see Section 3.3). The single-step approach therefore provides a natural way to optimize complete multi-aliquot protocols in one shot while preserving a consistent DRL training pipeline across scenarios.

3.3 Policy-Based Optimization: additional implementation details

We provide practical implementation details of the PBO algorithm, with emphasis on action distribution parametrization, training setup, and loss-stabilizing heuristics. The algorithm used in this work is the one introduced and validated in [6] and reused in our prior fluid-mechanics applications [7, 8]. Unless stated otherwise, we keep the same default settings—policy parameterization, covariance construction, reward normalization/clipping, history reuse—and only adapt the action-to-protocol mapping and reward definition to the present SRT setting.

Policy objective and single-step setting. In the present single-step formulation, each episode consists of a single state–action evaluation followed by a terminal reward: the policy outputs a complete protocol in one shot (open-loop), and the reward is evaluated only at the end of the simulated administration (i.e., no inter-aliquot observations or feedback). Policy parameters are updated using the PBO loss (Eq (6) in the main text), which reduces to a one-step policy-gradient objective in this setting. For clarity, quantization (rounding mapped continuous protocol parameters to predefined step sizes) is applied inside the training loop prior to each simulator call, and policy updates use rewards computed on the resulting quantized protocols. Because PBO uses a score-function (log-likelihood) policy-gradient update rather than backpropagating through the environment, the simulator and action-to-protocol mapping need not be differentiable.

Network parametrization. PBO represents the action-sampling policy as a d-dimensional multivariate normal distribution $\pi_\theta(a) = \mathcal{N}(\mu, \Sigma)$. The mean μ, standard deviations σ, and correlation parameters ρ are predicted by three independent fully connected networks with potentially distinct architectural and training choices.

Covariance parameterization. The policy outputs $\mu \in [-1,1]^d$ (tanh) and $\sigma \in [0,1]^d$, $\rho \in [0,1]^D$ (sigmoid), with $D = d(d-1)/2$. Following hypersphere decomposition [9, 10], ρ is mapped to a lower-triangular matrix L with unit diagonal (off-diagonal entries are products of sine/cosine terms, hence in $[-1,1]$), and the correlation matrix is obtained as $C = LL^\top$ (positive semidefinite by construction). The covariance is then assembled as $\Sigma = \mathrm{diag}(\sigma)\, C\, \mathrm{diag}(\sigma)$, preserving the interpretation of σ as per-dimension standard deviations.

History reuse (decayed experience). To stabilize learning of a full covariance matrix in higher-dimensional action spaces, PBO reuses a short history of previously evaluated (a, r) pairs with an exponential decay [6], $\eta = 1 - \exp(-\alpha d)$, with $\alpha = 0.35$. This yields $\eta \approx 0.50$ for the Baseline scenario ($d = 2$) and rapidly approaches 1 for the higher-dimensional scenarios ($d = 2n_{\mathrm{ctrl}} + 1$ and above). This mirrors a common CMA-ES heuristic, where covariance information from past generations is reused with exponentially decayed weights [11].

Reward normalization and filtering. Prior to the policy update, rewards are whitened within each batch (zero mean, unit variance) to improve numerical conditioning and reduce gradient variance [6]. The loss retains only above-mean whitened rewards through the $\max(\tilde{r}, 0)$ factor. In a one-step policy-gradient update, this amounts to updating the policy using only samples that outperform the batch mean, which (i) emphasizes improving directions in the sampled batch and (ii) avoids negative weights that can amplify noisy gradients when exploration is broad (early training). This heuristic is in the same spirit as elite selection used in CMA-ES–type updates [11].

Hyperparameters. Each of the three neural networks (mean, variance, correlation) is updated for n_e epochs using the Adam optimizer with learning rate λ_r. For each update, training data are drawn from a history of n_h episodes, shuffled, and split into n_b mini-batches (with mini-batch sizes chosen as multiples of the number of parallel environments n_{env}). The main PBO meta-parameters and network sizes are summarized in S2 Table. Consistent with the open-loop, stateless formulation, compact fully connected networks are sufficient here because the policy learns a direct parameterization of a distribution over complete protocols rather than a state-dependent control law.

Training verification. Training stability was monitored by logging the reward components and action traces; representative learning trajectories (reward and sub-scores) are reported in Section 7.1, showing smooth convergence over the training horizon.

Workflow The end-to-end RL workflow is summarized in S2 Algorithm, with the corresponding PBO policy update detailed in S3 Algorithm. We emphasize that the optimization is open-loop: each episode evaluates a complete instillation protocol and yields a terminal reward for a single policy update, with no inter-aliquot observations.

3.4 Action-to-protocol mapping: implementation details

This subsection details the practical design choices underlying the action-to-protocol mapping, including variable instillation-count strategies and the exploration-to-exploitation schedule used in the single-step setting.

Aliquot-count handling. The spline-based parametrization makes it possible, in principle, to vary the number of aliquots n_{alqt} without changing the action vector dimensionality, which depends only on the number of control points. In practice, this flexibility led to suboptimal exploration of effective configurations. To mitigate this limitation, a conditional warm-up phase imposed a fixed number of instillations for a set of episodes, providing more informative gradients before allowing free selection. Despite this, independent runs with a fixed aliquot count proved more efficient. Accordingly, in the remainder of this work, the number of aliquots varies across experiments, but each individual optimization uses a fixed instillation count.

Training schedule and seed selection. For each scenario, three independent training runs are performed with different random seeds, each comprising 300 episodes. This corresponds to approximately 2–6 h of total CPU time per run, depending on the number of aliquots. We report the best-performing run among three seeds as a pragmatic estimate of what is achievable under a fixed training budget in this setting, rather than as a robustness statistic. This choice reflects the non-convex reward landscape, in which different seeds can converge to distinct yet high-quality local optima. Such policy multiplicity is expected given partial redundancy among controls (dose allocation, flow modulation, posture), whereby different temporal combinations (e.g., the aliquot instillation sequence) can yield similar distal coverage patterns. An exhaustive quantification over all airway realizations, scenarios, and aliquot counts would require a substantially larger training campaign. The present study instead reports a targeted run-to-run analysis in Section 6, focusing on score-level recoverability and policy-level multiplicity in representative highly asymmetric settings.

4 Reward parameters

The reward used in the main text combines four interpretable components:

$$r = S_c \, S_e \, S_u \, S_v \, , \tag{S14}$$

where $S_c \in [0, 100]$ is the branch-level coverage score, and S_e, S_u, and S_v are bounded modulation factors in $[0, 1]$. As a result, the overall reward naturally follows the 0–100 scale of the coverage term.

A summary of the numerical parameters used in the definition of the reward components is provided in S3 Table. Their role is to specify the branch-wise tolerance bands used for coverage and conditional uniformity, and the shape of the local and global overdosing penalties.

Coverage score. The coverage score is the main branch-level driver of the reward. By construction, $S_c = 100$ corresponds to the case where all terminal branches lie within the full-credit target range, whereas low values indicate that a substantial fraction of branches remain either clearly below target or strongly overdosed. Because the score is averaged over all terminal branches, it can be interpreted as a tolerance-based proxy for branch-level distal coverage. The main experiments use $x_{\text{min}} = 0.3$, $x_{\text{max}} = 1.4$, and $k_{\text{over}} = 3.0$. Under this parametrization, terminal branches receiving less than 30 % of the prescribed local target receive no credit, the local score increases linearly up to the target, remains maximal up to 140 % of target, and then decays exponentially beyond that range (S4 Fig, panel a). This choice reflects a compromise between rewarding broad branch-level distal reach and preventing trivial gains through strong localized overdosing.

Efficiency score. The efficiency score quantifies the fraction of the total instilled volume that reaches the terminal tree. A value close to 1 indicates that most of the instilled material reaches distal branches, whereas lower values indicate substantial proximal losses. Since S_e is used multiplicatively, it mainly serves to suppress solutions with broad but weak distal delivery.

Conditional uniformity score. The conditional uniformity score measures how uniformly the adequately served branches are matched relative to one another. It is evaluated only on the subset of terminal branches whose branch-wise ratio lies within the admissible interval $(x_{\text{u,min}}, x_{\text{u,max}})$, with values close to 1 indicating low dispersion within this subset. The main experiments use $(x_{\text{u,min}}, x_{\text{u,max}}) = (0.75, 1.25)$, an interval chosen to be narrower than the broader coverage interval, since its role is not to assess all branches receiving nonzero credit, but to quantify the relative uniformity of the subset of branches already lying in a reasonably acceptable neighborhood of the target volume.

Volume penalty. The volume factor leaves solutions at or below the prescribed target volume unchanged and progressively downweights solutions that rely on excess total instilled volume. By construction, the penalty decreases smoothly between $V_{\text{inst}} = V_{\text{target}}$ and $V_{\text{inst}} = 2V_{\text{target}}$. In the main experiments, $K_{\text{over}} = 1.5$, corresponding to a moderate penalty shape that only mildly penalizes small excesses above the prescribed target (S4 Fig, panel b).

In the main experiments, the distal target volume entering the coverage score is set to a fixed fraction f_d of the prescribed target volume,

$$V_{\text{target,d}} = f_d \, V_{\text{target}} \, , \tag{S15}$$

across all scenarios. This choice provides a uniform normalization of the branch-level target across cases, so that a given coverage score retains the same interpretation from one scenario to another. It was chosen as a pragmatic compromise. Choosing too small a distal target would implicitly accept that a large fraction of the instilled dose may be lost to coating and does not contribute to distal delivery. Conversely, choosing too large a distal target would make the score excessively stringent and render most mechanically reasonable solutions poorly rated. The retained value $f_d = 0.75$ was informed by preliminary runs, in which distal efficiency typically lay between 0.7 and 0.9, and most often between 0.7 and 0.8.

5 Protocol parameter ranges rationale

This section provides complementary justification for the numerical ranges used in the optimization experiments and clarifies how protocol parameters are scaled across pediatric and adult models.

The lower end of the total instilled volumes aligns with common pediatric protocols (1–4 mL/kg). The upper bound, while excessive for neonates, is scaled for adults to reach plausible delivery regimes relevant to larger airway surfaces, consistent with previous adult trials [12]. Flow rates correspond to ventilator airflow interacting with the surfactant, not to the instillation rate itself, which is much lower in practice to avoid reflux, barotrauma, and other complications. The explored range (1–7 mL/kg/s) is chosen as an order-of-magnitude, model-consistent range: it spans values below typical neonatal respiratory flow rates (10–15 mL/kg/s) and overlaps reported adult breathing ranges (4–6 mL/kg/s).

In this formulation, the flow rate is used as a proxy for the ventilatory airflow driving bolus transport rather than for the mechanical instillation rate, which is intentionally kept much lower in clinical practice. Although the resulting absolute rates may appear high, they remain within normalized ranges (mL/kg/s) used in prior modeling studies of surfactant transport [3, 5, 13]. This abstraction provides a consistent normalization for exploring protocol sensitivity within each age group. Cross-scale comparisons should therefore be interpreted as qualitative, mechanism-driven trends rather than as one-to-one clinical equivalents. Overall, these ranges define a coherent, normalized parameterization for comparing optimization outcomes across the pediatric and adult cases considered here.

6 Reward sensitivity analysis

This section assesses robustness of the learned protocols to reward-design choices by repeating the full training pipeline under systematically modified reward settings.

We consider three groups of reward variants: (i) branch-level coverage variants, which modify the lower and upper coverage thresholds together with the local overdosing penalty; (ii) conditional uniformity variants, which modify the admissible ratio window used to compute the conditional uniformity score; and (iii) distal-dose variants, which modify the distal target fraction f_d entering the definition of $V_{\mathrm{target,d}}$. This analysis is conducted on a representative highly asymmetric pediatric airway tree, using 2 aliquots and a prescribed target volume $V_{\mathrm{target}} = 2\,\mathrm{mL}$. This provides a challenging stress test while avoiding a near-ceiling regime where uniformly high scores would reduce the diagnostic value of the sensitivity tests. Variant definitions are summarized in S4 Table. We did not perform a dedicated sensitivity sweep of the global volume penalty term, because changing this term would directly alter the trade-off between excess total volume and distal delivery, thereby redefining the optimization problem rather than perturbing the branch-level scoring rules. The present analysis therefore focuses on reward-parameter groups that preserve the same overall volume constraint while modifying only the branch-level scoring structure. Consistently, the volume factor remained extremely close to unity for all converged solutions retained in the main analysis ($S_v > 0.999$), indicating a practically secondary influence relative to the branch-level terms. Accordingly, S_v is omitted from the summary tables below.

Experimental setup and reporting. Sensitivity runs follow the same training pipeline as the reference workflow, with 3 independent training runs for each scenario and reward setting. Within each run, exponential moving averages (EMA) of the reward components are computed over a 10-episode window, and the reported terminal values correspond to the mean of the last ten EMA samples. For the main-text results, the reported protocol corresponds to the best-performing policy among the 3 runs, interpreted as a pragmatic upper bound under a fixed training budget. The present sensitivity analysis instead focuses on two complementary aspects: the stability of the attained physical scores, and the multiplicity/alignment of the corresponding learned policies across runs and reward variants.

Accordingly, we report two complementary summaries, both expressed as median [IQR]. In the reference setting, final reward and score components are summarized across the 3 runs, with policy alignment quantified from the 3 unique pairwise cosine similarities between learned policies. For reward variants, we compare the ensemble of 3 policies learned under the modified reward with the ensemble learned under the reference reward. Relative score changes are therefore based on the 9 variant-to-reference pairwise comparisons. For policy alignment, loose and tight variants are pooled within each family, so each family-level summary is based on 18 cosine similarities against the reference ensemble. These cross-ensemble summaries capture both the shift of the learned solution set relative to the reference reward and the residual multiplicity of near-equivalent optima within the two run ensembles.

In all cases, cosine alignment is computed in normalized action space after linearly mapping each action component to $[-1, 1]$ using its admissible range. Depending on the scenario, we additionally report reduced-space cosine alignments obtained after excluding posture variables, or excluding both posture and rheological variables, in order to distinguish core policy structure from variability carried by higher-dimensional compensatory controls.

Reference reward: policy multiplicity across scenarios. As shown in S5 Table, under the reference reward, the simplest baseline scenario yields a practically unique solution across runs, with identical scores and perfect policy alignment. Introducing per-dose control already removes strict uniqueness, but the learned policies remain closely aligned and the attained scores remain indistinguishable within the reported dispersion. Thus, the additional control freedom already allows multiple near-equivalent solutions, but these remain geometrically close in action space and have negligible impact on the attained physical outcome.

A sharper transition appears once posture is introduced: full-policy alignment drops markedly and becomes highly dispersed, whereas the final scores remain tightly clustered across runs. This indicates that several distinct high-performing optima coexist while yielding essentially the same reward and physical metrics. When posture variables are excluded, both the median alignment and its dispersion improve substantially, showing that most of the visible policy multiplicity is carried by posture scheduling rather than by the underlying non-postural structure of the solution.

Once rheology optimization is enabled, the full-policy misalignment remains substantial, but again decreases markedly once posture variables are excluded. Additional tests excluding both posture and rheological variables did not reveal systematically tighter clustering than the posture-excluded comparison alone. This suggests that rheological controls do not simply add variability on top of posture, but may also allow alternative compensatory non-postural structures. This behavior is also consistent with the outcome-oriented nature of the reward: the metric is designed to identify high-quality delivery patterns rather than a unique control realization, so multiple protocols may legitimately achieve similar final scores. Overall, these results support three main conclusions: when the control space is small, the learned solution is practically unique; enriching the control space increases the non-uniqueness of high-performing solutions; and most of the resulting variability is carried by posture-related degrees of freedom, with only limited impact on the attained reward and physical metrics.

Sensitivity to reward variants. The same general picture holds under the considered reward-design perturbations, as summarized in S6 Table. As expected, the largest score changes are produced by the coverage variants, with looser thresholds increasing both S_c and the reward, and tighter thresholds decreasing them by comparable amounts. This is expected because these variants directly modify the local branch-level scoring rule; absolute values of S_c and r are therefore not directly comparable across variants in the same way as invariant physical metrics. By contrast, the efficiency and conditional uniformity scores remain only weakly affected, typically within a few percent, indicating that the underlying mechanical outcomes remain globally stable. Variants acting on the conditional uniformity window or distal target fraction induce only modest score changes compared with the coverage family.

Despite these score shifts, the associated policy changes remain strongly structured. Cosine alignment on the full control vector is only moderate, especially for the coverage and distal-dose families, which is consistent with the fact that these variants alter how favorable delivery trade-offs are scored and can therefore redirect the optimization toward different but still high-performing control realizations. Most importantly, alignment rises substantially once posture variables are excluded. Thus, reward-design perturbations mainly affect which near-equivalent posture schedules are selected, rather than the core non-postural structure of the learned solution. Together with the weak variation of S_e and S_u (and the fact that S_v remains effectively unchanged), this indicates that the present reward formulation is robust in the sense most relevant here: moderate changes in its parametrization do not materially alter the attained physical outcome, even though they may select different members of a broader family of high-performing policies.

Although S6 Table focuses on the pediatric per-dose + posture scenario, the same analysis was repeated across all pediatric scenarios and led to the same qualitative conclusions; the full numerical results are provided in the supplementary data repository. In particular, the hierarchy already seen under the reference reward was preserved: the baseline setting remained practically unique, whereas policy multiplicity increased progressively as additional control levers were introduced, while preserving the same notion of robustness emphasized here, namely weak sensitivity of the attained physical outcome and high alignment ($\gtrsim 0.94$) once posture variables were excluded.

Overall, these sensitivity analyses indicate that moderate changes in reward design mainly affect which near-equivalent policy is selected—especially through posture-related degrees of freedom—while leaving the main physical conclusions and score hierarchy essentially unchanged.

Adult counterpoint. The corresponding results for a representative highly asymmetric adult airway model are reported in S7 Table–S8 Table, using the adult-scaled version of the same underlying asymmetric tree as in the pediatric analysis, with 2 aliquots and a prescribed target volume $V_{\text{target}} = 280\,\text{mL}$. Under the reference reward, the full policy vector remains non-unique across runs, with only moderate alignment on all controls. However, once posture variables are excluded, alignment becomes nearly perfect (0.996), while the attained scores remain tightly clustered. Compared with the pediatric posture-enabled scenario, this indicates a much more constrained non-postural control space in the adult case: distinct high-performing solutions still exist, but their variability is carried almost entirely by posture rather than by alternative volume/flow realizations.

The same conclusion holds under the tested reward variants. Coverage variants induce the largest changes in S_c and reward, as expected from the modified branch-level scoring rule, whereas the other score components remain only weakly affected. Yet even when the full-vector cosine alignment is only moderate, alignment without posture remains essentially unchanged and close to unity for all variant families ($\gtrsim 0.99$). Thus, in the adult case, reward perturbations mainly modify which posture schedule is selected, while leaving the non-postural structure of the learned solution almost unchanged. This reinforces the interpretation suggested by the pediatric analysis: policy multiplicity should not be read as instability of the attained physical outcome, and its non-postural component becomes increasingly constrained as the delivery problem becomes more mechanically restrictive.

7 Additional optimization results and diagnostics

This section provides supporting results that complement the main-text comparisons. We document representative training dynamics to confirm convergence and stability, then report additional global summaries and branch-level diagnostics for both the mechanics-only and rheology-extended control spaces. Trajectories display both raw episode-wise values and their corresponding exponential moving averages (EMA), computed over a 10-episode window. Terminal values reported in the tables are defined as the mean of the last ten EMA values.

7.1 Convergence behavior and training stability

To ensure that reported results reflect stable and meaningful policies, we briefly assess training dynamics using representative runs in both pediatric and adult settings for the Per-dose + Posture scenario with four instillations (S5 Fig–S6 Fig). In both cases, the total reward and its components converge smoothly within about 200 episodes, indicating coherent progress across all objectives. Total instilled volume stabilizes early, minimizing the global overvolume penalty, while individual volume fractions continue to adjust until mid-training, refining how the dose is distributed across instillations. Flow rates and posture parameters follow a similar convergence timeline, although the latter remain visually more irregular because posture is discretized on a 15° grid, so apparent jumps often correspond to local exploration among neighboring admissible orientations rather than to qualitatively different strategies. In some more fractionated adult runs (4 or 8 aliquots), small late-stage fluctuations can also persist for individual aliquot-specific action parameters, particularly flow-rate components. These do not affect convergence of the protocol-level reward or delivery metrics and likely reflect partial non-identifiability among near-equivalent solutions once the dominant control degrees of freedom have stabilized. Similar patterns were observed across all tested geometries and settings, as monitored through systematic logging of action values, decomposed reward components, and their corresponding EMA trajectories.

7.2 Baseline performance

7.2.1 Symmetric models

Beyond the performance metrics summarized in the main text, S7 Fig, panel a shows that conditional uniformity is already high in both pediatric and adult models and varies much less than coverage or efficiency. This indicates that, in symmetric trees, the main challenge is not to homogenize already well-served branches, but first to deliver enough material distally. In adults, the fraction of branches entering the conditional uniformity calculation increases monotonically with volume, consistent with a progressive transition from underdosed to well-served distal regions; see S7 Fig, panel b. In the pediatric model, this fraction may instead decrease slightly at high volume even while coverage approaches its ceiling. This does not indicate a degradation of overall delivery quality; rather, it reflects the conditional nature of S_u: as volume increases, some branches move outside the stricter admissible ratio window used to define this metric while still remaining within the broader full-credit range of the coverage score.

7.2.2 Mildly asymmetric models

Mild asymmetry confirms that the degradation induced by geometric imbalance is both progressive and scale-dependent. As shown in S9 Table and S8 Fig, pediatric performance remains high and close to the symmetric reference case across the explored target volumes, with only limited dispersion across airway realizations. Thus, in the pediatric model, mild asymmetry only weakly perturbs the favorable baseline regime identified in the main text. By contrast, the adult model already shows a clear loss of performance relative to the symmetric case, especially at low volume. Mild asymmetry therefore already pushes the adult baseline protocol out of the near-ceiling regime, even though the resulting degradation remains substantially weaker than in the highly asymmetric models.

The corresponding efficiency and volume penalty curves remain qualitatively similar to those discussed in the main text and in the highly asymmetric case. In particular, pediatric efficiency remains systematically higher than adult efficiency, and both scales still show monotonic improvement with prescribed volume and a modest benefit from fractionation. The main effect of mild asymmetry is therefore seen primarily in the branch-level coverage term, rather than in a qualitative restructuring of the global auxiliary metrics.

This interpretation is reinforced by the conditional uniformity analysis shown in S9 Fig. The conditional uniformity score S_u remains high and nearly flat across target volumes and aliquot

numbers in both pediatric and adult models, indicating that the relative homogeneity of already well-served terminal branches is largely preserved. The main asymmetry effect is instead visible in the fraction of terminal branches entering the conditional uniformity calculation. This fraction remains relatively high in the mildly asymmetric pediatric model, but is substantially lower in the adult model. Therefore, even in the mild asymmetry regime, the main loss mechanism is not a collapse of uniformity within the retained branch population, but a reduction in the number of branches that reach this sufficiently well-dosed regime in the first place.

Overall, the mildly asymmetric results support the same hierarchy as the highly asymmetric case, but in attenuated form: pediatric delivery remains close to the symmetric baseline, whereas adult delivery already departs substantially from it, showing that the asymmetry-induced collapse is progressive rather than binary and becomes increasingly severe with both geometric imbalance and scale.

7.2.3 Highly asymmetric models

The interpretation of the highly asymmetric baseline results reported in the main text is refined in S10 Fig. Despite the strong degradation in coverage, the conditional uniformity score S_u remains relatively high and varies only weakly with prescribed volume and number of aliquots. This indicates that, among the terminal branches that still fall within the admissible ratio window used to define S_u, relative homogeneity is not strongly degraded by asymmetry. The main asymmetry effect is instead seen in the fraction of terminal branches entering the conditional uniformity calculation, which drops sharply relative to the symmetric case, especially in the adult model. Thus, under strong asymmetry, the dominant loss mechanism is not a collapse of uniformity among the branches retained in S_u, but a marked reduction in their number: many branches no longer reach the sufficiently well-dosed regime required to contribute to this metric.

7.3 Effect of scenario complexity

7.3.1 Symmetric models

The main-text analysis of staged scenario complexity in the reference symmetric airway models is further detailed in S11 Fig, which shows the conditional uniformity score S_u and the fraction of terminal branches retained in its calculation.

Across all scenarios, the conditional uniformity score itself remains high and varies only moderately. In the adult model, it tends to increase slightly with scenario complexity, consistent with the fact that the additional control levers improve not only overall distal delivery, but also the uniformity of the branches that remain within the admissible ratio window used to define S_u. In the pediatric model, by contrast, S_u remains close to unity in all cases, indicating that once a branch is retained in the conditional uniformity calculation, its local dose distribution is already highly satisfactory even under the simpler scenarios.

The more discriminating quantity is therefore the fraction of branches entering the conditional uniformity calculation. In adults, this fraction generally increases with scenario complexity, especially in the lower-volume regimes, consistent with a progressive expansion of the distal branches contributing to S_u. This behavior is fully consistent with the main-text results: the more complex scenarios improve coverage not only by increasing the score of already well-covered branches, but also by bringing a larger number of branches into the regime where conditional uniformity becomes meaningful.

The pediatric model exhibits a different behavior. As scenario complexity increases, the fraction of branches entering the conditional uniformity calculation may decrease sharply at high volume, even while coverage becomes essentially perfect. This does not indicate a deterioration of delivery quality, nor an inconsistency between metrics. Rather, it reflects the more selective nature of S_u: many branches remain within the broad full-credit region of the coverage score while no longer satisfying the narrower admissible ratio window used for conditional uniformity. In other words, the apparent collapse of the retained branch fraction in the near-ceiling pediatric regime does not mean

that these branches become poorly served; it means only that they move away from the stricter "strictly well-matched" subset used to compute S_u. In this regime, conditional uniformity therefore becomes less discriminative than coverage, which is why it is reported here only as a supplementary diagnostic metric.

These results support the same interpretation as the main-text analysis: in the symmetric geometry, additional control complexity has little practical effect in the pediatric model once near-ceiling performance has been reached, whereas in the adult model it remains useful by both improving branch-level coverage and enlarging the subset of branches that can be considered uniformly well served.

7.3.2 Mildly asymmetric models

Results for the mildly asymmetric airway models are reported here using the same fixed-volume conditions as in the highly asymmetric analysis, namely 2 mL/kg in the pediatric model and 4 mL/kg in the adult model. Coverage scores are summarized in S10 Table, while S12 Fig reports the corresponding changes relative to the baseline protocol for coverage, efficiency, and total instilled volume.

The mildly asymmetric results follow the same overall hierarchy as in the highly asymmetric case, but in attenuated form. At both scales, coverage improves monotonically as optimization progresses from per-dose control to posture-enabled control and finally to rheology-enabled control, confirming that the additional control levers remain beneficial once geometric imbalance is introduced. However, the absolute gains are smaller than under strong asymmetry, consistent with the fact that mild asymmetry perturbs delivery less severely and therefore leaves less room for recovery. The pediatric model remains particularly close to ceiling, so that increased control flexibility produces only limited additional gains in final coverage. In adults, the same ordering remains visible, but the improvements remain more modest than in the highly asymmetric regime.

The efficiency and total-volume trends clarify the mechanism behind these gains. Increased control flexibility again improves the conversion of instilled material into useful distal delivery, with the largest gain obtained when rheology optimization is enabled, while posture-enabled control remains intermediate between rheology-enabled and per-dose optimization. At the same time, changes in total instilled volume remain limited: pediatric gains may be accompanied by slightly lower instilled volume, whereas in adults the optimized solutions remain close to the prescribed target volume. Thus, in the mildly asymmetric regime, richer scenarios improve performance mainly through better delivery efficiency rather than through substantial changes in the instilled volume budget.

The supplementary conditional uniformity metrics shown in S13 Fig further support this picture. At both scales, changes in the conditional uniformity score S_u remain limited, indicating that relative homogeneity among the branches retained by this metric is already good across scenarios. The clearer scenario effect is instead seen in how many branches enter the calculation, in both pediatric and adult models, consistent with a gradual expansion of the satisfactorily dosed distal set. Thus, in the mildly asymmetric regime, scenario enrichment acts mainly by bringing more branches into a satisfactorily dosed state, rather than by strongly altering the conditional uniformity within that branch population.

Overall, the mildly asymmetric results provide an intermediate regime between the symmetric and highly asymmetric cases: additional control complexity remains beneficial, the rheology-enabled scenario remains the most effective, and posture control is already more informative than in the symmetric geometry. However, because the geometric constraints are less severe than under strong asymmetry, the gains remain smaller in magnitude and the overall performance hierarchy is correspondingly less separated.

7.3.3 Highly asymmetric models

The conditional uniformity score S_u and the fraction of terminal branches entering its calculation for the staged scenario-complexity analysis in the highly asymmetric airway models are

reported in S14 Fig.

Across all scenarios, S_u itself varies comparatively little, whereas the number of branches contributing to it increases with scenario complexity. This indicates that strong asymmetry primarily limits how broadly acceptable dosing can be achieved across the distal tree, rather than strongly degrading relative homogeneity among the branches that remain in that range. The increase is visible in both pediatric and adult models, with the clearest gains observed under Rheology.

These gains nevertheless remain incomplete. Even under the most complex scenario, the selected branch fraction stays well below the levels observed in the symmetric reference geometry, especially in the adult model. This confirms that scenario enrichment mitigates but does not remove the structural limitations imposed by strong asymmetry: it expands the adequately dosed branch population, but does not restore a broadly and uniformly well-dosed distal population comparable to that of symmetric models.

8 Homogeneity metrics

This section defines the two homogeneity-based diagnostics used as companion metrics to branch-level coverage in the main text.

8.1 Global homogeneity

The global homogeneity score used in the main text is the standard homogeneity metric widely used in the literature to quantify the overall deviation of delivered volume across terminal branches.

For each terminal branch i, let

$$x'_i = \frac{v_i}{\bar{v}}, \tag{S16}$$

where v_i is the delivered volume in branch i, and $\bar{v}$ is the achieved mean branch dose. The associated relative deviation is then

$$\epsilon_i = |x'_i - 1|. \tag{S17}$$

The global homogeneity score is defined as the inverse of the root-mean-square (RMS) deviation:

$$S_\mathrm{h} = \frac{1}{\sigma_\mathrm{h}}, \qquad \sigma_\mathrm{h}^2 = \frac{1}{N}\sum_{i=1}^{N}\epsilon_i^2, \tag{S18}$$

where N denotes the total number of terminal branches.

8.2 Branch-resolved homogeneity

The branch-resolved homogeneity score is intended to evaluate spatial uniformity by rewarding the fraction of terminal branches whose local deposition remains within meaningful tolerance bands around the branch-wise mean.

For each terminal branch i, the same relative deviation $\epsilon_i = |x'_i - 1|$ is used. A local quality score $s_{\mathrm{hb},i}$ is assigned by comparing ϵ_i against a hierarchy of tolerance thresholds $\{\tau_k\}$ with associated weights w_k, ordered so that tighter tolerances receive larger weights:

$$s_{\mathrm{hb},i} = \max\{w_k \mid \epsilon_i \le \tau_k\}. \tag{S19}$$

Small deviations therefore receive high scores, whereas larger deviations are progressively penalized. The branch-level score is finally defined as the following scaled average across all terminal branches:

$$S_\mathrm{hb} = \frac{100}{N}\sum_{i=1}^{N} s_{\mathrm{hb},i}. \tag{S20}$$

Although branch-level coverage and branch-resolved homogeneity are both computed from branch-wise deposited volumes, they play different roles. Coverage evaluates whether local deposition remains acceptable across a broad distal population, whereas branch-resolved homogeneity evaluates how closely the achieved deposition pattern approaches uniformity around the achieved mean branch dose. Branch-resolved homogeneity is therefore stricter than coverage because progressively larger branch-wise mismatches receive progressively lower scores, but less fragile than global homogeneity because the final penalty is accumulated through bounded local branch-wise scores rather than through a single outlier-sensitive global RMS deviation. Large score drops remain possible when many branches are poorly matched, but not because a small number of extreme deviations collapses the whole metric. This distinction is illustrated in S15 Fig, which compares local coverage and branch-resolved homogeneity maps in a highly asymmetric model and shows that appreciable distal coverage can coexist with substantially lower branch-resolved homogeneity, while both remain associated with very poor global homogeneity values (below 2).

Thresholds $\{\tau_k\}$ and associated weights $\{w_k\}$ used in practice are listed in S11 Table. For interpretation, a score of 130 corresponds to near-perfect delivery, with all terminal branches exhibiting relative errors below 1 %. Scores between 110 and 130 indicate highly accurate and uniform delivery, with all errors below 3 %, while scores between 90 and 110 reflect excellent overall performance, with most branch-wise deviations remaining in the 3–5 % range.

9 Limitations of homogeneity-based delivery metrics in asymmetric trees

This section provides additional quantitative support for the main-text conclusion that coverage and homogeneity-based metrics are related, but not interchangeable. In particular, homogeneity scores—especially global homogeneity—can decrease much more sharply than coverage in asymmetric trees, thereby yielding a much more pessimistic assessment of delivery quality if interpreted in isolation. To document this distinction, we compare normalized coverage and homogeneity values, their rank association across scenarios, their pediatric–adult discrimination, and the residual structure of simple bidirectional regressions.

For these analyses, raw scores were first normalized using capped reference values in order to place the different metrics on comparable scales while limiting the influence of a few extreme values. This is mainly necessary for global homogeneity, which is unbounded and can take very large values when overall deviations become very small. Coverage was capped at its natural upper bound of 100, branch-resolved homogeneity at 130, and global homogeneity at 160. For visualization of the coverage–homogeneity scatter plots, each metric was then additionally rescaled by its scenario-specific maximum so that departures from the identity line could be compared on a common relative scale.

9.1 Normalized coverage versus homogeneity scores

Normalized coverage is compared with normalized global and branch-resolved homogeneity scores in S16 Fig. In both panels, most points lie well below the identity line, showing that homogeneity-based metrics are systematically more stringent than coverage. High coverage can therefore coexist with substantially lower homogeneity, particularly in asymmetric configurations.

The two homogeneity formulations nevertheless behave differently. Branch-resolved homogeneity retains a relatively continuous relationship with coverage: although it is systematically lower, points remain distributed along a broad monotone curve, indicating that it acts mainly as a stricter version of the same large-scale delivery pattern. By contrast, global homogeneity is much more compressed toward low values. Even cases with near-maximal normalized coverage can exhibit very low normalized global homogeneity, especially in mildly and highly asymmetric settings. This confirms that global homogeneity is far more sensitive to residual imbalance and local extremes, and therefore much more likely to yield a pessimistic assessment of delivery quality.

These scatter plots are fully consistent with the main-text interpretation. Coverage primarily measures whether a broad distal population is reached, whereas homogeneity-based metrics quantify how evenly delivered volume is redistributed once access has been established. Homogeneity therefore captures a different and more demanding property of the final distribution.

9.2 Scenario-wise statistical discrimination across asymmetry levels

To quantify the relationship between coverage and homogeneity, we computed Spearman rank correlations within each asymmetry level and optimization scenario (S12 Table–S13 Table). For both branch-level and global homogeneity, the correlations are generally high. Outside the symmetric cases, where the sample size is only $n = 6$, Spearman ρ is typically very close to one: for example, $\rho \approx 0.99$ for global homogeneity in the mildly and highly asymmetric cases, and remains between about 0.95 and 0.99 for branch-resolved homogeneity. Thus, the metrics broadly preserve the same ordering of protocols: cases with higher coverage also tend to have higher homogeneity.

This strong rank association does not, however, imply interchangeability. Spearman correlation only reflects monotone ordering; it does not indicate that the metrics have the same dynamic range, the same sensitivity to local imbalance, or the same practical interpretation. This distinction is already visible in S16 Fig: homogeneity scores can collapse far more strongly than coverage while still preserving a similar ordering of cases.

We also quantified pediatric–adult discrimination using Mann–Whitney U tests within each scenario and asymmetry level. The resulting AUC values and effect sizes r confirm that both homogeneity metrics remain statistically informative. In the mildly and highly asymmetric settings, discrimination is extremely strong, with AUC values equal or very close to one and effect sizes around $r \approx 0.85$ for most comparisons. Pediatric medians are consistently higher than adult medians, as expected from the more favorable delivery conditions at pediatric scale. Nevertheless, the absolute medians show that global homogeneity is much more compressed than branch-resolved homogeneity, especially in asymmetric regimes. Homogeneity metrics therefore remain useful for distinguishing easier from harder delivery conditions, but their absolute values should be interpreted as measures of distributional uniformity rather than as direct surrogates for functional delivery success.

9.3 Regression and residual diagnostics

Regression diagnostics reinforce the same distinction between coverage and homogeneity. Using the same normalized-centered framework as in the main analysis, we fitted simple bidirectional linear models relating coverage to each homogeneity metric while accounting for subject group and asymmetry level. The key point is not the absolute fit quality, but the structure of the residuals.

For branch-resolved homogeneity, residuals remain relatively small and only weakly structured (S17 Fig). This indicates that branch-resolved homogeneity follows coverage reasonably well once subject group and geometry are taken into account. In that sense, branch-resolved homogeneity behaves as a stricter companion metric: it preserves much of the same large-scale ordering as coverage, but penalizes non-uniform branch-wise delivery more strongly.

For global homogeneity, the situation is qualitatively different. Residuals are much more dispersed and strongly structured, with a clear concentration of large deviations at comparable coverage levels. In practice, cases with similar normalized coverage can exhibit very different global-homogeneity values. This is exactly the pattern expected for a metric that is highly sensitive to residual imbalance and local outliers: coverage still captures whether distal access has been broadly achieved, but global homogeneity introduces additional variability that is not captured by coverage alone.

Taken together, these analyses show that coverage and homogeneity metrics are strongly related but not equivalent. Coverage is the primary functionally oriented metric for protocol optimization in this study because it directly reflects whether surfactant reaches a broad distal population with locally acceptable dosing. Branch-resolved homogeneity provides a complementary and stricter assessment of how evenly delivered volume is redistributed across branches, while remaining

reasonably aligned with coverage. Global homogeneity is the most stringent and the most outlier-sensitive metric, and should therefore be interpreted with particular caution, especially in adult and asymmetric geometries. Its collapse should not be read as evidence that high-coverage protocols are ineffective, but rather as evidence that the uniformity criterion has become much more severe than the access criterion when applied to the same delivery pattern.

References

1. Zheng Y, Anderson JC, Suresh V, Grotberg JB. Effect of gravity on liquid plug transport through an airway bifurcation model. J Biomech Eng. 2005;127:798-806.

2. Zheng Y, Fujioka H, Grotberg JC, Grotberg JB. Effects of inertia and gravity on liquid plug splitting at a bifurcation. J Biomech Eng. 2006;128:707-16.

3. Filoche M, Tai CF, Grotberg JB. Three-dimensional model of surfactant replacement therapy. Proc Natl Acad Sci USA. 2015;112:9287-92.

4. Collins JM, Shapiro AH, Kimmel E, Kamm RD. The steady expiratory pressure-flow relation in a model pulmonary bifurcation. J Biomech Eng. 1993;115:299-305.

5. Kazemi A, Louis B, Isabey D, Nieman GF, Gatto LA, Satalin J, et al. Surfactant delivery in rat lungs: Comparing 3D geometrical simulation model with experimental instillation. PLoS Comput Biol. 2019;15:e1007408.

6. Viquerat J, Duvigneau R, Meliga P, Kuhnle A, Hachem E. Policy-based optimization: single-step policy gradient method seen as an evolution strategy. Neural Comput Appl. 2023;35:449-67.

7. Ghraieb H, Viquerat J, Larcher A, Meliga P, Hachem E. Single-step deep reinforcement learning for two- and three-dimensional optimal shape design. AIP Adv. 2022;12:085108.

8. Michel T, Ansaldi A, Viquerat J, Meliga P, Hachem E. Combining machine learning and computational fluid dynamics for solar panel tilt angle optimization in extreme winds. Phys Fluids. 2024;36:125186.

9. Rebonato R, Jäckel P. The most general methodology to create a valid correlation matrix for risk management and option pricing purposes. Available at SSRN 1969689. 2011;135.

10. Numpacharoen K, Atsawarungruangkit A. Generating correlation matrices based on the boundaries of their coefficients. PLoS One. 2012;7:e48902.

11. Hansen N. The CMA Evolution Strategy: a tutorial. arXiv preprint arXiv:160400772. 2016.

12. Gregory TJ, Steinberg KP, Spragg R, Gadek JE, Hyers TM, Longmore WJ, et al. Bovine surfactant therapy for patients with acute respiratory distress syndrome. American journal of respiratory and critical care medicine. 1997;155:1309-15.

13. Grotberg JB, Filoche M, Willson DF, Raghavendran K, Notter RH. Did reduced alveolar delivery of surfactant contribute to negative results in adults with acute respiratory distress syndrome? Am J Respir Crit Care Med. 2017;195:538-40.
