# Supplementary material for: Reassessing adult surfactant replacement therapy with mechanics-informed reinforcement learning
Source: PLoS Comput Biol. 2026 Aug 18;22(8):e1014629. doi: 10.1371/journal.pcbi.1014629 (PMC13485052; doi:10.1371/journal.pcbi.1014629)
Supplement: Supplementary file 2 [file pcbi.1014629.s019.pdf]

Fluid	Viscosity [cP]	Density [g/cm^3]	Surface tension [dyn/cm]	No. points
LB-400-X	151	0.989	30.7	68
Water	1	1	72.4	46
Glycerin	960	1.26	63.4	7
60 % Glycerin	9.7	1.16	68.1	10
