# Supplementary material for: Reassessing adult surfactant replacement therapy with mechanics-informed reinforcement learning
Source: PLoS Comput Biol. 2026 Aug 18;22(8):e1014629. doi: 10.1371/journal.pcbi.1014629 (PMC13485052; doi:10.1371/journal.pcbi.1014629)
Supplement: Supplementary file 3 [file pcbi.1014629.s020.pdf]

| Neural network | Mean | Variance | Correlation |
|---|---|---|---|
| No. epochs | 128 | 8 | 8 |
| No. episodes per update | 1 | 8 | 16 |
| No. mini-batches | 1 | 4 | 8 |
| Learning rate | 0.005 | 0.005 | 0.001 |
| Architecture | [2,2,2] | [2,2,2] | [2,2,2] |
