# Supplementary material for: Reassessing adult surfactant replacement therapy with mechanics-informed reinforcement learning
Source: PLoS Comput Biol. 2026 Aug 18;22(8):e1014629. doi: 10.1371/journal.pcbi.1014629 (PMC13485052; doi:10.1371/journal.pcbi.1014629)
Supplement: Supplementary file 4 [file pcbi.1014629.s021.pdf]

| Parameter | Value | Role |
|---|---|---|
| $x_{\text{min}}$ | 0.3 | Lower threshold for nonzero local coverage credit |
| $x_{\text{max}}$ | 1.4 | Upper tolerance range for full local coverage credit |
| $k_{\text{over}}$ | 3.0 | Strength of local overdosing penalty |
| $x_{\text{u,min}}$ | 0.75 | Lower threshold for conditional uniformity subset |
| $x_{\text{u,max}}$ | 1.25 | Upper threshold for conditional uniformity subset |
| $K_{\text{over}}$ | 1.5 | Strength of global overvolume penalty |
| $f_d$ | 0.75 | Distal target fraction |
