# Supplementary material for: Reassessing adult surfactant replacement therapy with mechanics-informed reinforcement learning
Source: PLoS Comput Biol. 2026 Aug 18;22(8):e1014629. doi: 10.1371/journal.pcbi.1014629 (PMC13485052; doi:10.1371/journal.pcbi.1014629)
Supplement: Supplementary file 5 [file pcbi.1014629.s022.pdf]

Group	Variant	$x_{\min}$	$x_{\max}$	k_{over}	$x_{\text{u,min}}$	$x_{\text{u,max}}$	K_{over}	f_d
Reference	ref	0.3	1.4	3.0	0.75	1.25	1.5	0.75
Coverage	cov-loose	0.2	1.5	2.0	0.75	1.25	1.5	0.75
	cov-tight	0.4	1.3	4.0	0.75	1.25	1.5	0.75
Cond. uniformity	uni-loose	0.3	1.4	3.0	0.70	1.30	1.5	0.75
	uni-tight	0.3	1.4	3.0	0.80	1.20	1.5	0.75
Distal dose	dist-loose	0.3	1.4	3.0	0.75	1.25	1.5	0.70
	dist-tight	0.3	1.4	3.0	0.75	1.25	1.5	0.80
