# Supplementary material for: Reassessing adult surfactant replacement therapy with mechanics-informed reinforcement learning
Source: PLoS Comput Biol. 2026 Aug 18;22(8):e1014629. doi: 10.1371/journal.pcbi.1014629 (PMC13485052; doi:10.1371/journal.pcbi.1014629)
Supplement: Supplementary file 6 [file pcbi.1014629.s023.pdf]

Scenario	Reward	Scores			Policy alignment	
	r	S_c	$100S_e$	$100S_u$	All	No posture
Baseline	57.5 [0.0]	74.1 [0.0]	88.0 [0.0]	88.3 [0.0]	1.000 [0.000]	—
Per-dose	60.0 [0.1]	76.9 [0.1]	88.0 [0.0]	88.7 [0.0]	0.800 [0.101]	—
Posture	60.1 [0.5]	76.9 [1.0]	87.6 [0.2]	89.0 [0.4]	0.023 [0.512]	0.849 [0.085]
Rheology	65.0 [0.3]	79.3 [0.9]	92.7 [0.4]	88.5 [0.2]	0.768 [0.168]	0.884 [0.097]
