# Supplementary material for: Reassessing adult surfactant replacement therapy with mechanics-informed reinforcement learning
Source: PLoS Comput Biol. 2026 Aug 18;22(8):e1014629. doi: 10.1371/journal.pcbi.1014629 (PMC13485052; doi:10.1371/journal.pcbi.1014629)
Supplement: Supplementary file 7 [file pcbi.1014629.s024.pdf]

Scenario	Variant	Rel. variations (%)				Policy alignment	
		r	S_c	S_e	S_u	All	No posture
Posture	cov-loose	+8.2 [1.5]	+8.5 [2.6]	0.5 [0.4]	-0.3 [0.7]	0.376 [0.375]	0.943 [0.087]
	cov-tight	-9.7 [0.8]	-9.2 [1.6]	-0.1 [0.3]	-0.7 [1.0]		
	uni-loose	-2.2 [1.5]	0.2 [2.4]	0.1 [0.5]	-2.4 [0.8]	0.550 [0.433]	0.955 [0.124]
	uni-tight	+1.8 [0.6]	-0.2 [1.6]	0.0 [0.1]	+2.1 [1.0]		
	dist-loose	-1.0 [1.1]	0.3 [2.1]	-0.5 [0.5]	-0.4 [0.7]	0.389 [0.211]	0.974 [0.185]
	dist-tight	-0.7 [1.2]	0.2 [2.0]	0.3 [0.4]	-1.0 [0.9]		
