# Supplementary material for: Reassessing adult surfactant replacement therapy with mechanics-informed reinforcement learning
Source: PLoS Comput Biol. 2026 Aug 18;22(8):e1014629. doi: 10.1371/journal.pcbi.1014629 (PMC13485052; doi:10.1371/journal.pcbi.1014629)
Supplement: Supplementary file 8 [file pcbi.1014629.s025.pdf]

Scenario	Reward	Scores			Policy alignment	
	r	S_c	$100S_e$	$100S_u$	All	No posture
Posture	38.2 [1.1]	57.8 [0.8]	75.7 [1.2]	87.3 [0.0]	0.293 [0.273]	0.996 [0.004]
