# Supplementary material for: Reassessing adult surfactant replacement therapy with mechanics-informed reinforcement learning
Source: PLoS Comput Biol. 2026 Aug 18;22(8):e1014629. doi: 10.1371/journal.pcbi.1014629 (PMC13485052; doi:10.1371/journal.pcbi.1014629)
Supplement: Supplementary file 9 [file pcbi.1014629.s026.pdf]

Scenario	Variant	Rel. variations (%)				Policy alignment	
		r	S_c	S_e	S_u	All	No posture
Posture	cov-loose	+12.4 [6.1]	+13.0 [2.7]	-0.6 [2.3]	0.0 [0.1]	0.544 [0.298]	0.997 [0.005]
	cov-tight	-16.3 [5.0]	-14.4 [2.3]	-0.9 [3.0]	0.0 [0.0]		
	uni-loose	-2.4 [3.6]	0.0 [1.8]	+0.1 [1.8]	-2.5 [0.0]	0.293 [0.732]	0.999 [0.007]
	uni-tight	+5.4 [6.1]	+1.5 [2.6]	+1.1 [3.2]	+2.7 [0.0]		
	dist-loose	+2.3 [3.5]	+2.3 [1.6]	0.0 [2.1]	+0.1 [0.0]	0.535 [0.293]	0.995 [0.007]
	dist-tight	-2.5 [5.5]	-2.3 [2.2]	-0.2 [2.3]	0.0 [0.0]		
