# Supplementary material for: Reassessing adult surfactant replacement therapy with mechanics-informed reinforcement learning
Source: PLoS Comput Biol. 2026 Aug 18;22(8):e1014629. doi: 10.1371/journal.pcbi.1014629 (PMC13485052; doi:10.1371/journal.pcbi.1014629)
Supplement: Supplementary file 10 [file pcbi.1014629.s027.pdf]

Scenario	Group	Target vol. [mL/kg]	No. aliquots		
			2×	4×	8×
Baseline	Pediatric	1	86.3 [4.7]	89.0 [3.5]	93.4 [1.7]
		2	90.8 [2.2]	91.9 [2.1]	94.1 [2.0]
		3	90.1 [2.3]	91.3 [2.4]	92.1 [2.8]
		4	90.3 [2.1]	91.0 [1.9]	92.1 [1.5]
	Adult	1	20.0 [15.9]	53.3 [9.0]	61.5 [2.1]
		2	65.2 [1.2]	69.4 [4.0]	70.4 [3.5]
		3	70.6 [2.7]	71.8 [1.6]	75.0 [2.2]
		4	74.5 [2.3]	76.8 [1.6]	79.7 [1.8]
