# Supplementary material for: Reassessing adult surfactant replacement therapy with mechanics-informed reinforcement learning
Source: PLoS Comput Biol. 2026 Aug 18;22(8):e1014629. doi: 10.1371/journal.pcbi.1014629 (PMC13485052; doi:10.1371/journal.pcbi.1014629)
Supplement: Supplementary file 11 [file pcbi.1014629.s028.pdf]

Scenario	Group	Target vol. [mL/kg]	No. aliquots		
			2×	4×	8×
Per-dose	Pediatric	2	92.0 [2.1]	94.0 [1.4]	95.5 [1.6]
	Adult	4	78.0 [2.0]	81.1 [1.5]	84.3 [1.6]
Posture	Pediatric	2	94.2 [0.6]	95.9 [0.8]	97.9 [0.0]
	Adult	4	80.4 [1.1]	84.0 [0.2]	86.2 [0.6]
Rheology	Pediatric	2	94.5 [0.6]	97.7 [0.1]	99.3 [0.1]
	Adult	4	82.3 [0.8]	85.8 [0.2]	88.1 [0.6]
