# Supplementary material for: Reassessing adult surfactant replacement therapy with mechanics-informed reinforcement learning
Source: PLoS Comput Biol. 2026 Aug 18;22(8):e1014629. doi: 10.1371/journal.pcbi.1014629 (PMC13485052; doi:10.1371/journal.pcbi.1014629)
Supplement: Supplementary file 12 [file pcbi.1014629.s029.pdf]

Threshold τ_k	0.01	0.02	0.03	0.04	0.05	0.06	0.08	0.10
Weight w_k	1.3	1.2	1.1	1.0	0.9	0.7	0.4	0.1
