# Supplementary material for: Reassessing adult surfactant replacement therapy with mechanics-informed reinforcement learning
Source: PLoS Comput Biol. 2026 Aug 18;22(8):e1014629. doi: 10.1371/journal.pcbi.1014629 (PMC13485052; doi:10.1371/journal.pcbi.1014629)
Supplement: Supplementary file 13 [file pcbi.1014629.s030.pdf]

Model	Scenario	Spearman	AUC	r	Median	
					Pediatric	Adult
Sym.	Baseline	0.66	0.67	0.27	0.78	0.61
	Per-dose	0.94	1.00	0.80	0.59	0.08
	Posture	0.94	1.00	0.80	0.65	0.09
	Rheology	0.94	0.89	0.62	0.60	0.22
Mild asym.	Baseline	0.99	1.00	0.85	0.29	0.19
	Per-dose	0.99	1.00	0.85	0.04	0.02
	Posture	0.99	1.00	0.85	0.05	0.03
	Rheology	0.99	1.00	0.85	0.12	0.06
High asym.	Baseline	0.99	1.00	0.85	0.15	0.10
	Per-dose	0.99	1.00	0.85	0.02	0.01
	Posture	0.99	1.00	0.85	0.03	0.02
	Rheology	0.99	1.00	0.85	0.07	0.03
