# Supplementary material for: Reassessing adult surfactant replacement therapy with mechanics-informed reinforcement learning
Source: PLoS Comput Biol. 2026 Aug 18;22(8):e1014629. doi: 10.1371/journal.pcbi.1014629 (PMC13485052; doi:10.1371/journal.pcbi.1014629)
Supplement: Supplementary file 14 [file pcbi.1014629.s031.pdf]

Model	Scenario	Spearman	AUC	r	Median	
					Pediatric	Adult
Sym.	Baseline	0.66	0.67	0.27	0.83	0.69
	Per-dose	0.94	1.00	0.80	0.98	0.57
	Posture	0.94	1.00	0.80	0.98	0.52
	Rheology	0.94	0.89	0.62	0.93	0.62
Mild asym.	Baseline	0.98	1.00	0.85	0.31	0.21
	Per-dose	0.98	1.00	0.85	0.25	0.15
	Posture	0.97	1.00	0.85	0.27	0.17
	Rheology	0.99	1.00	0.85	0.34	0.18
High asym.	Baseline	0.98	1.00	0.85	0.17	0.11
	Per-dose	0.95	0.99	0.84	0.13	0.08
	Posture	0.97	1.00	0.85	0.14	0.09
	Rheology	0.99	1.00	0.85	0.21	0.10
