# Supplementary material for: Reassessing adult surfactant replacement therapy with mechanics-informed reinforcement learning
Source: PLoS Comput Biol. 2026 Aug 18;22(8):e1014629. doi: 10.1371/journal.pcbi.1014629 (PMC13485052; doi:10.1371/journal.pcbi.1014629)
Supplement: Supplementary file 15 [file pcbi.1014629.s032.pdf]

Require: unit direction vectors for the parent ($\mathbf{v}_0$) and daughter airways ($\mathbf{v}_1$, $\mathbf{v}_2$); global vertical unit vector $\mathbf{e}_z$

compute the horizontal projection of the parent direction: $\mathbf{v}_{0//} = \mathbf{v}_0 - (\mathbf{v}_0 \cdot \mathbf{e}_z)\mathbf{e}_z$
if $\|\mathbf{v}_{0//}\| = 0$ **then**
    $\gamma = \frac{\pi}{2}\,\mathrm{sgn}(\mathbf{v}_0 \cdot \mathbf{e}_z)$
else
    normalize $\mathbf{v}_{0//} \leftarrow \mathbf{v}_{0//}/\|\mathbf{v}_{0//}\|$
    compute γ from $\cos\gamma = \mathbf{v}_{0//} \cdot \mathbf{v}_0$ and $\sin\gamma = \mathbf{v}_0 \cdot \mathbf{e}_z$
compute the unit normal to the bifurcation plane: $\mathbf{v}_n = (\mathbf{v}_1 \times \mathbf{v}_2)/\|\mathbf{v}_1 \times \mathbf{v}_2\|$
set rotation axis $\mathbf{v}_\gamma = \mathbf{v}_0 \times \mathbf{e}_z$
if $\|\mathbf{v}_\gamma\| = 0$ **then**
    $\mathbf{v}'_n = \mathbf{v}_n$ ▷ degenerate case: parent axis aligned with gravity
else
    rotate $\mathbf{v}_n$ by angle $-\gamma$ around axis $\mathbf{v}_\gamma$ to obtain $\mathbf{v}'_n$
compute the horizontal projection: $\mathbf{v}'_{n//} = \mathbf{v}'_n - (\mathbf{v}'_n \cdot \mathbf{e}_z)\mathbf{e}_z$
if $\|\mathbf{v}'_{n//}\| = 0$ **then**
    $\phi = 0$
else
    normalize $\mathbf{v}'_{n//} \leftarrow \mathbf{v}'_{n//}/\|\mathbf{v}'_{n//}\|$
    compute $s = \mathrm{sgn}\big((\mathbf{e}_z \times \mathbf{v}'_{n//}) \cdot \mathbf{v}'_n\big)$
    compute ϕ from $\cos\phi = \mathbf{v}'_n \cdot \mathbf{e}_z$ and $\sin\phi = s\,(\mathbf{v}'_{n//} \cdot \mathbf{v}'_n)$
