# Supplementary material for: Reassessing adult surfactant replacement therapy with mechanics-informed reinforcement learning
Source: PLoS Comput Biol. 2026 Aug 18;22(8):e1014629. doi: 10.1371/journal.pcbi.1014629 (PMC13485052; doi:10.1371/journal.pcbi.1014629)
Supplement: Supplementary file 16 [file pcbi.1014629.s033.pdf]

Require: Airway-tree geometry G; mechanistic SRT simulator $S(\cdot,\cdot)$; policy π_θ over $a \in [-1,1]^d$; action-to-protocol map $\Phi(\cdot)$; quantization operator $\mathcal{Q}(\cdot)$; metric extractor $\mathcal{M}(\cdot)$; reward function $R(\cdot)$; number of training iterations N_{ep}; number of parallel environments n_{env}.

Ensure: Trained policy parameters θ.

function ENVSTEP(a, G)
    $p_{\text{cont}} \leftarrow \Phi(a)$ ▷ Map to physical ranges (volumes, flows, posture, rheology)
    $p \leftarrow \mathcal{Q}(p_{\text{cont}})$ ▷ Apply predefined discretization/step sizes
    $y \leftarrow S(G, p)$ ▷ Simulate full protocol; terminal outcome
    $m \leftarrow \mathcal{M}(y)$ ▷ Coverage/auxiliary delivery metrics
    $r \leftarrow R(m)$ ▷ Scalar reward
    return (r, m, p)
Initialize θ
for $t = 1$ to N_{ep} **do**
    Sample a batch of actions $\{a_i\}_{i=1}^{n_{\text{env}}} \sim \pi_\theta$ ▷ One-step, stateless episodes
    for $i = 1$ to n_{env} **do** ▷ Parallel execution
        $(r_i, m_i, p_i) \leftarrow$ ENVSTEP(a_i, G) ▷ Update uses r_i; (m_i, p_i) for logging
    $\theta \leftarrow$ UPDATEPBO$(\theta, \{a_i\}, \{r_i\})$ ▷ Policy update; see S3 Algorithm
return θ
