# Supplementary material for: Reassessing adult surfactant replacement therapy with mechanics-informed reinforcement learning
Source: PLoS Comput Biol. 2026 Aug 18;22(8):e1014629. doi: 10.1371/journal.pcbi.1014629 (PMC13485052; doi:10.1371/journal.pcbi.1014629)
Supplement: Supplementary file 17 [file pcbi.1014629.s034.pdf]

Require: Batch of actions $\{a_i\}_{i=1}^{n_{\text{env}}}$ and rewards $\{r_i\}_{i=1}^{n_{\text{env}}}$ (from S2 Algorithm); internal history buffer $\mathcal{H}$ (persisting across calls); history length n_h; decay factor η; history-to-dataset operator $\mathcal{D}_\eta(\cdot)$; reward normalization operator $\mathcal{W}(\cdot)$; mini-batch operator $\mathcal{B}(\cdot,\cdot)$; network-specific training hyperparameters (n_e, n_b, λ_r).

Ensure: Trained policy parameters $\theta = \{\theta_\mu, \theta_\sigma, \theta_\rho\}$.

function UpdatePBO($\theta, \{a_i\}, \{r_i\}$)
    $\mathcal{H} \leftarrow$ Push($\mathcal{H}, \{a_i\}, \{r_i\}, n_h$) ▷ Append batch; keep last n_h iterations
    $\mathcal{D} \leftarrow \mathcal{D}_\eta(\mathcal{H})$ ▷ Build decayed training set
    $\{\tilde{r}_j\} \leftarrow \mathcal{W}(\{r_j\}_{(a_j,r_j)\in\mathcal{D}})$ ▷ Whiten rewards
    for $q \in \{\mu, \sigma, \rho\}$ **do** ▷ Use (n_e, n_b, λ_r) specific to network q
        for $e = 1$ to n_e **do**
            shuffle $\mathcal{D}$ ▷ Randomize before batching
            for $k = 1$ to n_b **do**
                $\mathcal{B} \leftarrow \mathcal{B}(\mathcal{D}, k)$ ▷ Form mini-batch (fixed size)
                $\hat{L}_q \leftarrow \frac{1}{|\mathcal{B}|}\sum_{(a_j,\tilde{r}_j)\in\mathcal{B}} \max(\tilde{r}_j, 0)\, \log \pi_\theta(a_j)$ ▷ Mini-batch estimate of the PBO loss
                $\theta_q \leftarrow$ AdamStep($\theta_q, \nabla_{\theta_q}\hat{L}_q, \lambda_r$) ▷ Policy update
    return θ
